# α-Bromodiazoacetamides – a new class of diazo compounds for catalyst-free, ambient temperature intramolecular C–H insertion reactions

**DOI:** 10.3762/bjoc.9.157

**Published:** 2013-07-11

**Authors:** Åsmund Kaupang, Tore Bonge-Hansen

**Affiliations:** 1 Department of Chemistry, University of Oslo, P.O. Box 1033 Blindern, NO-0315 Oslo, Norway

**Keywords:** α-halo-β-lactam, diazo, halocarbonylcarbene, halogenation, thermolysis

## Abstract

In this work, we introduce a new class of halodiazocarbonyl compounds, α-halodiazoacetamides, which through a metal-free, ambient-temperature thermolysis perform intramolecular C–H insertions to produce α-halo-β-lactams. When carried out with α-bromodiazoacetamides bearing cyclic side chains, the thermolysis reaction affords bicyclic α-halo-β-lactams, in some cases in excellent yields, depending on the ring size and substitution pattern of the cyclic amide side chains.

## Introduction

Diazocarbonyl compounds are popular precursors for carbonylcarbenes and -carbenoids, the synthetic utility of which is thoroughly established through their successful employment in cycloaddition, ylide formation, cyclopropanation and C–H insertion reactions [1–3]. A generally useful modification of diazo compounds is the substitution of the α-hydrogen for an electrophile. This substitution can be effected in the presence of a base or starting from the metalated diazo compound, and leaves the diazo function intact [4]. Among the reported transformations are substitutions of the diazomethyl hydrogen for electrophiles based on boron [5–7], nitrogen (NO_2_^+^) [8–12], silicon [13–15], phosphorous [16–18], sulfur [19–21] and halogens [10,22–30], as well as carbon, e.g., in aldol reactions with aldehydes [31–32], ketones [33–34] and imines [35–36].

The first syntheses of α-halodiazoacetic esters, reported in the late 1960s, employed electrophilic diazoalkane substitution; the mercury or silver salts of ethyl diazoacetate (EDA) were reacted with sources of electrophilic halogen (SO_2_Cl_2_, Br_2_ or I_2_). These protocols allowed Gerhart and Schöllkopf et al. to study the properties of the resulting α-halodiazoacetates and the reactivity of their photolytically derived carbenes [26–30]. Some ten years later, Regitz et al. reported the syntheses of an α-halodiazomethyl phosphonic acid dimethyl ester and an α-halodiazomethyl diphenyl phosphoxide, starting from the silver salts of the respective diazo compounds [10].

More recently, two novel protocols for the halogenation of diazoesters and -phosphonates have been introduced by our group, both employing an *N*-halosuccinimide as the halogen source in combination with either the amidine base 1,8-diazabicyclo[5.4.0]undec-7-ene (DBU) or sodium hydride (NaH). In these reports, the obtained α-halodiazoacetates and α-halodiazophosphonates were successfully applied in dirhodium(II)-catalysed cyclopropanation, and C–H and Si–H insertion reactions [37–39].

There are, to the best of our knowledge, no reports in the literature of α-halodiazoacetamides as a substance class. Thus, we wished to expand the substrate scope of one of our published methodologies to encompass the halogenation of diazoacetamides. We report herein the bromination of the diazoacetamides derived from a selection of cyclic secondary amines, using DBU and *N*-bromophthalimide (NBP), as well as an investigation of the ability of the carbenes/carbenoids derived from the resulting α-bromodiazoacetamides to form α-bromo-β-lactams.

## Results

**Scheme 1:**
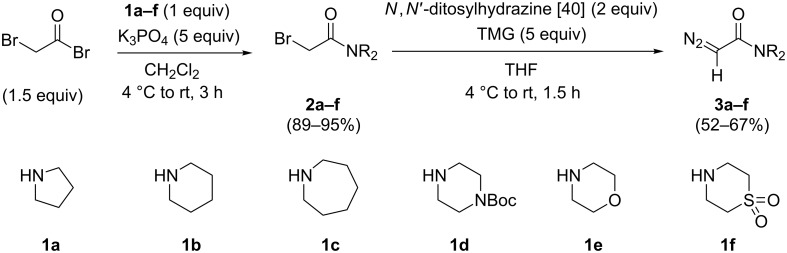
Preparation of the diazoacetamides.

The diazoacetamides **3a**–**f** were synthesised from α-bromoacetamides **2a**–**f** using a protocol published by Toma et al. [40], modified by exchanging the base employed in the original procedure (DBU) for 1,1,3,3-tetramethylguanidine (TMG). The use of TMG allowed for a more convenient, nonaqueous workup, involving the near quantitative removal of the produced TMG-*p*-toluenesulfinate salt [41] by filtration of a diethyl ether dispersion of the crude reaction mixture. This modification thus allowed for gram-scale preparations of the desired diazoacetamides. The α-bromoacetamides were in turn prepared by the acylation of the respective secondary amines **1a**–**f** with bromoacetyl bromide (Scheme 1; see Supporting Information File 1 for full experimental details). Among the obtained diazoacetamides, **3d** and **3f** were not previously reported. We therefore prepared crystals and resolved their structures by single-crystal X-ray diffraction. The diazoacetamides **3d** and **3f** crystallised as their (*Z*)-rotamers. These data have recently been reported [42–43].

**Scheme 2:**
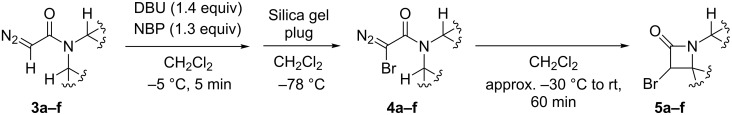
Bromination of the diazoacetamides **3a**–**f** and thermolysis of the α-bromodiazoacetamides **4a**–**f**.

The diazoacetamides were brominated at −5 °C with NBP in the presence of DBU and passed through a dry-ice-cooled plug of silica gel with CH_2_Cl_2_ (precooled to −15 °C), in order to remove the base and phthalimide. Allowing the solution to warm to ambient temperature effected the thermolysis of the α-bromodiazoacetamides (Scheme 2). Although we have not determined the exact temperature at which the thermolysis takes place, the α-bromodiazoacetamides will rapidly lose their bright red colour at temperatures above 0 °C.

As can be seen in Table 1, the obtained yields of the α-bromo-β-lactams **5a**–**f** vary significantly throughout the series. Among the derivatives with aliphatic amide side chains, the yields increase dramatically with ring size (Table 1, entries 1–3, products **5a**–**c**), whereas among the derivatives bearing 1,4-heterosubstituted six-membered rings as side chains, poorer yields are obtained (Table 1, entries 4–6, products **5d**–**f**). The latter result could possibly be viewed as an expression of the deactivation of the C–H bonds β to the *N*-methylene groups of the amide [44]. With the exception of the piperazine derivative **5d**, the observed diastereomeric ratio in the β-lactam products was approximately 6:1, favouring the diastereomer in which the bromine atom and the ring fragment are in a *trans* relationship (hereafter referred to as *exo*-**5a**–**f**). The stereochemistry of the obtained β-lactams was determined based on the previously published NMR data for *endo*/*exo*-**5b** [45], as well as on the characteristic magnitude of the couplings and chemical shift values of the α-protons of the *endo*/*exo* stereoisomers [46–49].

**Table 1:** Yields^a^ of β-lactams **5a**–**f** obtained by thermolysis of α-bromodiazoacetamides **4a**–**f**.

		Yields^a^ (%)				
Entry	Product	*exo*	*endo*	*exo* + *endo*	*exo*/*endo*	α,α’-dibromoacetamide

1	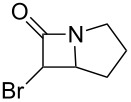 **5a**	7^b^	–^c^	7^b^	n/a	34
2	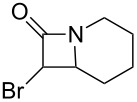 **5b**	73	11	84	7:1	2
3	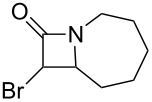 **5c**	77 (81)^d^	16 (13)^d^	93 (94)^d^	5:1 (6:1)^d^	trace (–)
4	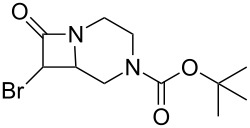 **5d**	34	2	36	17:1	21
5	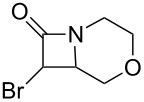 **5e**	12	1	13	6:1	20
6	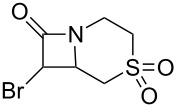 **5f**	14	3	17	5:1	12

^a^Determined by ^1^H NMR using an internal standard (see Supporting Information File 1 for details). ^b^Decomposed in CDCl_3_ within 48 hours (see Supporting Information File 1 for details). ^c^Not detected by ^1^H NMR. ^d^Isolated yield after chromatography. Bromination performed with NBS/DBU.

The high-yielding C–H insertions (Table 1, entries 2–3, products **5b**,**c**) proceed cleanly and with few byproducts (see the NMR spectra of the crude reaction mixtures containing **5b**,**c** in Supporting Information File 1). In contrast, in the lower yielding reactions, a byproduct that could be routinely identified was the corresponding α,α'-dibromoacetamide, the origin of which remains unclear. Due to the overlap of signals from the amide side chains in the starting materials and those of eventual dimeric products, the extent of formation of carbene dimers was not possible to determine from the crude ^1^H NMR spectra.

As can be interpreted from Table 1, the dominant reaction pathway in the high-yielding reactions (Table 1, entries 2–3), is apparently the intramolecular C–H insertion to form a β-lactam. The preferential formation of intramolecular products from *N*,*N*’-disubstituted diazoacetamides, as compared to diazoacetates has previously been rationalised in terms of the proximity of the side chain C–H bonds to the carbene centre [50–52]. The greater ease with which the α-bromocarbene amide can insert into the C–H bonds of the larger rings (cf. the increase in yields of products **5a**, **5b** and **5c**) suggests that the proximity and/or conformational flexibility of the C–H fragment is of importance.

The α-bromo-β-lactam **5b** and its α-chloro-analogue have previously been prepared by Johansson et al. in 61% [45] and 53–54% yield [53–54], respectively, by the thermolysis of α-dihalo(phenylmercury)acetamides in bromobenzene under reflux: a reaction that plausibly occurs with a free halocarbonylcarbene or a mercury carbenoid as intermediate [45,55–56]. In the case of the α-bromo-β-lactam **5b**, the authors reported an *exo-*diastereoselectivity of 5.25:1. In order to compare the reactivity of other halides in the α-position, we prepared and thermolysed the α-chloro- and α-iodo analogues of **4d** (see results in Table 5.1 in Supporting Information File 1) [57]. In analogy to the results of Johansson et al., the α-chloro analogue afforded a lower yield of the corresponding α-chloro-β-lactam. In the case of the α-iodo analogue, the surprisingly low yield obtained may be due to decomposition during the chromatographic step, as indicated by a change in colour from red to purple, possibly owing to the formation of I_2_.

In a broader context, related examples of carbene/carbenoid C–H insertions to form β-lactams exist in the literature, in which the α-substituent on the carbene carbon varies (see below). The α-phenyl analogue of β-lactam **5b** has previously been prepared by carbene C–H insertion. In these reports, the base-promoted decomposition of a hydrazone and subsequent thermolysis of the diazo compound in, e.g., toluene under reflux, afforded the bicyclic α-phenyl-β-lactam in up to 60% yield (6:1/*exo:endo*) [58–60]. Axten et al. also prepared the α-phenyl-analogues of **5a**, **5c** and **5e**, as well as an azocane (heptamethyleneimine) derivative. The yields of the obtained β-lactams were, however, not reported [60].

### Dirhodium(II) catalysis

Comparing with results obtained using dirhodium(II) catalysis, the α-H-analogue of the azepane derivative **5c** was prepared in 67% yield by Doyle et al. from **3d** [61]. They could also prepare the analogous azocane-derived α-H-β-lactam in 45% yield, accompanied by a 22% yield of the α-H-γ-lactam. Interestingly, the dirhodium(II)-catalysed methylene C–H insertion reactions of the smaller cyclic derivatives **3a**, **3b** and **3e**, were unsuccessful [61]. For comparison with the thermolytic reaction, we tested the performance of a small series of dirhodium(II)-catalysts in the intramolecular C–H insertion that forms **5b**. Our best result was obtained with the electron-rich dirhodium(II) carboxamidate Rh_2_(cap)_4_, affording a 44% combined yield with a 6:1 *exo*/*endo* ratio (versus 84% combined yield, 7:1 *exo*/*endo*, in the thermolysis; see Table 5.2 in Supporting Information File 1 for details). Furthermore, we observed a correlation between the electron-donating ability of the dirhodium(II) catalyst and the obtained yield of the β-lactam **5d** (see Table 5.2 in Supporting Information File 1). In our hands, the more stabilised carbenoid afforded the best result, suggesting that the intramolecular C–H insertion reaction was favoured by a less reactive carbenoid. This result may provide insight into why the formation of the bicyclic system is achieved starting from the α-*bromo*diazoacetamide **4c**, but not from the α-H diazoacetamide **3c** [61] (see Discussion on the carbene-stabilising effect of halogens below) employing an electronically comparable dirhodium(II) carboxamidate catalyst (Rh_2_(cap)_4_ versus Rh_2_(*S*-MEPY)_4_).

## Discussion

### Thermolysis

To the best of our knowledge, only one previous account of the synthetic application of a thermolysis of a halodiazocarbonyl compound can be found in the literature. In this report the thermolysis of an α-bromodiazoketone was successfully employed in an intramolecular cyclopropanation reaction [62]. Historically, the thermolysis of diazocarbonyl compounds has been carried out under reflux [63–66], although examples of low temperature and ambient temperature thermolysis can be found in the case of more labile diazo compounds [14,22–23,67]. Recent examples of thermolyses of diazocarbonyl compounds, include the preparation of arylcyclopropanes (cyclopropanation) and α-arylamino esters (N–H insertion) by thermolysis of aryldiazoacetates in trifluorotoluene under reflux [68–69]. In terms of their application in synthesis, the need for prolonged heating may have narrowed the substrate scope of thermolysis reactions considerably, contributing to the limited number of reports of catalyst-free thermolyses of diazo compounds in the literature. In comparison, the capability of the α-bromodiazoacetamides to thermolyse at ambient temperature without the use of a metal catalyst, offers the reactivity of the bromocarbene amides under genuinely mild reaction conditions.

### Halocarbonyl carbenes and carbene/carbenoid stabilisation

In their ground state, the halocarbonyl carbenes derived from halodiazoamides are, in analogy with those derived from halodiazoesters, predicted by theory to be singlet carbenes [70]. Furthermore, in analogy to α-bromoethoxycarbonyl carbene [28,30,71], they should be electrophilic carbenes. An important modulator of carbene electrophilicity is the stabilising effect of π-donation from a substituent on the carbene carbon into the vacant carbene 2p-orbital. This effect is predicted by theory, as well as observed experimentally [28,30,71–73]. The degree of stabilisation exerted by a bromine substituent has been quantified in the scale of carbenic reactivity, introduced by R. A. Moss [71]. The increased stabilisation of α-bromocarboethoxycarbene as compared to α-H-carboethoxycarbene, is apparent in its increased propensity towards reaction with the more electron-rich of the available reaction partners, a propensity which has traditionally been quantified by the ratios of cyclopropanation of increasingly substituted ethylenes [28,30,71]. More recently, a two-dimensional carbenic reactivity surface has been established by Brinker and co-workers. Unfortunately, no halocarbonylcarbene has yet been included in these studies [73].

The donation of electron density to the vacant carbenoid 2p orbital, has also been a topic in the paradigm of dirhodium(II) catalysis, in which the electron-donating ability of the catalyst ligands correlates to the observed selectivity of the dirhodium(II) carbenoid. Thus, in analogy to the preference for more highly substituted ethylenes, in a case where only C–H insertion is viable, a highly stabilised dirhodium(II) carbenoid shows increased selectivity among the possible C–H insertion partners. For alkanes, the following order of preference is observed: R_3_CH > R_2_CH_2_ > RCH_3_ [2,74–76]. The order of reactivity of the C–H bonds reflects the varying abilities of the CH_n_ carbons to stabilise the build-up of positive charge as the incipient electrophilic carbene/carbenoid 2p orbital attacks the C–H σ-bonding orbital of the substrate. Consequently, C–H bonds vicinal to heteroatoms are activated for insertion [44,77]. The observed selectivity of the more stabilised carbenoids is attributed to the donation of electron density from the 4d orbitals of the dirhodium(II) complex to the vacant carbenoid 2p orbital [76,78–81], which attenuates its electrophilicity. This effect is analogous to the increased selectivity conferred by π-donation from a halogen substituent on the carbene carbon (see above) [28,30,71,81–84].

### Future perspectives

A pronounced goal in the previously published syntheses of the piperidine-derived bicyclic β-lactams, analogues of **5b**, was to develop a model system for the synthesis of β-lactam antibiotics [58,85]. In this vein, the metal-independent character of the presented thermolysis of α-bromodiazoacetamides makes the transformation compatible with late-stage synthesis in medicinal chemistry. Additionally, in a preparative context, the formation of α-bromo-β-lactams such as **5b,c** using thermolysis is advantageous; **5c** could be conveniently prepared in excellent isolated yields from **3c**, without the use of expensive catalysts, inert atmosphere or anhydrous conditions.

## Conclusion

We have demonstrated the halogenation of a series of diazoacetamides and the ambient temperature thermolysis of the resulting α-halodiazoacetamides. The synthetic utility of the α-bromodiazoacetamides has been demonstrated in the preparation of bicyclic α-bromo-β-lactams. In the cases where C–H insertion is conformationally favoured, the α-bromo-β-lactams were obtained in good to excellent yields.

## Supporting Information

File 1 Detailed experimental procedures and physical data for the obtained products.
