# Supplementary material for: α-Bromodiazoacetamides – a new class of diazo compounds for catalyst-free, ambient temperature intramolecular C–H insertion reactions
Source: Beilstein J Org Chem. 2013 Jul 11;9:1407–13. doi: 10.3762/bjoc.9.157 (PMC3740503; doi:10.3762/bjoc.9.157)

Supporting Information

for

α-Bromodiazoacetamides – a new class of diazo compounds for catalyst-free, ambient temperature intramolecular C–H insertion reactions

Åsmund Kaupang and Tore Bonge-Hansen*

Address: Department of Chemistry, University of Oslo, P.O. Box 1033 Blindern, NO-0315 Oslo, Norway

Email: Åsmund Kaupang – asmund.kaupang@farmasi.uio.no; Tore Bonge-Hansen* - tore.hansen@kjemi.uio.no

*Corresponding author

Detailed experimental procedures and physical data for the obtained products

Contents

Contents 2

General introduction 4

Experimental procedures 5

1 *N*-bromoimides 5

1.1 N-bromophthalimide 5

1.2 N-bromosuccinimide 6

2 *N,N'*-ditosylhydrazine 6

3 α-bromoacetamides 8

3.1 2-bromo-1-(pyrrolidin-1-yl)ethanone (**2a**) 8

3.2 2-bromo-1-(piperidin-1-yl)ethanone (**2b**) 9

3.3 1-(azepan-1-yl)-2-bromoethanone (**2c**) 9

3.4 tert-butyl 4-(2-bromoacetyl)piperazine-1-carboxylate (**2d**) 10

3.5 2-bromo-1-morpholinoethanone (**2e**) 10

3.6 2-bromo-1-(1,1-dioxidothiomorpholino)ethanone (**2f**) 11

4 α-diazoacetamides 11

4.1 2-diazo-1-(pyrrolidin-1-yl)ethanone (**3a**) 12

4.2 2-diazo-1-(piperidin-1-yl)ethanone (**3b**) 13

4.3 1-(azepan-1-yl)-2-diazoethanone (**3c**) 13

4.4 tert-butyl 4-(2-diazoacetyl)piperazine-1-carboxylate (**3d**) 14

4.5 2-diazo-1-morpholinoethanone (**3e**) 15

4.6 2-diazo-1-(1,1-dioxido-4-thiomorpholinyl)ethanone (**3f**) 15

5 β-lactams 16

5.1 6-bromo-1-azabicyclo[3.2.0]heptan-7-one (**5a**) 18

5.2 7-bromo-1-azabicyclo[4.2.0]octan-8-one (**5b**) 18

5.3 8-bromo-1-azabicyclo[5.2.0]nonan-9-one (**5c**) 19

5.4 tert-butyl 7-bromo-8-oxo-1,4-diazabicyclo[4.2.0]octane-4-carboxylate (**5d**) 20

5.5 7-bromo-4-oxa-1-azabicyclo[4.2.0]octan-8-one (**5e**) 20

5.6 7-bromo-4-thia-1-azabicyclo[4.2.0]octan-8-one 4,4-dioxide (**5f**) 20

5.7 Synthesis and thermolysis of α-chloro- and α-iodo-**4d** 21

5.8 Dirhodium(II) catalysis results 21

6 Additional procedures 22

6.1 1,1,3,3-tetramethylguanidine p-toluenesulfinate 22

References 23

NMR Spectra **25**
6.2 N-bromophthalimide . . . 25
6.3 N,N'-ditosylhydrazine . . . 26
6.4 2-bromo-1-(pyrrolidin-1-yl)ethanone **(2a)** . . . 28
6.5 2-bromo-1-(piperidin-1-yl)ethanone **(2b)** . . . 30
6.6 1-(azepan-1-yl)-2-bromoethanone **(2c)** . . . 32
6.7 tert-butyl 4-(2-bromoacetyl)piperazine-1-carboxylate **(2d)** . . . 35
6.8 2-bromo-1-morpholinoethanone **(2e)** . . . 37
6.9 2-bromo-1-(1,1-dioxidothiomorpholino)ethanone **(2f)** . . . 39
6.10 2-diazo-1-(pyrrolidin-1-yl)ethanone **(3a)** . . . 41
6.11 2-diazo-1-(piperidin-1-yl)ethanone **(3b)** . . . 43
6.12 1-(piperidin-1-yl)-2-tosylethanone . . . 46
6.13 1-(azepan-1-yl)-2-diazoethanone **(3c)** . . . 48
6.14 tert-butyl 4-(2-diazoacetyl)piperazine-1-carboxylate **(3d)** . . . 51
6.15 2-diazo-1-morpholinoethanone **(3e)** . . . 53
6.16 2-diazo-1-(1,1-dioxido-4-thiomorpholinyl)ethanone **(3f)** . . . 56
6.17 Crude 6-bromo-1-azabicyclo[3.2.0]heptan-7-one **(5a)** . . . 58
6.18 Crude 7-bromo-1-azabicyclo[4.2.0]octan-8-one **(5b)** . . . 59
6.19 Crude 7-bromo-1-azabicyclo[4.2.0]octan-8-one **(5b)** without int. standard . . . 60
6.20 Crude 8-bromo-1-azabicyclo[5.2.0]nonan-9-one **(5c)** . . . 61
6.21 exo-8-bromo-1-azabicyclo[5.2.0]nonan-9-one (exo-**5c)** . . . 62
6.22 endo-8-bromo-1-azabicyclo[5.2.0]nonan-9-one (endo-**5c)** . . . 64
6.23 Crude tert-butyl 7-bromo-8-oxo-1,4-diazabicyclo[4.2.0]octane-4-carboxylate **(5d)** . . . 66
6.24 Crude 7-bromo-4-oxa-1-azabicyclo[4.2.0]octan-8-one **(5e)** . . . 67
6.25 Crude 7-bromo-4-thia-1-azabicyclo[4.2.0]octan-8-one 4,4-dioxide **(5f)** . . . 68
6.26 1,1,3,3-tetramethylguanidine sulfinate (and sulfonate) . . . 69
6.27 2,2-dibromo-1-(piperidin-1-yl)ethanone . . . 70

General introduction

All commercially available reagents and solvents were used as received except in the case of *N*-bromosuccinimide (NBS), which was recrystallized from water (see details below). The CH_2Cl_2 used in the acylation of the amines and in the thermolysis reactions was obtained from an MBraun MB SPS-800 solvent purification system and is dry, but more importantly free of alcoholic stabilizers. Alcohol-stabilized CH_2Cl_2 does not completely inhibit the reactions in question, but traces of ethyl esters (*O*-acylation) and ethyl ethers (carbene O-H insertion product) have been been observed spectroscopically when running the reactions in alcohol-stabilized CH_2Cl_2. The prepared/recrystallized *N*-bromoimides and diazoamides were stored in a freezer at -20 °C to ensure their integrity and were left in ambient temperature for about 5 minutes before exposing them to air in order to minimize weighing errors caused by condensed water.

Except for the above stated routine, no special precautions were taken to exclude air or airborne moisture at any point during the syntheses described in this work, except in the acylation reactions where it was clearly detrimental. Inert atmosphere (Ar or N_2) is used only where stated specifically (acylations and catalyzed reactions). Stirring refers to magnetic stirring in all the procedures. Heating was performed with fluoropolymer-coated all-metal heating mantles.

Chromatography was performed on silica gel (Merck 60, 40-63 μm) and thin layer chromatography (TLC) on aluminium sheets coated with silica gel (Fluka 6607780, 20 mm, fluorescent coating for 254 nm) or neutral alumina (Macherey Nagel Alugram Alox N, 0.20 mm, unactivated, no fluorescent coating). The plates were visualized with $KMnO_4$, K_2CO_3 and NaOH in water for the α-bromo amides (alumina) and UV-light and/or a solution of *p*-anisaldehyde, concentrated H_2SO_4 and glacial CH_3CO_2H in 96% EtOH for the α-diazoacetamides (silica gel), followed by heating with a heat gun for both. The β-lactams (alumina) were visualized with a solution of ninhydrin and pyridine in absolute EtOH followed by heating or with a solution containing $AgNO_3$, H_2O, 2-phenoxyethanol, acetone and H_2O_2 (30 %, aq) followed by exposing the plate to UV-light for about 10 minutes (white spots on beige background).

Nuclear magnetic resonance (NMR) spectroscopy was performed on Bruker DXP300/DXP200 spectrometers operating at 300/200 MHz for ^{1}H and 75/50 MHz for ^{13}C. The chemical shifts are reported in parts per million (δ) relative to the residual solvent signal in general accordance with the literature[1]: δ-^{1}H/δ-^{13}C (Solvent); 7.26/77.00 ($CDCl_3$), 5.32/53.80 (CD_2Cl_2), 2.50/39.52 (DMSO$-d_6$) and 1.94/118.26 (CD_3CN). Chemical shifts are given in the section for physical data for each compound. Additionally, the spectra for the reported compounds are included in the appendix where their signals are tentatively assigned to numbered structures appearing on each spectrum, based on the information gained from the performed NMR experiments. The DEPT-135 spectra were referenced using the ^{13}C-NMR, as noted in the spectra. The NMR data were processed with ACD/Labs NMR Processor Academic Edition.

Mass spectrometry experiments were carried out with a Fisons VG ProSpec for electron impact ionisation (EI) and a Micromass Q-TOF 2 or a Bruker Apex FTMS (FT-ICR, 4.7 T) for electrospray ionisation (ESI). The data from the three instruments are marked (EI), (ESI, Q-TOF) and (ESI, FT-ICR), respectively. The solvent used and other essential details are noted in each case. **NOTE**: Diazo compounds are prone to dinitrogen extrusion, a process that can be effected by means of both heat and acidic media. While the obtention of molecular ions using electron impact ionisation (EI) was straightforward, like others,[2a] we did not observe

molecular ions using electrospray ionisation (ESI) with added HCOOH. The ESI-data for the diazoacetamides could indicate, however, that the observed radical cations are produced upon extrusion of dinitrogen and the inclusion of a molecule of solvent (tentatively assigned). One can speculate if this owes to an interaction of the carbene produced upon nitrogen extrusion with a molecule of solvent.[3] Such a reproducible *m/z*, not corresponding to the molecular ion, has also been noted by others in ESI experiments with EDA.[2a] Interestingly, when running the ESI without added HCOOH, simple sodium adducts of the molecular ions was observed, except in the case of **3f**.

Melting points were measured using a Stuart SMP10 melting point apparatus (Barloworld Scientific Ltd., UK) and are uncorrected.

Yield measurement using an internal standard: The reactions were left to expire overnight, before evaporating the solvent under reduced pressure at or below 23 °C. 0.5 molar equivalents of 2-naphthaldehyde was then added and the exact weight noted, before dissolving the entire crude material in $CDCl_3$. The solution was transferred to an NMR-tube and a ^{1}H-NMR spectrum recorded. The integral of the aldehyde proton of the internal standard was calculated based on its molar fraction in relation to the starting material. Thus, the yields can be read out from the integral of the signal from the β--lactam α--proton in the NMR spectra in the appendix.

1 *N*-bromoimides

1.1 *N*-bromophthalimide

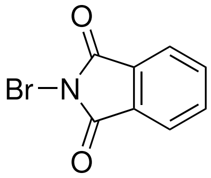

N-bromophthalimide was prepared from phthalimide by extending a procedure published by Souza *et. al*[4] for the preparation of *N*-chloro- and *N*-bromosaccharin, in which bromine is generated *in situ* by oxidation of KBr in water. The original procedure is modified by an additional 0.25 eq of Na_2CO_3.

The reaction proceeds noticeably slower than in the case of sodium saccharin (as in the original publication - preparation not shown), possibly due to the fairly low solubility of phthalimide even in basic water. Another consequence of this, was a prolonged and more visible release of bromine. As the consumption of the released bromine is slower than with saccharin, it was necessary to close the reaction vessel with a glass stopper to contain the evolution of bromine gas. The yield was 65 %. The obtained spectroscopic data were in accordance with the published data.[5] The measured melting point was lower, however, possibly owing to the fact that the recrystallization from toluene left a slightly amorphous crystal mass.

1.1.1 Experimental procedure

Phthalimide (7.36 g, 50.0 mmol, 1.0 eq), Na_2CO_3 (3.98 g, 37.6 mmol, 0.75 eq) and KBr (5.95 g, 50.0 mmol, 1.0 eq) were added through a solid addition funnel to a 500 mL round bottom flask followed by H_2O (Type II, 200 mL). The flask was cooled on an ice/water bath for 10 minutes, after which potassium peroxymonosulfate (as its triple salt $2\,KHSO_5 \cdot KHSO_4 \cdot K_2SO_4$, oxone®) (30.8 g, 50.1 mmol, 1.0 eq) suspended in H_2O (Type II, 75 mL) was added in small aliquots, with vigorous stirring (700 rpm), over a period of 10 minutes. The addition of the oxidant resulted in vigorous release of bromine gas, and thus the flask was capped with a glass stopper between additions. The mixture quickly turned into an orange frothing suspension with visible bromine gas above it. The suspension was left to stir for 24 hours, at which point it had become a yellow solution with a white precipitate. Stirring was continued another 24 hours, until the solution

above the precipitate was almost clear. The suspension was filtered on a Büchner-funnel and the filter cake sucked dry for a period of 30 minutes. The white precipitate was dissolved in boiling toluene (150 mL), hot filtered into a beaker and left to cool slowly to ambient temperature, covered with an aluminium foil. Upon reaching ambient temperature, precipitation had begun and the beaker was placed in a refrigerator at 4 °C for a period of 17 hours, before filtering the precipitate on a Büchner-funnel. The filter cake was washed with *n*-pentane (30 mL) to give 6.52 g (57 %) of small white crystals. The mother liquor was concentrated, filtered and the filter cake washed with toluene (5 mL) and *n*-pentane (10 mL) to give 0.91 g (8 %). Both crops were of equal purity by ^{1}H-NMR. The total yield was 65%.

Physical data

- ^{1}H-NMR (200 MHz, $CDCl_3$) δ 7.95 – 7.81 (m, 2H), 7.81 – 7.68 (m, 2H)
- Melting point: 176 – 184 °C ($C_6H_5CH_3$, decomposition)
  (lit. 199 – 200 °C,[5] CH_2Cl_2)

1.2 *N*-bromosuccinimide

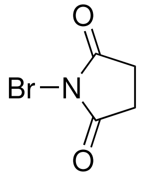

N-bromosuccinimide was purchased from Sigma-Aldrich and recrystallized from boiling water (10 mL g^{-1}) to give thin transparent plates in accordance with the literature (purification method "*Aa*").[6]

2 *N,N'*-ditosylhydrazine

Synthesis route and comments

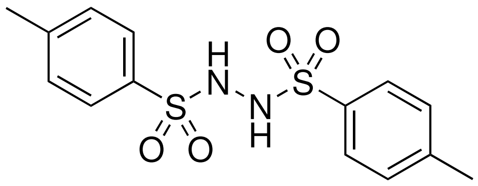

N,N'-ditosylhydrazine was synthesized in 92 % yield in one step by the reaction of tosylhydrazine with tosyl chloride in the presence of pyridine, as described by Toma *et al.*,[7] albeit with a slight modification of the work-up procedure. The modified procedure requires less methanol than the original procedure, making the work-up easier and safer to perform on a preparative scale (∼ 100 g), without compromising the purity of the isolated product.

The reaction was performed once following the original procedure, affording a yield comparable to that reported (∼ 15 g, 85 %). The procedure was originally performed on a 50 mmol scale and employed a recrystallization from 400 mL of boiling MeOH as the final purification step.
In the modified work-up procedure, the crude precipitate (see experimental details below) is simply slurried in methanol at reflux, lowering the expenditure of MeOH considerably.

The reaction could thus be performed at various scales with a low methanol expenditure:

Scale (mmol)	MeOH volume (mL)	Yield (g)	Yield (%)
100	400	31	92
192	500	61	93
300	800	95	93
350	900	109	92

The ^{1}H-NMR and ^{13}C-NMR spectra of the obtained products were identical to those of the product obtained using the original procedure, and are in accordance with the published spectroscopic data.[7] An X-ray structure can also be found in the literature.[8]

Representative experimental procedure

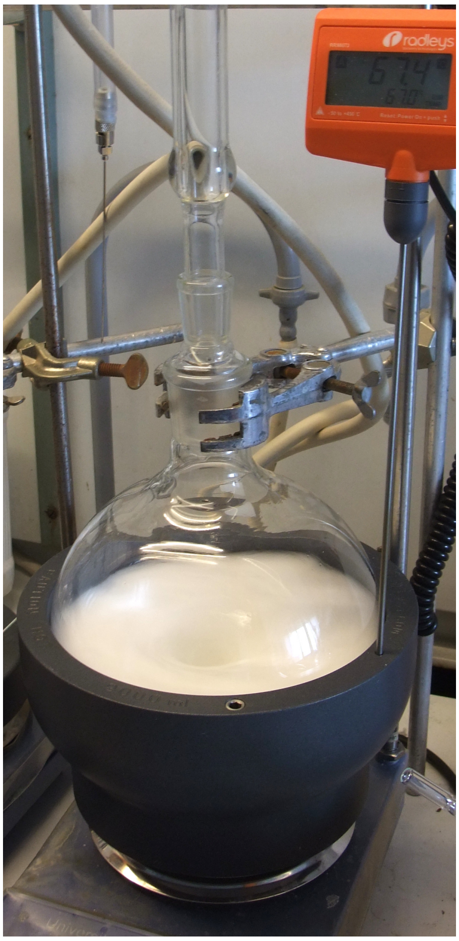

Tosyl chloride (100.0 g, 524.5 mmol, 1.5 eq) and tosylhydrazide (65.12 g, 349.7 mmol, 1.0 eq) were added to a 1000 mL round-bottom flask through a solid addition funnel, and dissolved/suspended in anhydrous CH_2Cl_2 (250 mL), giving a concentration of approximately 1 M based on the limiting reactant. An oval stir bar (3.5 × 1.5 cm) was added and the suspension stirred (700 rpm) while cooling on an ice/water bath, until an internal temperature of 3 °C was reached. Addition of a solution of pyridine (41.49 g, 524.5 mmol, 1.5 eq) in anhydrous CH_2Cl_2 (80 mL) over a period of 10 minutes, in such a way that the internal temperature did not rise above 20 °C. This gave a transparent yellow solution which soon deposited a white precipitate, upon which stirring was increased to 1000 rpm, and continued for a period of 3 hours. Et_2O (200 mL) was added and the suspension solidified. The heavy precipitation was broken up with a spatula and the suspension was transferred to a 1500 mL beaker, where it was stirred with a triangular stir bar (5 × 1 × 1 cm). H_2O (Type II, 200 mL) and Et_2O (200 mL) was added in the given order. Stirring was continued for 10 minutes, before filtering the precipitate on a 13 cm Büchner-funnel, where it was left to air dry with suction for a period of 5 minutes.
The solid was transferred directly to a 2000 mL round bottom flask and suspended in MeOH (900 mL). The flask was equipped with a reflux-condenser and an oval stir bar (5 × 2 cm) before heating the suspension to reflux (mantle temperature 67 °C) and stirring for a period of 3 hours.

The suspension, which at this point would separate to slightly yellow solution above a bright white precipitate, was allowed to cool before filtering on a 13 cm Büchner-funnel. The filter cake was washed with MeOH (300 mL) and Et_2O (300 mL). The filtrate was then concentrated on a rotary evaporator (bath temperature $\sim$ 45 °C) to a volume of approximately 150 mL, before cooling the flask under running tap water. The precipitate was filtered on a Büchner-funnel, washed with MeOH (200 mL) and Et_2O (200 mL) giving a second crop. The first crop was a voluminous white powder, while the second more crystalline crop consisted of short white needles. Both crops were dried in air in a fume hood over a period of 48 hours before determining the yield. Crop I: 97.60 g (81 %), crop II: 12.34 g (10 %). The total yield was 109.94 g (92 %). Both crops were of equal purity by NMR analysis and were subsequently mixed and stored in a screw-capped PTFE jar at ambient temperature.

Physical data

- ^{1}H-NMR (300 MHz, DMSO$-$d$_6$) δ 2.40 (s, 6H), 7.37 – 7.40 (m, 4H), 7.64 – 7.67 (m, 4H), 9.59 (s, 2H)
- ^{13}C-NMR (75 MHz, DMSO$-$d$_6$) δ 21.0, 127.8, 129.4, 135.5, 143.4
- Melting point: 212 – 215 °C (decomposition)

3 α-bromoacetamides

Synthesis route and comments

The α-bromoacetamides were synthesized by the dropwise addition of the amine in CH_2Cl_2 to bromoacetyl bromide and tribasic potassium phosphate in dry, alcohol-free CH_2Cl_2. The procedure was based on a recent publication where the authors investigated the effect of different bases on the formation of amides from amines and acid chlorides.[9] The obtained α-bromoacetamides were sufficiently pure after work-up to be used in the next step without further purification.

Representative experimental procedure

A 250 mL round bottom flask was charged, in the given order, with bromoacetyl bromide (30 mmol, 1.5 eq), anhydrous CH_2Cl_2 (20 mL) and K_3PO_4 (50 mmol, 2.5 eq). An oval stir bar (3.5 × 1.5 cm) was added and the flask was fitted with a 100 mL addition funnel, containing the amine (20 mmol, 1 eq) in CH_2Cl_2 (30 mL). The flask was cooled on an ice/water bath, while purging the system with argon for a period of 5 minutes. Stirring (500 rpm) was commenced and the amine solution added dropwise over a period of 30 minutes, after which the addition funnel was washed with CH_2Cl_2 (5 mL). Stirring was then continued for a period of 3 hours, before quenching the excess acid bromide with aqueous HCl (0.5 M, 30 mL). After stirring another 5 minutes, H_2O (10 mL) and brine (10 mL) were added and the now transparent two phases transferred to a separatory funnel and the organic phase drained off. The aqueous phase was extracted with CH_2Cl_2 (1 × 20 mL), before washing the joint organic phases with a mixture of aqueous $KHCO_3$ (10 %wt, 20 mL) and brine (10 mL), and then with brine (20 mL). Drying over $MgSO_4$ and evaporating under reduced pressure at 30 °C bath temperature, afforded the α-bromoacetamides. The crude material was stored at −20 °C before submission to the next step.

3.1 2-bromo-1-(pyrrolidin-1-yl)ethanone (2a)

Yield: 3.466 g (90 %) from 20.01 mmol of pyrrolidine.
NMR spectra can be found on page 28.

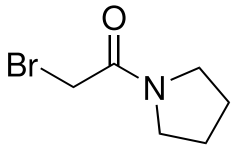

Physical data

- ^{1}H-NMR (300 MHz, $CDCl_3$) δ 3.77 (s, 2H), 3.51 – 3.42 (m, 4H), 2.03 – 1.80 (m, 4H)
- ^{13}C-NMR (75 MHz, $CDCl_3$) δ 164.9, 46.9, 46.3, 27.3, 26.0, 24.2
- MS (EI) *m/z* (% rel. int.) 193/191 (M^+, 16/16), 121/123 (4/4), 112 (100), 98 (81), 93/95 (6/6), 70 (48)
- HRMS (EI) Calcd. for C_6H_{10} ^{79}BrNO [M^+]: 190.9946; found 190.9950 (−2.2 ppm)
- TLC (Neutral Al_2O_3, CH_2Cl_2) R_f: 0.35

3.2 2-bromo-1-(piperidin-1-yl)ethanone (2b)

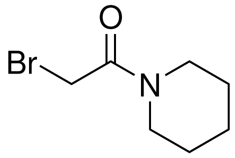

Yield: 7.750 g (94 %) from 40.01 mmol of piperidine.
NMR spectra can be found on page 30.

Physical data

- ^{1}H-NMR (300 MHz, $CDCl_3$) δ 3.84 (s, 2H), 3.57 – 3.49 (m, 2H), 3.46 – 3.36 (m, 2H), 1.69 – 1.59 (m, 4H), 1.59 – 1.48 (m, 2H)
- ^{13}C-NMR (75 MHz, $CDCl_3$) δ 165.0, 47.8, 43.2, 26.1, 26.1, 25.3, 24.2
- MS (EI) *m/z* (% rel. int.) 207/205 (M^+, 4/4), 126 (100), 123/121 (3/4), 112 (20), 95/93 (2/2), 84 (18)
- MS (ESI, Q-TOF) (MeOH) *m/z* 230.0/228.0 $[M + Na]^+$, 208.0/206.0 $[M + H]^+$
- HRMS (EI) Calcd. for C_7H_{12} ^{79}BrNO) [M^+]: 205.0102; found 205.0104 (-1.0 ppm)
- TLC (Neutral Al_2O_3, CH_2Cl_2) R_f: 0.54

3.3 1-(azepan-1-yl)-2-bromoethanone (2c)

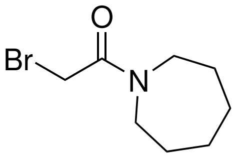

Yield: 4.151 g (94 %) from 20.01 mmol of azepane.
NMR spectra can be found on page 32.

Physical data

- ^{1}H-NMR (300 MHz, $CDCl_3$) δ 3.84 (s, 2H), 3.54 – 3.42 (m, 4H), 1.82 – 1.62 (m, 4H), 1.62 – 1.47 (m, 4H)
- ^{13}C-NMR (75 MHz, $CDCl_3$) δ 166.3, 48.5, 46.3, 28.9, 27.2, 27.1, 26.4, 26.4
- MS (EI) *m/z* (% rel. int.) 221/219 (M^+, 1/1), 140 (100), 126 (7), 123/121 (2/3), 98(8), 95/93 (2/2)
- HRMS (EI) Calcd. for C_8H_{14} ^{79}BrNO) [M^+]: 219.0259; found 219.0257 (0.6 ppm)
- TLC (Neutral Al_2O_3, CH_2Cl_2) R_f: 0.45

3.4 *tert*-butyl 4-(2-bromoacetyl)piperazine-1-carboxylate (2d)

Yield: 5.885 g (95 %) from 20.00 mmol of *tert*-butyl piperazine-1-carboxylate.
NMR spectra can be found on page 35.

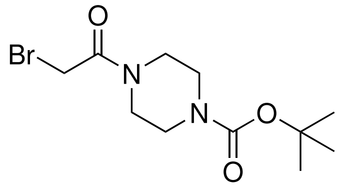

Physical data

- ^{1}H-NMR (300 MHz, $CDCl_3$) δ 3.85 (s, 2H), 3.62 – 3.38 (m, 8H), 1.45 (s, 9H)
- ^{13}C-NMR (75 MHz, $CDCl_3$) δ 165.4, 154.4, 80.4, 46.5 (2C), 41.9 (2C), 28.3 (3C), 25.6
- MS (EI) *m/z* (% rel. int.) 308/306 (M^+, 4/4), 252/250 (9/9), 235/233 (9/9), 207/205 (2/1), 171 (22), 170 (37), 127 (16), 123/121 (3/3), 113 (14), 95/93 (1/2), (85 (35), 57 (100)
- HRMS (EI) Calcd. for $C_{11}H_{19}$ $^{79}BrN_2O_3$) [M^+]: 306.0579; found 306.0587 (−2.7 ppm)
- TLC (Neutral Al_2O_3, CH_2Cl_2) R_f: 0.14

3.5 2-bromo-1-morpholinoethanone (2e)

Yield: 3.737 g (89 %) from 20.00 mmol of morpholine.
NMR spectra can be found on page 37.

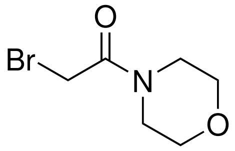

Physical data

- ^{1}H-NMR (300 MHz, $CDCl_3$) δ 3.83 (s, 2H), 3.75 – 3.64 (m, 4H), 3.64 – 3.56 (m, 2H), 3.54 – 3.47 (m, 2H)
- ^{13}C-NMR (75 MHz, $CDCl_3$) δ 165.3, 66.5, 66.3, 47.1, 42.3, 25.4
- MS (EI) *m/z* (% rel. int.) 209/207 (M^+, 21/21), 194/192 (14/15), 128 (100), 123/121 (14/14), 114 (16), 95/93 (5/5), 86 (79)
- HRMS (EI) Calcd. for C_6H_{10} $^{79}BrNO_2$) [M^+]: 206.9895; found 206.9888 (3.5 ppm)
- TLC (Neutral Al_2O_3, CH_2Cl_2) R_f: 0.37

3.6 2-bromo-1-(1,1-dioxidothiomorpholino)ethanone (2f)

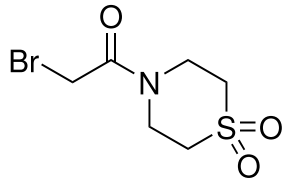

The solubilities of both the amine thiomorpholine-1,1-dioxide and the obtained amide are relatively low in CH_2Cl_2. As such, the volume of CH_2Cl_2 in which the amine was initially dissolved was increased to 7.40 mmol/30 mL. Additionally, on the same scale, the reaction mixture was diluted with CH_2Cl_2 (100 mL) after quenching with HCl (aq), before separating the phases. A longer reaction time of 24 hours was also needed to achieve full conversion of the amine.

Yield: 1.733 g (91 %) from 7.40 mmol of thiomorpholine-1,1-dioxide.
NMR spectra can be found on page 39.

Physical data

- ^{1}H-NMR (300 MHz, CD_3CN) δ 4.06 (s, 2H), 4.01 – 3.86 (m, 4H), 3.20 – 2.96 (m, 4H)
- ^{13}C-NMR (75 MHz, CD_3CN) δ 163.2, 52.3, 52.1, 45.7, 41.6, 27.8
- MS (EI) *m/z* (% rel. int.) 257/255 (M^+, 1/1), 193/191 (1/1), 192/190 (2/2), 176 (41), 162 (9), 134 (45), 123/121 (14/14), 112 (100), 95/93 (8/9), 70 (44)
- HRMS (EI) Calcd. for C_6H_{10} $^{79}BrNO_3S$) [M^+]: 254.9565; found 254.9565 (-0.2 ppm)
- TLC (Neutral Al_2O_3, CH_2Cl_2) R_f: 0.29

4 α-diazoacetamides

4.0.1 Synthesis route and comments

In the procedure reported by Toma et al. the amidine base 1,8-Diazabicyclo-[5.4.0]-undec-7-ene (DBU) was used to effect the nucleophilic attack of the *N,N'*-ditosylhydrazine anion on the α-carbon of α-bromoacetic esters, as well as the subsequent decomposition of the resulting hydrazide/hydrazone intermediate to yield an α-diazo group.[7] The lower cost and similar base strength[10] of 1,1,3,3-Tetramethylguanidine (TMG), as well as its properties as a nucleophilic catalyst,[11] made it an interesting alternative to DBU in this transformation. Furthermore, in the original procedure, the crude reaction mixture was quenched with $NaHCO_3$(aq) and then extracted with Et_2O. In our experience it was problematic to perform a clean aqueous extraction in the presence of the at least 2 molar equivalents of $DBU{-}HO_2S\text{-}C_6H_4CH_3$ produced during the reaction. This situation manifested itself more gravely on a $>$ 5 mmol scale. The problem could lie in the intermediate solubility of the $DBU{-}HO_2S\text{-}C_6H_4CH_3$ complex between the aqueous phase and the Et_2O phase. What further complicated the matter of an aqueous extraction procedure, was the aqueous solubility of some of the obtained α-diazoacetamides.

The use of TMG instead of DBU resulted in the partial precipitation of the $TMG \cdot HO_2S\text{-}C_6H_4CH_3$ salt from THF during the reaction. Thus, in the cases where the diazoamide was soluble in Et_2O (products **3a - e**), the reaction solvent (THF) could be destilled off under reduced pressure and the diazoamide extracted from the resulting solid with Et_2O (the $TMG \cdot HO_2S\text{-}C_6H_4CH_3$ salt is poorly soluble in Et_2O). A final clearing of the Et_2O-phase by slurrying with silica gel and filtering, yielded the diazoamides with decent purities, without the need for an aqueous work-up. As noted by Toma et al., we also observed the formation of the corresponding α-tosylacetamides, resulting from the nucleophilic attack of the produced *p*-toluenesulfinic acid on the substrate α-bromoacetamides. This side-reaction can however be minimized by maintaining the reaction temperature below 0 °C (see procedure below).

In the case where the diazoamide was not soluble in Et_2O (product **3f**), a THF/Et_2O (2:1) mixture could be used to extract the diazoamide with adequate results (see the experimental procedure for product **3f** below).

All the obtained diazoamides were subsequently chromatographed on silica gel to ensure that they were free of contaminations (e.g. their corresponding α-tosylacetamides) that could interfere in the investigation of their halogenation and subsequent reactions. The details pertaining to the chromatography for each compound are given below.

4.0.2 Representative experimental procedure (products 3a - e)

To a 250 mL round bottom flask containing the α-bromoacetamide (20 mmol, 1 eq), were added through a solid addition funnel; *N,N′*-ditosylhydrazine (40 mmol, 2 eq), THF (35 mL) and an oval stir bar (3.5 × 1.5 cm). The suspension was cooled on an acetone/CO_2 (s) bath with stirring (400 rpm) until reaching an internal temperature of −10 °C, upon which a solution of 1,1,3,3-tetramethylguanidine (100 mmol, 5 eq) in THF (10 mL) was added in aliquots using a pasteur pipette, while maintaining the internal temperature below 0 °C by the addition of CO_2 (s). The resulting yellow solution was removed from the cooling bath and allowed to warm to ambient temperature over a period of 30 minutes, during which time a white precipitate is deposited. This suspension was stirred for a period of 90 minutes, before removing the THF under reduced pressure at or below 23 °C. The resulting solid was treated with anhydrous Et_2O (150 mL) and vigorously stirred for 10 minutes, before being filtered on a Büchner-funnel. The filter cake was washed with anhydrous Et_2O (2 - 5 × 50 mL), until leaving an almost white filter cake. Approximately 25 g (1 large tablespoon) of silica gel was added to the filtrate and the suspension stirred for 5 minutes, followed by vacuum filtration through a pad of silica gel (∼ 25 g, column ⌀ 3.5 cm). The column was washed with Et_2O (50 mL) and the Et_2O evaporated under reduced pressure at or below 23 °C. The thus obtained crude diazoacetamides were then subjected to flash chromatography on silica gel eluting increasing amounts of Et_2O in Pet.Ether. The products were stored at −20 °C before submission to the next step.

4.1 2-diazo-1-(pyrrolidin-1-yl)ethanone (3a)

Yield: 1.706 g (67 %) as a yellow oil starting from 18.05 mmol **2a**. The compound is a solid at −20 °C (picture), and is previously reported in the literature.[12, 13]
NMR spectra can be found on page 41.

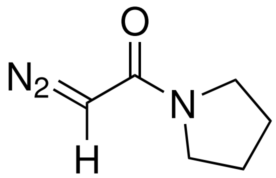

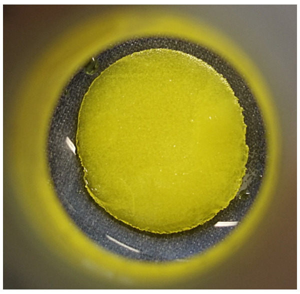

Physical data

- ^{1}H-NMR (300 MHz, CD_2Cl_2) δ 4.92 (s, 1H), 3.54 – 2.98 (m, 4H), 2.06 – 1.64 (m, 4H)
- ^{13}C-NMR (75 MHz, CD_2Cl_2) δ 163.8, 46.4, 46.2 (2C), 26.1, 24.8
- MS (ESI, FT-ICR) (MeCN, no HCOOH) *m/z* 162.0 $[M + Na]^+$
- TLC (SiO_2, Et_2O) R_f: 0.13

4.2 2-diazo-1-(piperidin-1-yl)ethanone (3b)

Yield: 3.008 g (52 %) as a yellow oil starting from 37.61 mmol **2b**. The compound is a solid at −20 °C (picture), and is previously reported in several publications.[14–21]
NMR spectra can be found on page 43.

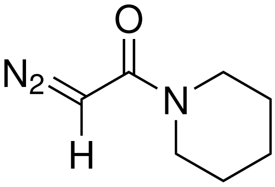

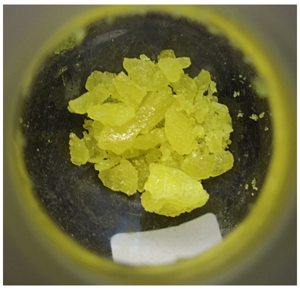

Physical data

- ^{1}H-NMR (300 MHz, CD_2Cl_2) δ 5.16 (s, 1H), 3.30 (bs, 4H), 1.66 – 1.55 (m, 2H), 1.55 – 1.42 (m, 4H)
- ^{13}C-NMR (75 MHz, CD_2Cl_2) δ 164.3, 46.1, 45.2 (2C), 26.2, 24.8 (2C)
- MS (EI) *m/z* (% rel. int.) 154 (5), 153 (M^+, 25), 125 (19), 112 (11), 96 (45), 84 (38), 69 (53), 55 (71), 42 (100), 41 (79)
- MS (ESI, Q-TOF) (MeCN) *m/z* 167.1 $[M + H - N_2 + MeCN]^+$
- MS (ESI, Q-TOF) (MeOH) *m/z* 180.1 $[M + Na - N_2 + MeOH]^+$, 158.1 $[M + H - N_2 + MeOH]^+$
- MS (ESI, FT-ICR) (MeCN, no HCOOH) *m/z* 176.1 $[M + Na]^+$
- HRMS (EI) Calcd. for $C_7H_{11}N_3O$) $[M^+]$: 153.0902; found 153.0891 (7.2 ppm)
- TLC (SiO_2, Et_2O) R_f: 0.28

1-(piperidin-1-yl)-2-tosylethanone — a commonly observed by-product.[7] See NMR-spectra on page 46:

- MS (EI) *m/z* (% rel. int.) 282 (M^++1, 1), 217 (26), 155 (6), 127 (14), 126 (100), 112 (42), 110 (30), 97 (29), 91 (43), 84 (34)
- MS (ESI, Q-TOF) (MeCN) *m/z* 282.2 $[M + H]^+$, 585.3 $[_2M + Na]^+$

4.3 1-(azepan-1-yl)-2-diazoethanone (3c)

Yield: 2.217 g (66 %) as a soft yellow solid starting from 18.85 mmol **2c**.
The compound is previously reported as a reagent,[21] however no details on its synthesis were disclosed.
NMR spectra can be found on page 48.

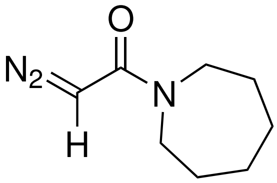

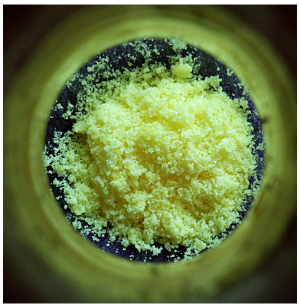

Physical data

- ^{1}H-NMR (300 MHz, CD_2Cl_2) δ 5.07 (s, 1H), 3.68 – 3.00 (m, 4H), 1.79 – 1.61 (m, 4H), 1.61 – 1.43 (m, 4H)
- ^{13}C-NMR (75 MHz, CD_2Cl_2) δ 165.2, 47.8, 46.2 (2C), 28.8 (2C), 27.5, 27.3

- MS (EI) *m/z* (% rel. int.) 168 (7), 167 (M^+, 16), 139 (7), 138 (15), 126 (8), 111 (9), 110 (21), 96 (61), 69 (64), 55 (65), 41 (100)
- MS (ESI, FT-ICR) (MeCN, no HCOOH) *m/z* 190.1 $[M + Na]^+$
- HRMS (EI) Calcd. for $C_8H_{13}N_3O$ $[M^+]$: 167.1059; found 167.1060 (−0.6 ppm)
- TLC (SiO_2, Et_2O) R_f: 0.39
- Melting point: 48 – 50 °C

Dimerization was observed in the mass spectrometer (EI) as has been noted by others.[2b] The dimer radical cation had the following characteristics:

- MS (EI) *m/z* (% rel. int.) 279 (5), 278 (M^+, 4), 181 (12), 180 (12), 152 (8), 149 (17), 110 (13), 98 (100)
- HRMS (EI) Calcd. for $C_{16}H_{26}N_2O_2$ $[M^+]$: 278.1994; found 278.1984 (3.8 ppm)

4.4 *tert*-butyl 4-(2-diazoacetyl)piperazine-1-carboxylate (3d)

Yield: 2.938 g (60 %) as a bright yellow solid starting from 19.16 mmol **2d**.
The compound is to the best of our knowledge previously unreported as a reagent. Our group recently reported the x-ray structure of **3e**.[22]
NMR spectra can be found on page 51.

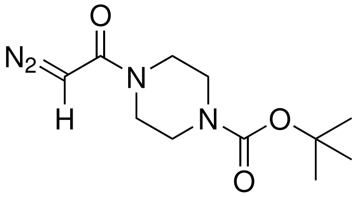

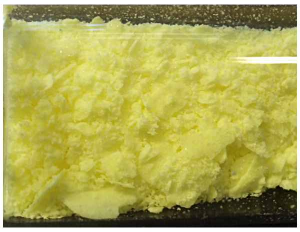

Physical data

- ^{1}H-NMR (300 MHz, CD_2Cl_2) δ 5.13 (s, 1H), 3.47 – 3.21 (m, 8H), 1.42 (s, 9H)
- ^{13}C-NMR (75 MHz, CD_2Cl_2) δ 165.0, 154.6, 80.1, 46.5, 43.9 (br, 4C), 28.4
- MS (EI) *m/z* (% rel. int.) 255 (1), 254 (M^+, 10), 198 (11), 181 (6), 170 (64), 128 (8), 125 (6), 97 (10), 85 (31), 69 (20), 57 (100), 41 (32)
- MS (ESI, Q-TOF) (MeCN) *m/z* 308.2 $[M{-}N_2 + 2\,MeCN]^+$, 268.2 $[M + H{-}N_2 + MeCN]^+$, 212.1 $[M + H{-}N_2{-}56\,(\textit{iso}{-}\text{butene}) + MeCN]^+$
- MS (ESI, Q-TOF) (MeOH) *m/z* 539.3 $[_2M + Na{-}2\,N_2 + 2\,MeOH]^+$, 281.1 $[M + Na{-}N_2 + MeOH]^+$, 203.1 $[M + H{-}N_2{-}56\,(\textit{iso}{-}\text{butene}) + MeOH]^+$
- MS (ESI, Q-TOF) (MeCN, no HCOOH) *m/z* 277.1 $[M + Na]^+$
- HRMS (EI) Calcd. for $C_{11}H_{18}N_4O_3$) $[M^+]$: 253.1379; found 254.1374 (2.1 ppm)
- TLC (SiO_2, Et_2O) R_f: 0.20
- Melting point: 108 – 111 °C

4.5 2-diazo-1-morpholinoethanone (3e)

Yield: 1.677 g (54 %) as a yellow oil starting from 17.97 mmol **2e**.
The compound is previously reported in several publications.[17,20,23]
NMR spectra can be found on page 53.

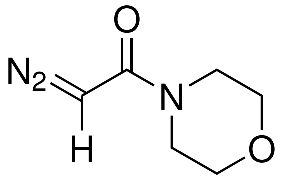

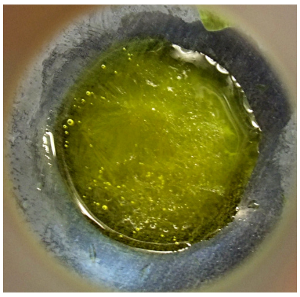

Physical data

- ^{1}H-NMR (300 MHz, CD_2Cl_2) δ 5.05 (s, 1H), 3.68 – 3.56 (m, 4H), 3.37 – 3.23 (m, 4H)
- ^{13}C-NMR (75 MHz, CD_2Cl_2) δ 165.1, 66.9 (2C), 46.3, 44.4 (2C)
- MS (EI) *m/z* (% rel. int.) 156 (10), 155 (M^+, 74), 140 (13), 127(8), 114 (6), 98 (22), 86 (25), 69 (65), 56 (81), 41 (100)
- MS (ESI, FT-ICR) (MeCN, no HCOOH) *m/z* 178.0 $[M + Na]^+$
- HRMS (EI) Calcd. for $C_6H_9N_3O_2$) [M^+]: 155.0695; found 155.0693 (1.1 ppm)
- TLC (SiO_2, Et_2O) R_f: 0.15

4.6 2-diazo-1-(1,1-dioxido-4-thiomorpholinyl)ethanone (3f)

Yield: 0.987 g (64 %) as a light yellow solid starting from 7.53 mmol **2f**.
The compound is generally poorly soluble in common solvents, and is to the best of our knowledge previously unreported as a reagent. Our group recently published the x-ray structure of **3f**.[24]
NMR spectra can be found on page 56.

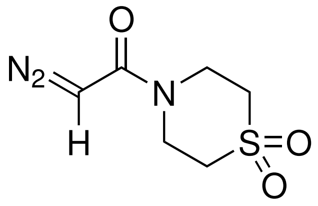

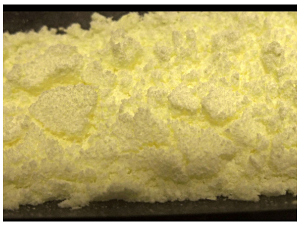

4.6.1 Experimental procedure

To a 250 mL round bottom flask containing 2-bromo-1-(1,1-dioxidothiomorpholino)ethanone (1.928 g, 7.53 mmol, 1 eq), were added through a solid addition funnel *N,N'*-ditosylhydrazine (5.129 g, 15.07 mmol, 2 eq), THF (50 mL) and an oval stir bar (3.5 × 1.5 cm). The suspension was cooled on an ice/water bath with stirring (400 rpm) for a period of 15 minutes, upon which a solution of 1,1,3,3-tetramethylguanidine (4.337 g, 37.65 mmol, 5 eq) in THF (10 mL) was added in bulk. The white suspension quickly became yellow, and was then stirred for a period of 70 minutes, before adding Et_2O (100 mL) causing the disappearance of the yellow colour from the THF/Et_2O phase, as the product was no longer soluble. Removal of some of the Et_2O under reduced pressure, brought the yellow colour back to the liquid phase in the suspension. The mixture was filtered on a Büchner-funnel and the filter cake washed with THF/Et_2O (2:1, 75 mL) and THF (30 mL), leaving an almost white filter cake.

A preliminary purification was performed by adding Celite (~ 60 g, 3 tablespoons) to the filtrate, and evaporating the solvents under reduced pressure at or below 23 °C. The resulting

slightly sticky powder, was suspended in CH_2Cl_2 (15 mL) and loaded on top of 1 cm of sea-sand covering a column of silica gel (8.0 × 3.5 cm) packed in CH_2Cl_2. A yellow band was eluted with MeOH/CH_2Cl_2 (5:95). To this fraction was added silica gel (~ 50 g, 2 large tablespoons) and the solvents evaporated, leaving a pale yellow powder. The powder was loaded on top of a column (5.5 × 3.5 cm) of silica gel packed in CH_2Cl_2, and the product slowly eluted with CH_2Cl_2 and from the point of elution $CHCl_3$. The solvents were evaporated under reduced pressure at or below 23 °C affording 0.987 g (64 %) of a pale yellow powder which was stored at −20 °C.

Physical data

- ^{1}H-NMR (300 MHz, CD_2Cl_2) δ 5.13 (s, 1H), 3.94 – 3.80 (m, 4H), 3.07 – 2.97 (m, 4H)
- ^{13}C-NMR (75 MHz, CD_2Cl_2) δ 165.1, 52.4 (2C), 47.2, 42.8 (2C)
- MS (EI) *m/z* (% rel. int.) 205 (1), 204 (2), 203 (M^+, 19), 175 (2), 162 (5), 134 (21), 118 (6), 111 (5), 83 (100), 70 (30), 69 (94)
- MS (ESI, FT-ICR) (MeCN, no HCOOH) *m/z* 227.1 $[M + Na + H]^+$
- HRMS (EI) Calcd. for $C_6H_9N_3O_3S$) [M^+]: 203.0365; found 203.0366 (−0.7 ppm)
- Melting point: 165 – 176 °C (decomposition)

5 β-lactams

The halogenated α-bromodiazoacetic ethyl esters were reported to be stable for days at 0 °C.[25] The α-halodiazoamides, however, appear less stable. In our experience the 2 – 5 °C temperatures, obtainable with a common ice/water bath, were not sufficiently low to work with the α-halodiazoacetamides with adequate reproducibility. Therefore, all procedures were carried out at −5 °C using an acetone bath in which the temperature was controlled by a cryostat.

In the summaries from the mass spectrometry experiments, the R-group in "α,α'-Br_2−NR_2" refers to the cyclic amide side chain of the respective compound.

Representative experimental procedure

The diazoacetamide (0.5 mmol, 1.0 eq) was weighed out in a 25 mL pear shaped flask, DBU (0.7 mmol, 1.4 eq) in a 10 mL beaker and the *N*-halo compound (0.65 mmol, 1.3 eq) in a weighing funnel. The flask was charged with a small magnetic stir bar (10 × 2 mm) and CH_2Cl_2 (2 mL, ambient temperature), and was subsequently mounted alongside a 250 mL round bottom flask containing CH_2Cl_2 (250 mL) in an acetone bath cooled by a cryostat to −5 °C (measured with an alcohol thermometer at the surface). Stirring (300 RPM) was commenced and after 2 minutes the base in CH_2Cl_2 (2 mL, ambient temperature) was added. After 2 minutes the *N*-halo compound was added in bulk through the funnel and the funnel rinsed with CH_2Cl_2 (1 mL, ambient temperature). The addition effected a colour change from bright yellow to bright red. While the reaction was stirring, a short plug of silica gel (2 × 2 cm) was packed in CH_2Cl_2 (ambient temperature) in a fritted flash chromatography column (15 × 2 cm) and mounted inside a holder which allows for the filling of dry ice around the column (covering ~ 300°, leaving an observation window, see figure 5.1 on the facing page). After stirring for 5 minutes, the thermometer was placed in the 250 mL flask and CO_2(s) was added to the bath until the internal temperature of the CH_2Cl_2 had reached −15 °C. The space between the jacket and the chromatography column was filled with dry ice, and the column equilibrated with CH_2Cl_2 (−15 °C, 20 mL). The reaction mixture was then poured directly onto the column, and the flask rinsed with two pipette volumes of CH_2Cl_2 (−15 °C). The reaction mixture was run into the column using pressurized air (ambient temperature), and the column filled up with CH_2Cl_2

(−15 °C). The column was then run with CH_2Cl_2 (−15 °C, ∼ 200 mL) under air pressure to completely elute a bright red band into a new 250 mL flask in the cooling bath. The eluted solution is bright red/pink in colour with a temperature of approximately −30 °C.

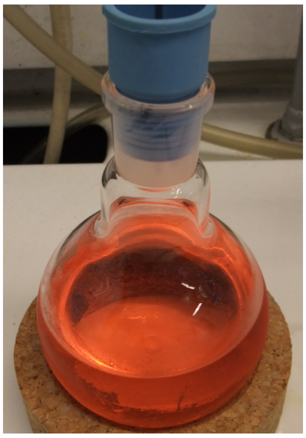

Thermolysis: The flask was placed on the bench without stirring while the temperature rose from −30 °C towards ambient temperature. The solution became clear in approximately 60 minutes. In the chromatographic step, for the more polar analogues, it was necessary to add Et_2O (5 mL) to the last 50 mL of the eluent to completely collect the red band. It should be noted that Et_2O has a much higher elution strength than CH_2Cl_2 under these conditions and fortunately does not seem to interfere with the C-H insertion reaction at this concentration. We have however observed that higher concentrations of Et_2O will make intermolecular reactions (ylide formation, C-H insertion) sufficiently favoured so as to compete with the intramolecular C-H insertions.

Catalyzed reactions: The flask was charged with a stir bar, capped with a septum and maintained in the cooling bath at −15 °C (cryostat) while degassing with argon (5 minutes). The flask was then taken out of the cooling bath and a degassed solution of the catalyst in CH_2Cl_2 (2 mL) was added via syringe under vigorous stirring. The solution was then left to heat towards room temperature.

For an illustration of the CO_2(s)-cooled column used in this procedure, see figure 5.1 below.

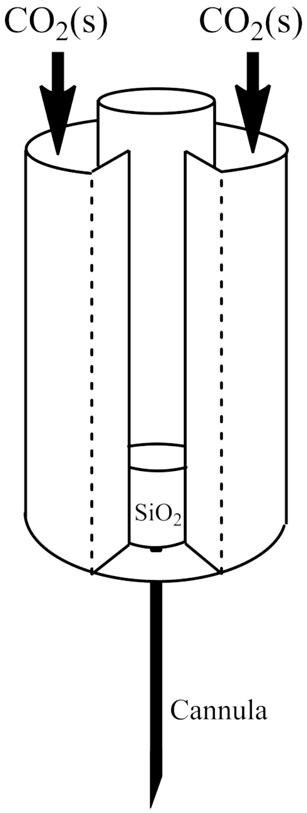

Figure 5.1: An illustration of the column with a compartment for CO_2(s)

5.1 6-bromo-1-azabicyclo[3.2.0]heptan-7-one (5a)

The compound was prepared following the general procedure for thermolysis given above. In $CDCl_3$, the compound decomposes over time as indicated by a ^{1}H-NMR spectrum recorded 48 hours after the first (data not shown). This could owe to the oxidative accumulation of HCl in the $CDCl_3$ resulting in β--lactam hydrolysis, and would be consistent with the reported rapid hydrolysis of β-lactams fused with 5-membered rings.[14]
An NMR spectrum of the crude reaction mixture, added internal standard, can be found on page 58.

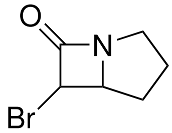

Physical data

Crude reaction mixture from thermolysis:

- Partial ^{1}H-NMR (300 MHz, $CDCl_3$) δ 4.395 (d, J = 1.7 Hz, 1H)
- MS (ESI, FT-ICR) (MeCN) m/z 274/272/270 $[\alpha,\alpha'\text{-}Br_2{-}NR_2 + H]^+$, 210/208 $[M + H_2O + H]^+$ (hydrolysis)

5.2 7-bromo-1-azabicyclo[4.2.0]octan-8-one (5b)

The compound was prepared both following the general procedure for thermolysis and that for catalysis, given above.
NMR spectra of the crude reaction mixtures and crude reactions mixture added internal standard, can be found on page 59.

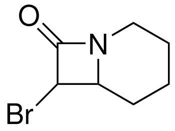

Physical data

Crude reaction mixture from thermolysis, mixture of *endo*/*exo*-isomers:

- ^{1}H-NMR (300 MHz, $CDCl_3$) δ 4.945 (dd, J = 4.43, 1.60 Hz, 1H, *endo*), 4.395 (d, J = 1.13 Hz, 1H, *exo*), 3.805 (dd, J = 13.19 , 4.52 Hz, 1H), 3.50 (ddd, J = 10.69, 4.47, 1.04 Hz, 1H), 2.735 (ddd, J = 13.47, 11.77, 4.33 Hz, 1H), 2.10 (m, 1H), 1.95 – 1.10 (comp, 5H)

Crude reaction mixture from dirhodium(II) catalysis:

- MS (ESI, Q-TOF) (MeOH) m/z 433/431/429 $[C_{17}H_{20}Br_2N_2O_2 + Na]^+$ (formal carbene dimer), 288/286/284 $[\alpha,\alpha'\text{-}Br_2{-}NR_2 + H]^+$, 206/204 $[M + H]^+$

Isolated by preparative TLC:

- MS (EI) m/z (% rel. int.) 205/203 (M^+, 6/6), 134/132 (22/22), 124 (100), 121/119 (7/7), 96 (8), 84 (11), 81 (22), 69 (9)
- HRMS (EI) Calcd. for C_7H_{10} ^{79}BrNO [M^+]: 202.9946; found 202.9938 (3.6 ppm)
- TLC (SiO_2, Et_2O) R_f *exo*: 0.29, *endo*: 0.19

α,α'-Br_2–piperidine acetamide — a common by-product. See ^{1}H-NMR spectrum on page 70:

- ^{1}H-NMR (300 MHz, $CDCl_3$) δ 6.21 (s, $HCBr_2$–NR_2), 6.14 (s, $HCBr_2$–NR_2), 3.75 – 3.50 (m, 4H), 1.80 – 1.50 (m, 6H)
- MS (EI) *m/z* (% rel. int.) 287/285/283 (M^+, 6/13/6), 206/204 (56/57), 175/173/171 (4/9/4), 122/120 (6/6), 112 (100), 84 (21), 69 (69)
- TLC (SiO_2, Et_2O) R_f 0.54

5.3 8-bromo-1-azabicyclo[5.2.0]nonan-9-one (5c)

The compound was prepared following the general procedure for thermolysis given above.
NMR spectra of the crude reaction mixture added internal standard, as well as the *exo*/*endo* isomers isolated by chromatography, can be found on page 61.

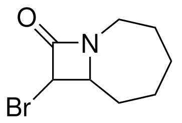

Chromatographic procedure

The diastereomers *exo*-**3e** and *endo*-**3e** were separated on a column of neutral alumina (10 × 2.5 cm ⌀), using Et_2O as the eluent.

Physical data

Isolated by chromatography:

exo-8-bromo-1-azabicyclo[5.2.0]nonan-9-one

- ^{1}H-NMR (300 MHz, $CDCl_3$) δ 4.255 (dd, J = 1.13, 1.13 Hz, 1H), 3.765 (ddd, J = 9.61, 2.83, 1.70 Hz, 1H), 3.45 – 3.23 (m, 2H), 2.15 – 2.05 (m, 1H), 1.95 – 1.70 (m, 3H), 1.60 – 1.25 (m, 4H)
- ^{13}C-NMR (75 MHz, $CDCl_3$) δ 162.5, 64.6, 45.7, 43.4, 33.7, 29.2, 27.2, 26.6

endo-8-bromo-1-azabicyclo[5.2.0]nonan-9-one

- ^{1}H-NMR (300 MHz, $CDCl_3$) δ 4.905 (ddd, J = 4.62, 1.79, 0.75 Hz, 1H), 3.85 (ddd, J = 10.13, 4.66, 2.17 Hz, 1H), 3.40 – 3.25 (m, 2H), 2.00 – 1.75 (m, 5H), 1.60 – 1.20 (m, 5H)
- ^{13}C-NMR (75 MHz, $CDCl_3$) δ 163.2, 57.1, 47.9, 43.5, 33.3, 28.9, 28.1, 26.7

Crude reaction mixture from thermolysis, mixture of *endo*/*exo*-isomers:

- MS (ESI, FT-ICR) (MeCN) *m/z* 220/218 $[M + H]^+$
- TLC (SiO_2, Et_2O) R_f *exo*: 0.49, *endo*: 0.36, α,α'-Br_2–NR_2: 0.65

5.4 *tert*-butyl 7-bromo-8-oxo-1,4-diazabicyclo[4.2.0]octane-4-carboxylate (5d)

The compound was prepared following the general procedure for thermolysis given above.
An NMR spectrum of the crude reaction mixture, added internal standard, can be found on page 66.

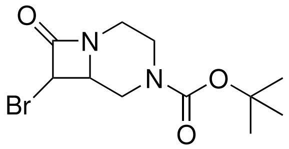

Physical data

Crude reaction mixture from thermolysis:

- Partial ^{1}H-NMR (300 MHz, $CDCl_3$) δ 4.935 (dd, J = 4.52, 1.13 Hz, 1H, *endo*), 4.495 (d, J = 1.32 Hz, 1H, *exo*)
- MS (ESI, FT-ICR) (MeCN) *m/z* 325/323 $[M + H_2O + H]^+$ (hydrolysis), 251/249 $[M-56$ (*iso*$-$butene) $+ H]^+$ (Boc-degradation pathway to carboxylate)

5.5 7-bromo-4-oxa-1-azabicyclo[4.2.0]octan-8-one (5e)

The compound was prepared following the general procedure for thermolysis given above.
An NMR spectrum of the crude reaction mixture, added internal standard, can be found on page 67.

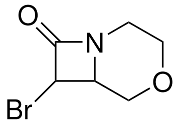

Physical data

Crude reaction mixture from thermolysis:

- Partial ^{1}H-NMR (300 MHz, $CDCl_3$) δ 4.935 (dd, J = 4.52, 1.32 Hz, 1H, *endo*), 4.485 (d, J = 1.32 Hz, 1H, *exo*)
- MS (ESI, FT-ICR) (MeCN) *m/z* 415/413/411 $[C_{12}H_{16}Br_2N_2O_4 + H]^+$ (formal carbene dimer), 208/206 $[M + H]^+$

5.6 7-bromo-4-thia-1-azabicyclo[4.2.0]octan-8-one 4,4-dioxide (5f)

The compound was prepared following the general procedure for thermolysis given above.
An NMR spectrum of the crude reaction mixture, added internal standard, can be found on page 68.

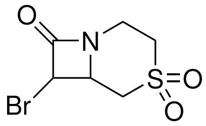

Physical data

Crude reaction mixture from thermolysis:

- Partial ^{1}H-NMR (300 MHz, $CDCl_3$) δ 5.20 (dd, J = 4.71, 1.32 Hz, 1H, *endo*), 4.70 (d, J = 1.51 Hz, 1H, *exo*)
- MS (ESI, FT-ICR) (MeCN) *m/z* 274/272 $[M + H_2O + H]^+$ (hydrolysis)

5.7 Synthesis and thermolysis of α-chloro- and α-iodo-4d

It was desirable to investigate whether the reaction of the α-bromodiazoacetamides could be extended to its chlorine and iodine analogues. The piperazine-derivative **3d** was chosen as the model compound, since the C-H insertion reaction of **Br-4d** gave a moderate to low yield, which provided an opportunity for its chlorine and iodine analogues to expose their differential reactivity. The halogenation reactions were carried out using DBU/NXS (X = Cl, Br, I), due to the availability of the halosuccinimides.

Table 5.1: Yields[a] in % of *exo*/*endo*-*tert*-butyl 7-halo-8-oxo-1,4-diazabicyclo[4.2.0]octane-4-carboxylate **X-5d** (X = Cl, Br, I) obtained from the thermolysis of α-chloro-, α-bromo- and α-iodo-*tert*-butyl 4-(2-diazoacetyl)piperazine-1-carboxylate

Diazo compound	*exo*	*endo*	*exo*/*endo* ratio	Total
Cl-**4d**	10	2	5:1	12
Br-**4d**	34	2	17:1	36
I-**4d**	2[b]	-[bc]	n/a	2

[a] Determined by ^{1}H-NMR (method described above)
[b] The bright red solution turned purple when passed through the short silica gel column used (see experimental procedure), possibly indicating a release of I_2.
[c] Not detected by ^{1}H-NMR

5.8 Dirhodium(II) catalysis results

The results were obtained following the representative experimental procedure for catalyzed reactions given above. NBS was used instead of NBP, due to the higher retention of succinimide vs phthalimide on the short column used to purify the β-diazoacetamide.

Table 5.2: Yields[a] in % of *exo*/*endo*-7-bromo-1-azabicyclo[4.2.0]octan-8-one (**5b**) obtained from the catalytic dediazotization of **4b**.

Catalyst	Nominal catalyst loading (% mol)	*exo*	*endo*	*exo*/*endo* ratio	Total	$\alpha,\alpha' - Br_2$-Amide
$Cu(acac)_2$	3	2	-[b]	n/a	2	2
$Rh_2(tfa)_4$	3	22	6	3.7:1	28	25
$Rh_2(OAc)_2(pc)_2$[26]	1	30	4	7.5:1	34	10
$Rh_2(cap)_4$[27]	3	38	6	6.3:1	44	14

[a] Determined by ^{1}H-NMR (method described above)
[b] Not detected by ^{1}H-NMR

6 Additional procedures

6.1 1,1,3,3-tetramethylguanidine *p*-toluenesulfinate

The precipitation of this salt occurred during the preparations of the α-diazoacetamides as described in their experimental procedure. In one instance, the precipitate was weighed dry and it accounted for 98 %wt of the theoretical amount. For the characterization of the salt, a small amount of the precipitation was dried and stored under an argon atmosphere to ensure a low degree of oxidation before submitting the sample to mass spectrometry (ESI). The spontanous oxidation of *p*-toluenesulfinates to *p*-toluenesulfinatessulfonates have been noted by others.[28] The ^{1}H-NMR shows a mixture of 1,1,3,3-tetramethylguanidine *p*-toluenesulfinate and 1,1,3,3-tetramethylguanidine *p*-toluenesulfonate (a tentative assignment of the peaks can be found in the spectrum on page 69).

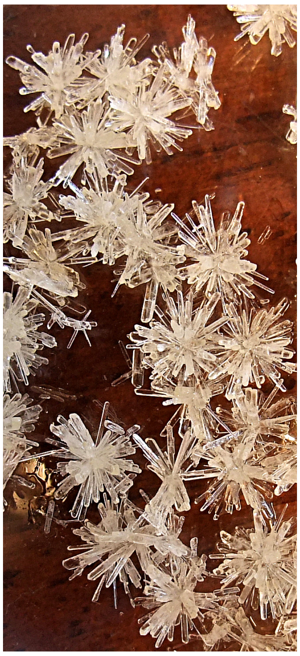

Physical data

- ^{1}H-NMR (200 MHz, $CDCl_3$) δ 8.92 (bs), 7.72 (m), 7.53 (m), 7.11 (m), 7.08 (m), 3.87 (bs), 2.80 (s), 2.28 (s)
- MS (ESI) (H_2O) (ES+) *m/z* 510/508/506 $[2\,TMG + 2\,HBr + H]^+$, 403.4 $[2\,TMG + p{-}Ts{-}SO_3H + H]^+$, 387.5 $[2\,TMG + p{-}Ts{-}SO_2H + H]^+$, 313/311 $[2\,TMG + HBr]^+$, 231.3 $[2\,TMG + H]^+$, 116.2 $[TMG + H]^+$
- MS (ESI) (H_2O) (ES-) *m/z* 171 $[p{-}Ts{-}SO_3]^-$, 155 $[p{-}Ts{-}SO_2]^-$

References

[1] H. E. Gottlieb, V. Kotlyar, A. Nudelman, *J. Org. Chem.* **1997**, *62*, 7512–7515

[2] a) B. P. Axe, *J. Pharm. Biomed. Anal.* **2006**, *41*, 804–810
b) A. T. Lebedev, *Mass Spectrom. Rev.* **1991**, *10*, 91–132

[3] E. M. Tippmann, M. S. Platz, I. B. Svir, O. V. Klymenko, *J. Am. Chem. Soc.* **2004**, *126*, 5750–5762

[4] S. P. L. de Souza, J. F. M. da Silva, M. C. S. de Mattos, *Synth. Commun.* **2003**, *33*, 935–939

[5] J.-J. Kim, D.-H. Kweon, S.-D. Cho, H.-K. Kim, S.-G. Lee, Y.-J. Yoon, *Synlett* **2006**, *2*, 194–200

[6] H. J. Dauben, Jr., L. L. McCoy, *J. Am. Chem. Soc.* **1959**, *81*, 4863–4873

[7] T. Toma, J. Shimokawa, T. Fukuyama, *Org. Lett.* **2007**, *9*, 3195–3197

[8] S. K. Vasisht, P. Venugopalan, J. Kataria, A. Sharma, *Indian J. Chem.* **2002**, *41A*, 2054–2059

[9] L. Zhang, X. jun Wang, J. Wang, N. Grinberg, D. Krishnamurthy, C. H. Senanayake, *Tetrahedron Lett.* **2009**, *50*, 2964–2966

[10] I. Leito, *Acidity-Basicity Data (pKa Values) in Nonaqueous Solvents*, `http://tera.chem.ut.ee/~ivo/HA_UT/`

[11] a) S. M. Ahmad, D. C. Braddock, G. Cansell, S. A. Hermitage, *Tetrahedron Lett.* **2007**, *48*, 915–918
b) R. S. Grainger, N. E. Leadbeater, A. M. Pàmies, *Catal. Commun.* **2002**, *3*, 449–452

[12] J. V. Ruppel, T. J. Gauthier, N. L. Snyder, J. A. Perman, X. P. Zhang, *Org. Lett.* **2009**, *11*, 2273–2276

[13] V. K. Aggarwal, P. Blackburn, R. Fieldhouse, R. V. H. Jones, *Tetrahedron Lett.* **1998**, *39*, 8517–8520

[14] R. H. Earle, D. T. Hurst, M. Viney, *Journal of the Chemical Society C: Organic* **1969**, *0*, 2093–2098

[15] M. Regitz, J. Hocker, A. Liedhegener, *Org. Prep. Proced.* **1969**, *1*, 99–104

[16] G. Lowe, J. Parker, *J. Chem. Soc. D* **1971**, *18*, 1135–1136

[17] N. P. Peet, S. Sunder, *J. Heterocycl. Chem.* **1975**, *12*, 1191–1197

[18] J. S. Wiering, H. Wynberg, *J. Org. Chem.* **1976**, *41*, 1574–1578

[19] K. Bott, *Chem. Ber.* **1987**, *120*, 1867–1871

[20] A. Ouihia, L. Rene, J. Guilhem, C. Pascard, B. Badet, *J. Org. Chem.* **1993**, *58*, 1641–1642

[21] M. P. Doyle, A. V. Kalinin, *Synlett* **1995**, *1995*, 1075–1076

[22] Å. Kaupang, C. H. Görbitz, T. Hansen, *Acta Crystallogr. Sect. E: Struct. Rep. Online* **2010**, *66*, o1299

[23] A. Saghatelian, J. Buriak, V. S. Y. Lin, M. R. Ghadiri, *Tetrahedron* **2001**, *57*, 5131–5136

[24] Å. Kaupang, C. H. Görbitz, T. Hansen, *Acta Crystallogr. Sect. E: Struct. Rep. Online* **2011**, *67*, o1844–o1845

[25] U. Schöllkopf, M. Reetz, *Tetrahedron Lett.* **1969**, *20*, 1541–1544

[26] A. R. Chakravarty, F. A. Cotton, D. A. Tocher, J. H. Tocher, *Organometallics* **1985**, *4*, 8–13

[27] M. P. Doyle, L. J. Westrum, W. N. E. Wolthuis, M. M. See, W. P. Boone, V. Bagheri, M. M. Pearson, *J. Am. Chem. Soc.* **1993**, *115*, 958–964

[28] F. C. Whitmore, F. H. Hamilton, *Org. Synth.* **1922**, *2*, 89

NMR Spectra

Acquisition Time (sec)	2.9999	***Frequency (MHz)***	200.13	***Nucleus***	1H	***Number of Transients***	16	***Origin***	DPX200
Original Points Count	12417	***Points Count***	16384	***Pulse Sequence***	zg30	***Receiver Gain***	1625.50	***SW(cyclical) (Hz)***	4139.07
Solvent	CHLOROFORM-d			***Spectrum Offset (Hz)***	1233.7882			***Spectrum Type***	STANDARD
Sweep Width (Hz)	4138.82	***Temperature (degree C)***	25.160						

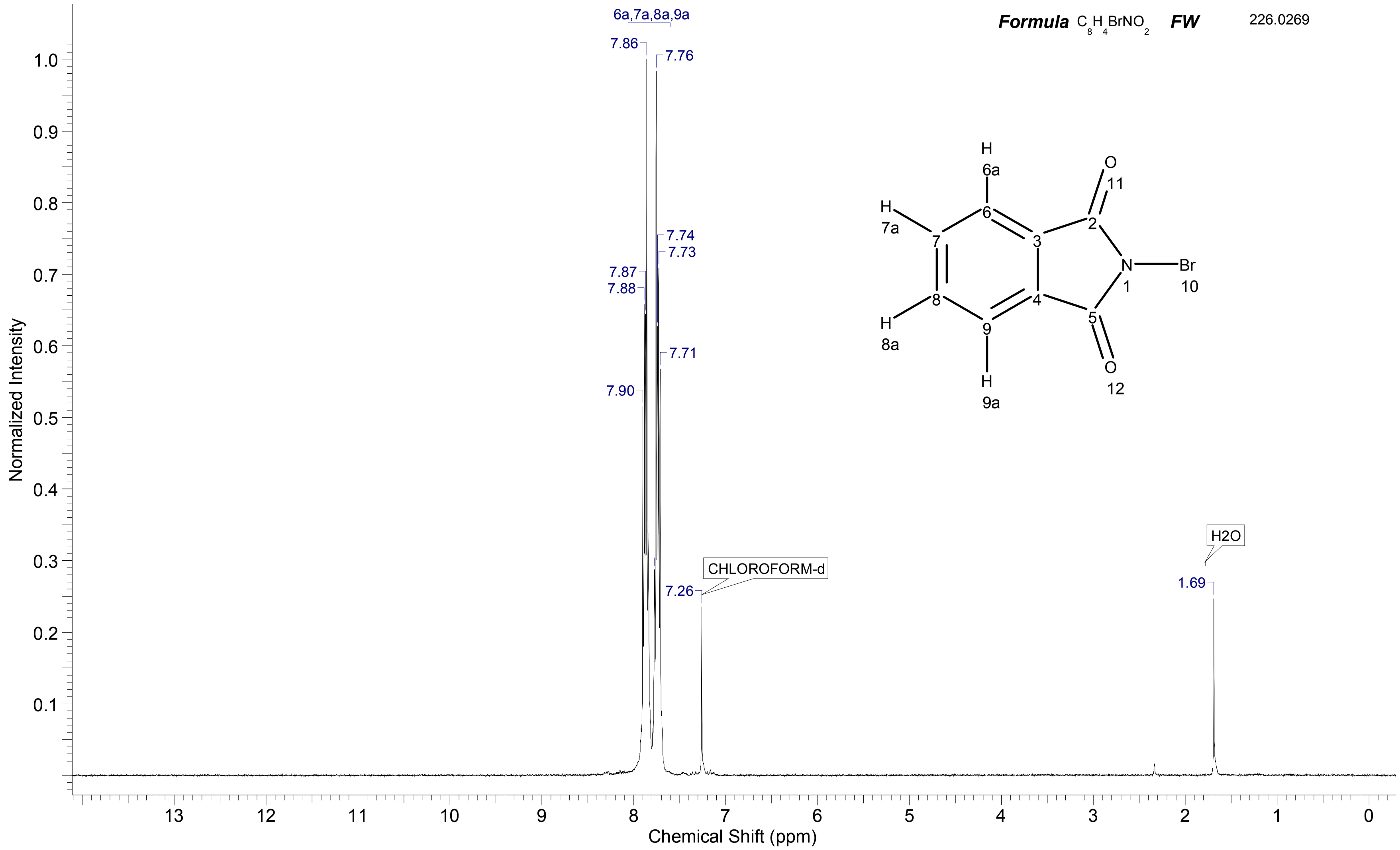

Acquisition Time (sec)	5.3084	***Comment***	PROTON DMSO D: {UIO\aasmuk} 31			***Frequency (MHz)***	300.13	***Nucleus***	1H
Number of Transients	16	***Origin***	DPX300	***Original Points Count***	32768	***Points Count***	32768	***Pulse Sequence***	zg30
Receiver Gain	161.30	***SW(cyclical) (Hz)***	6172.84	***Solvent***	DMSO-d6	***Spectrum Offset (Hz)***	1852.0708	***Spectrum Type***	STANDARD
Sweep Width (Hz)	6172.65	***Temperature (degree C)***	25.160						

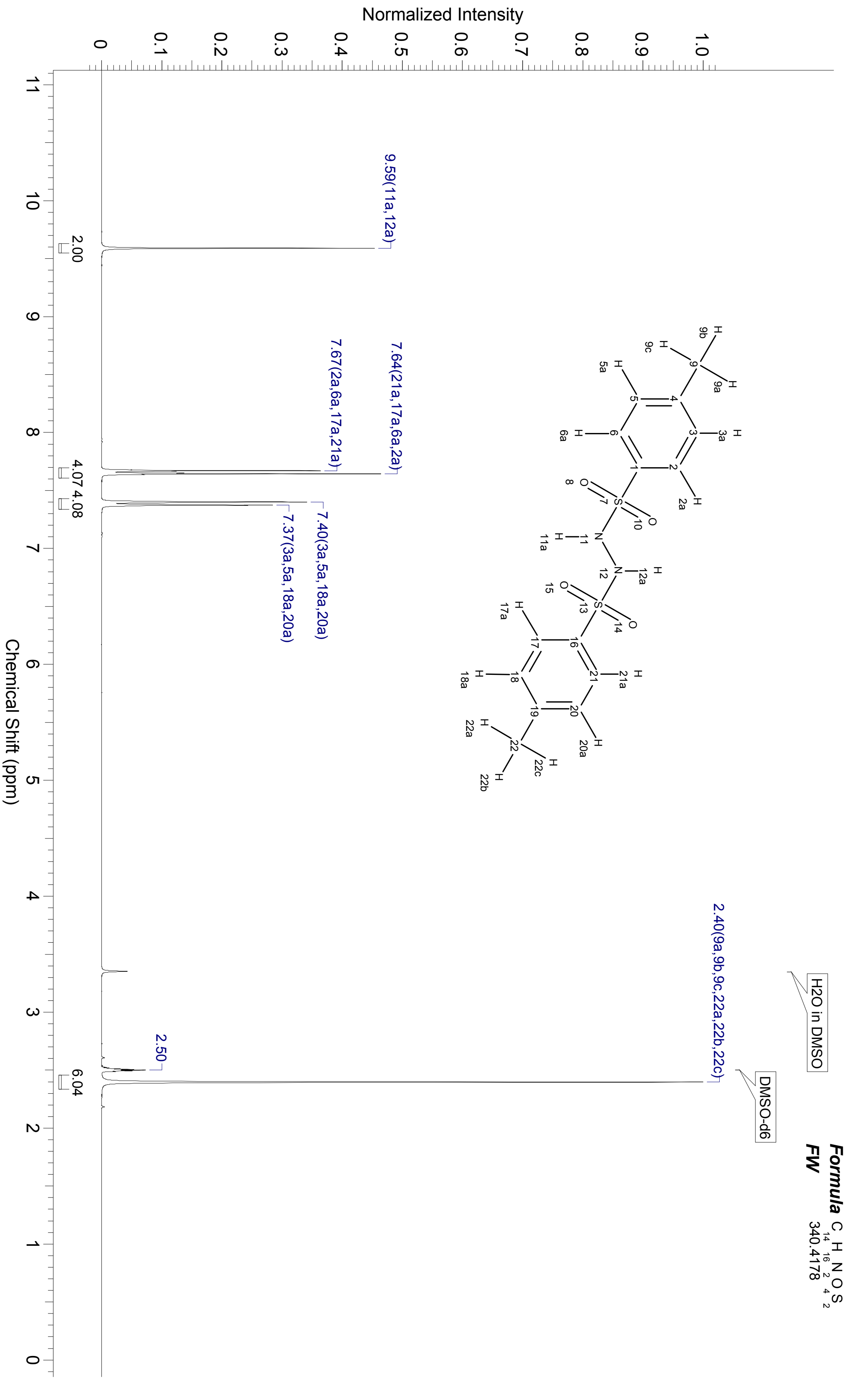

Acquisition Time (sec)	1.8219	***Comment***	C13CPD DMSO D: {UIO\aasmuk} 31			***Frequency (MHz)***	75.48	***Nucleus***	13C
Number of Transients	512	***Origin***	DPX300	***Original Points Count***	32768	***Points Count***	32768	***Pulse Sequence***	zgpg30
Receiver Gain	3649.10	***SW(cyclical) (Hz)***	17985.61	***Solvent***	DMSO-d6	***Spectrum Offset (Hz)***	7511.2959	***Spectrum Type***	STANDARD
Sweep Width (Hz)	17985.06	***Temperature (degree C)***	25.160						

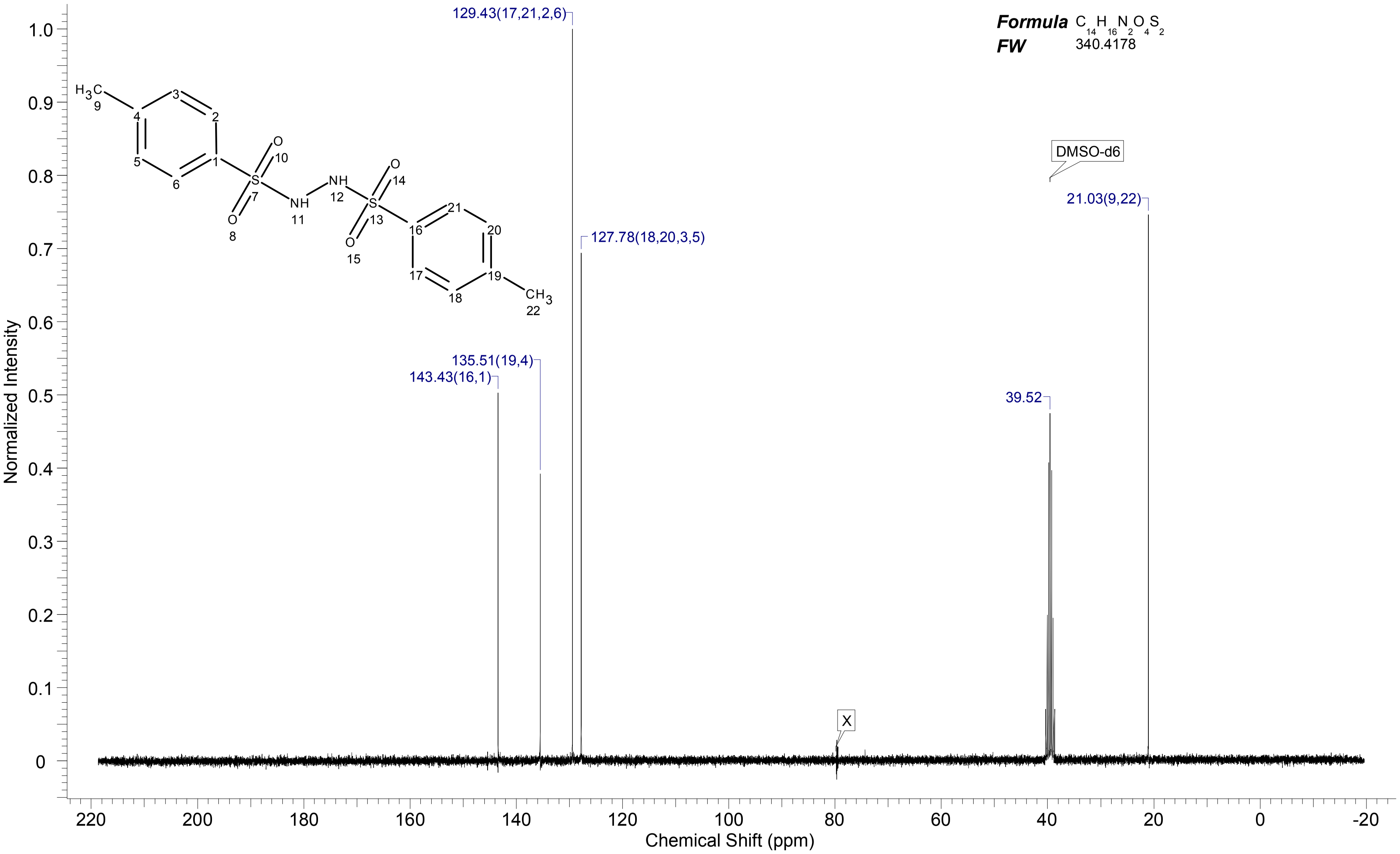

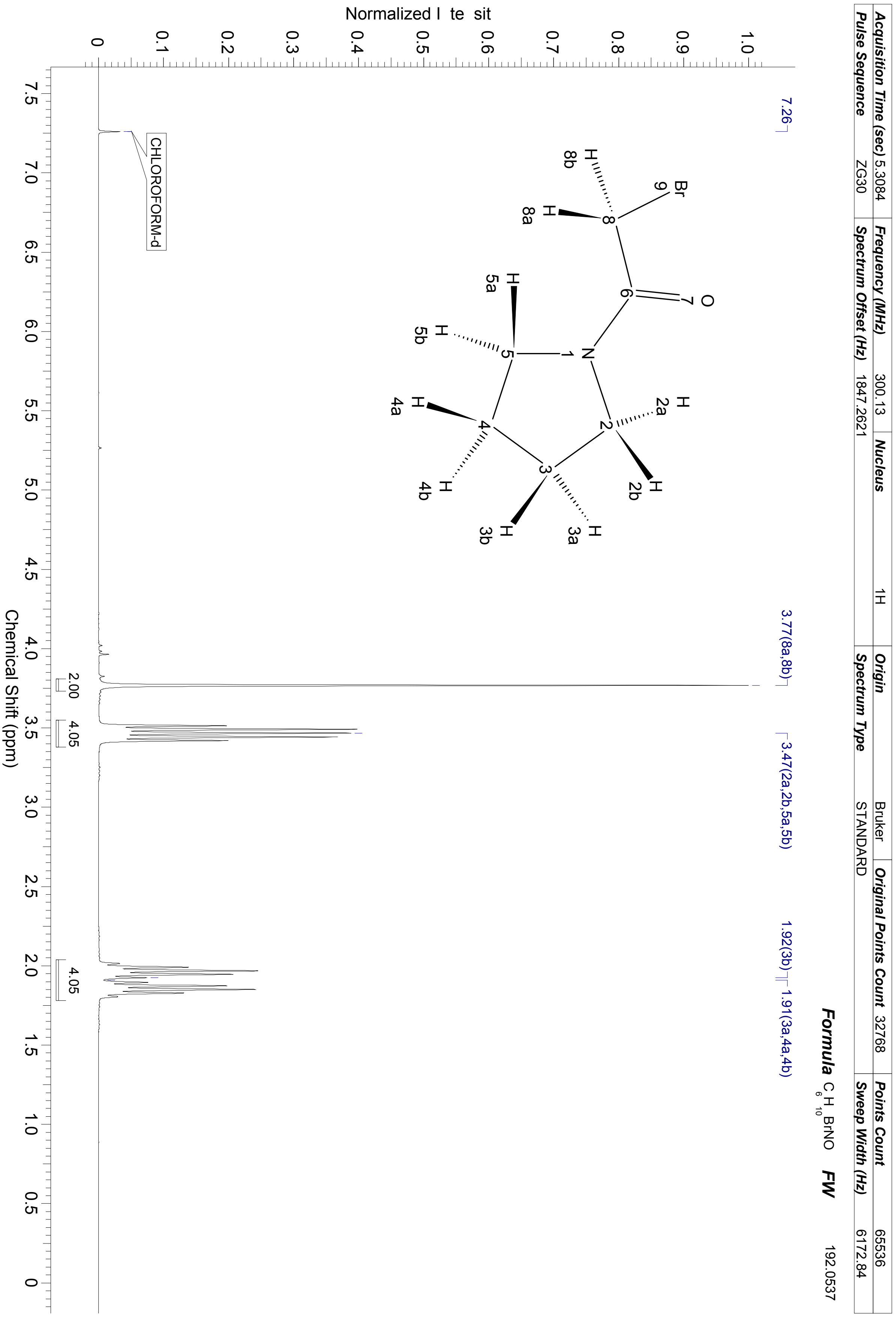
Acquisition Time (sec) 5.3084
Frequency (MHz) 300.13
Nucleus 1H
Origin Bruker
Original Points Count 32768
Points Count 65536
Pulse Sequence ZG30
Spectrum Offset (Hz) 1847.2621
Spectrum Type STANDARD
Sweep Width (Hz) 6172.84
Formula C6H10BrNO FW 192.0537
7.26
3.77(8a,8b)
3.47(2a,2b,5a,5b)
1.92(3b)
1.91(3a,4a,4b)
CHLOROFORM-d
2.00
4.05
4.05
Chemical Shift (ppm)

Acquisition Time (sec)	1.8219	***Comment***	C13CPD CDCl3 D: {UIO\aasmuk} 50			***Frequency (MHz)***	75.48	***Nucleus***	13C
Number of Transients	1024	***Origin***	DPX300	***Original Points Count***	32768	***Points Count***	32768	***Pulse Sequence***	zgpg30
Receiver Gain	11585.20	***SW(cyclical) (Hz)***	17985.61	***Solvent***	CHLOROFORM-d			***Spectrum Offset (Hz)***	7538.0913
Spectrum Type	STANDARD			***Sweep Width (Hz)***	17985.06	***Temperature (degree C)***	25.160		

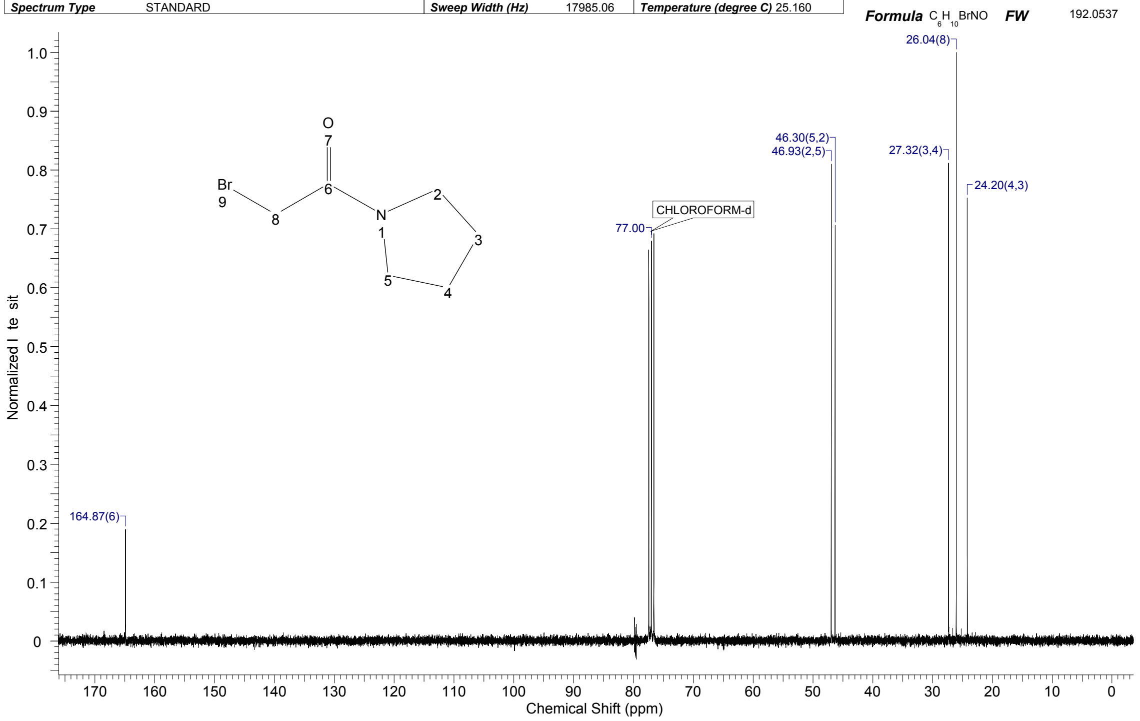

Acquisition Time (sec)	1.8219	***Comment***	C13DEPT135 CDCl3 D: {UIO\aasmuk} 50			***Frequency (MHz)***	75.48	***Nucleus***	13C
Number of Transients	256	***Origin***	DPX300	***Original Points Count***	32768	***Points Count***	32768	***Pulse Sequence***	dept135
Receiver Gain	16384.00	***SW(cyclical) (Hz)***	17985.61	***Solvent***	CHLOROFORM-d			***Spectrum Offset (Hz)***	7538.3940
Spectrum Type	DEPT135	***Sweep Width (Hz)***	17985.06	***Temperature (degree C)***	25.160				

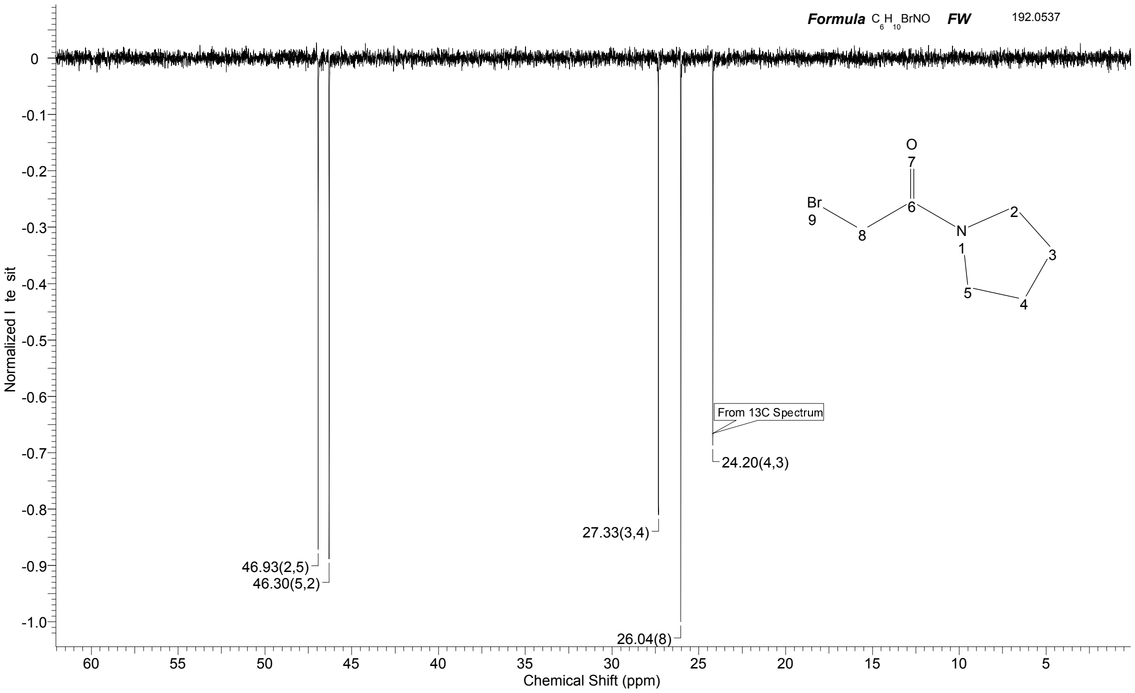

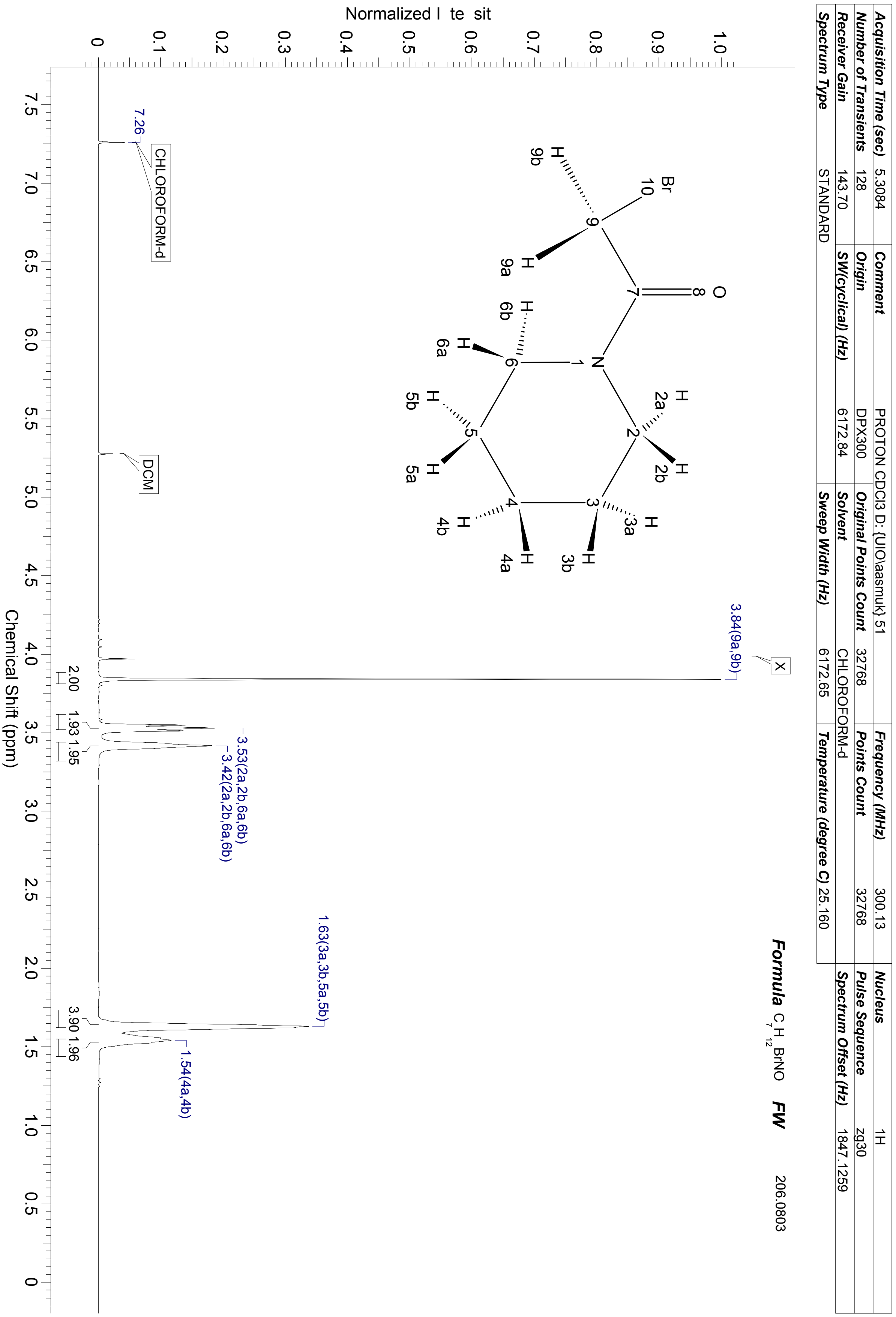
Acquisition Time (sec) 5.3084
Comment PROTON CDCl3 D: {UIO}aasmuk} 51
Frequency (MHz) 300.13
Nucleus 1H
Number of Transients 128
Origin DPX300
Original Points Count 32768
Points Count 32768
Pulse Sequence zg30
Receiver Gain 143.70
SW(cyclical) (Hz) 6172.84
Solvent CHLOROFORM-d
Spectrum Offset (Hz) 1847.1259
Spectrum Type STANDARD
Sweep Width (Hz) 6172.65
Temperature (degree C) 25.160
Formula C7H12BrNO FW 206.0803
3.84(9a,9b)
3.53(2a,2b,6a,6b)
3.42(2a,2b,6a,6b)
1.63(3a,3b,5a,5b)
1.54(4a,4b)
7.26
CHLOROFORM-d
DCM
X
2.00
1.93
1.95
3.90
1.96
Normalized Intensity
Chemical Shift (ppm)

Acquisition Time (sec)	1.8219	***Comment***	C13CPD CDCl3 D: {UIO\aasmuk} 51			***Frequency (MHz)***	75.48	***Nucleus***	13C
Number of Transients	1024	***Origin***	DPX300	***Original Points Count***	32768	***Points Count***	32768	***Pulse Sequence***	zgpg30
Receiver Gain	11585.20	***SW(cyclical) (Hz)***	17985.61	***Solvent***	CHLOROFORM-d			***Spectrum Offset (Hz)***	7540.2866
Spectrum Type	STANDARD			***Sweep Width (Hz)***	17985.06	***Temperature (degree C)***	25.160		

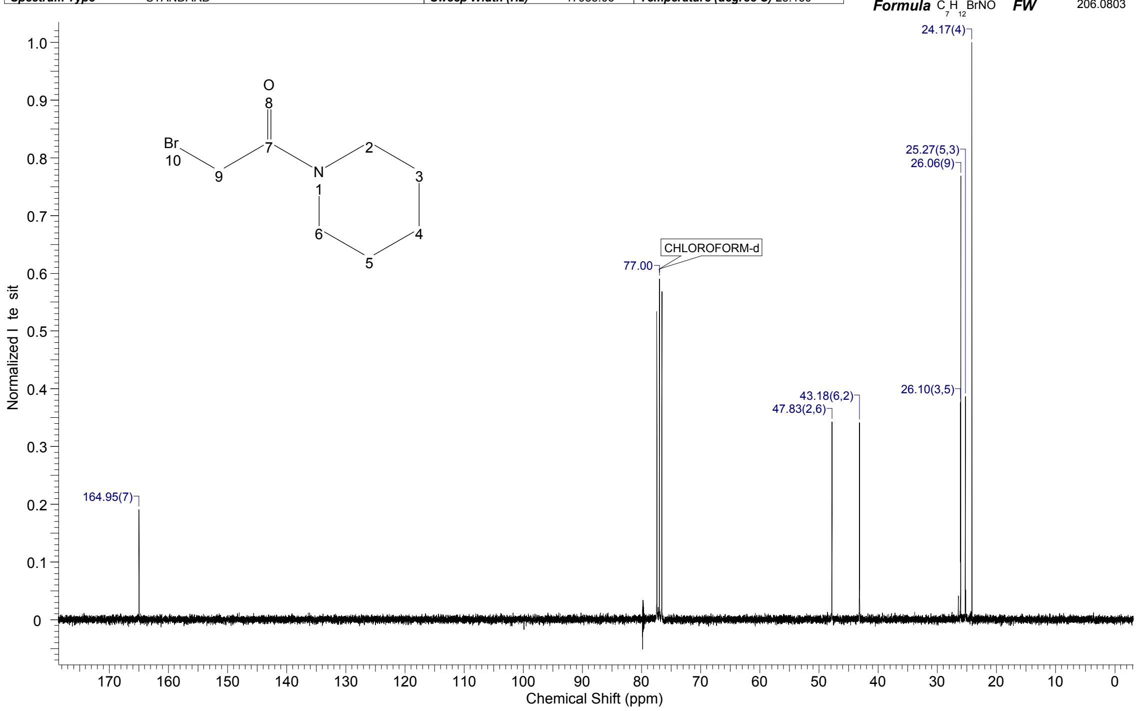

Acquisition Time (sec)	1.8219	***Comment***	C13DEPT135 CDCl3 D: {UIO\aasmuk} 51			***Frequency (MHz)***	75.48	***Nucleus***	13C
Number of Transients	256	***Origin***	DPX300	***Original Points Count***	32768	***Points Count***	32768	***Pulse Sequence***	dept135
Receiver Gain	16384.00	***SW(cyclical) (Hz)***	17985.61	***Solvent***	CHLOROFORM-d			***Spectrum Offset (Hz)***	7539.9717
Spectrum Type	DEPT135	***Sweep Width (Hz)***	17985.06	***Temperature (degree C)***	25.160				

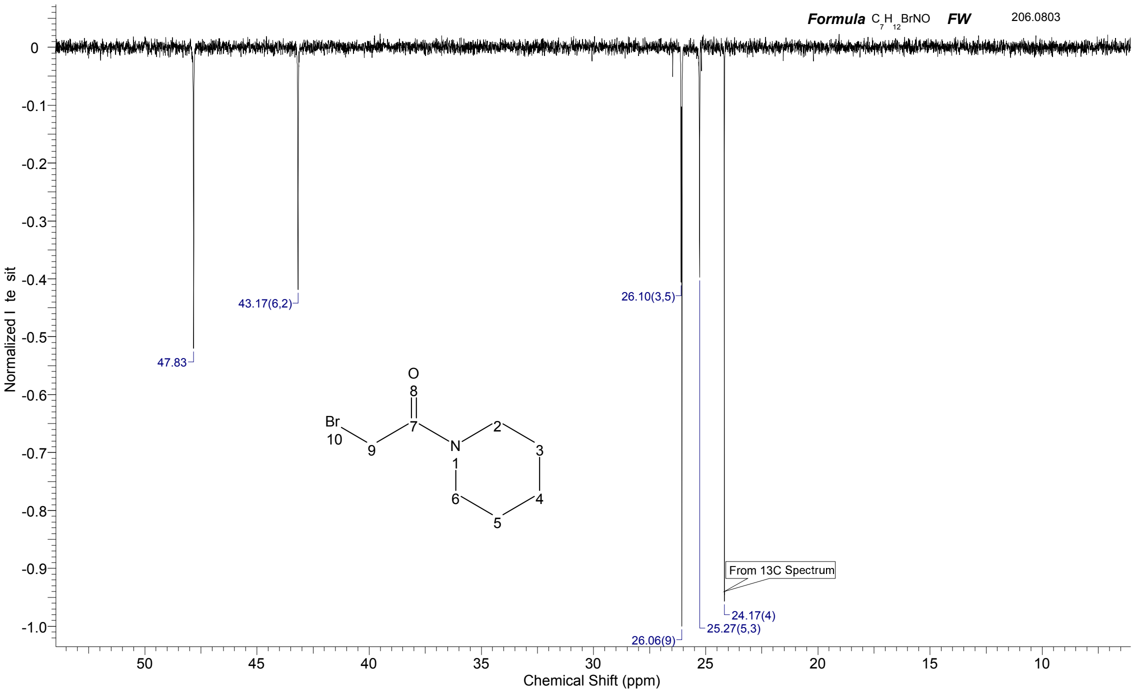

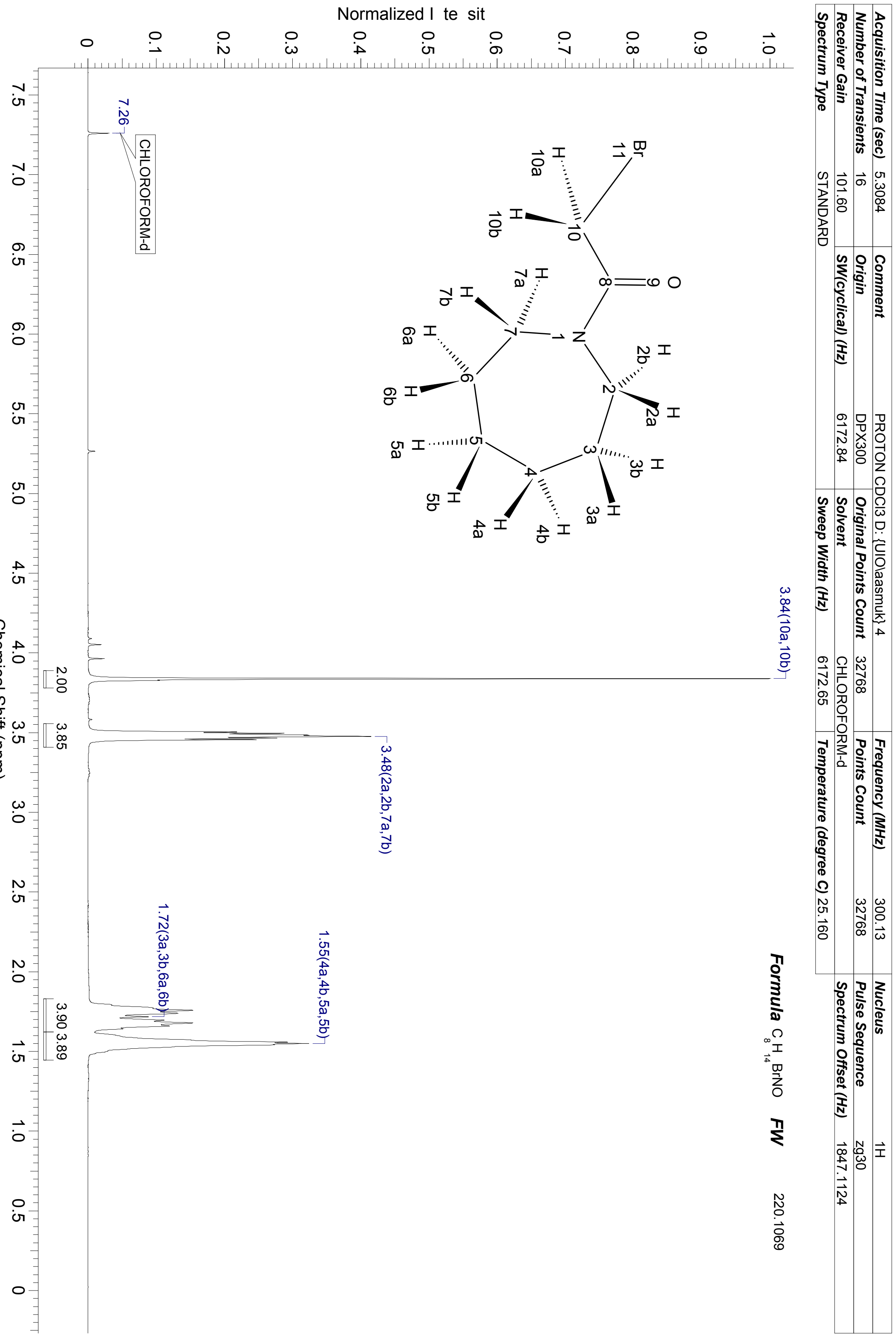
Acquisition Time (sec) 5.3084
Comment PROTON CDCl3 D: {UIO\aasmuk} 4
Frequency (MHz) 300.13
Nucleus 1H
Number of Transients 16
Origin DPX300
Original Points Count 32768
Points Count 32768
Pulse Sequence zg30
Receiver Gain 101.60
SW(cyclical) (Hz) 6172.84
Solvent CHLOROFORM-d
Spectrum Offset (Hz) 1847.1124
Spectrum Type STANDARD
Sweep Width (Hz) 6172.65
Temperature (degree C) 25.160
Formula C8H14BrNO FW 220.1069
3.84(10a,10b)
3.48(2a,2b,7a,7b)
1.72(3a,3b,6a,6b)
1.55(4a,4b,5a,5b)
7.26
CHLOROFORM-d
2.00
3.85
3.90 3.89
Normalized Intensity
Chemical Shift (ppm)

Acquisition Time (sec)	1.8219	***Comment***	C13CPD CDCl3 D: {UIO\aasmuk} 4			***Frequency (MHz)***	75.47	***Nucleus***	13C
Number of Transients	1024	***Origin***	DPX300	***Original Points Count***	32768	***Points Count***	32768	***Pulse Sequence***	zgpg30
Receiver Gain	11585.20	***SW(cyclical) (Hz)***	17985.61	***Solvent***	CHLOROFORM-d			***Spectrum Offset (Hz)***	7536.9590
Spectrum Type	STANDARD			***Sweep Width (Hz)***	17985.06	***Temperature (degree C)***	25.160		

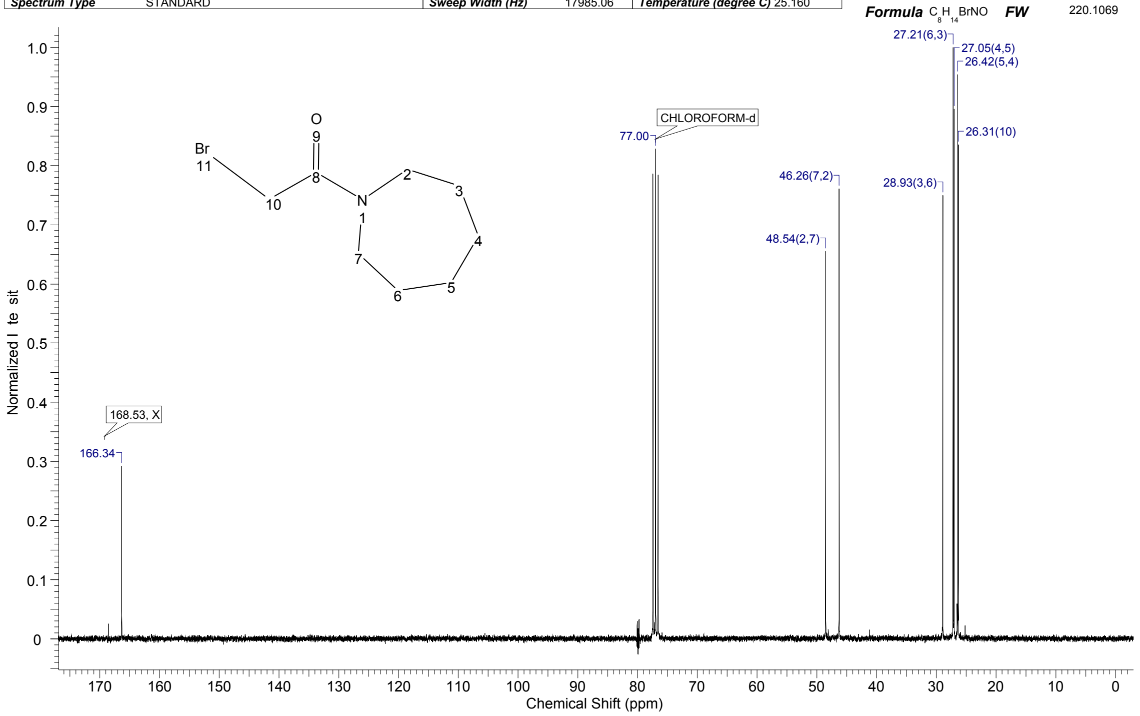

Acquisition Time (sec)	1.8219	***Comment***	C13DEPT135 CDCl3 D: {UIO\aasmuk} 4			***Frequency (MHz)***	75.47	***Nucleus***	13C
Number of Transients	256	***Origin***	DPX300	***Original Points Count***	32768	***Points Count***	32768	***Pulse Sequence***	dept135
Receiver Gain	11585.20	***SW(cyclical) (Hz)***	17985.61	***Solvent***	CHLOROFORM-d			***Spectrum Offset (Hz)***	7536.6265
Spectrum Type	DEPT135	***Sweep Width (Hz)***	17985.06	***Temperature (degree C)***	25.160				

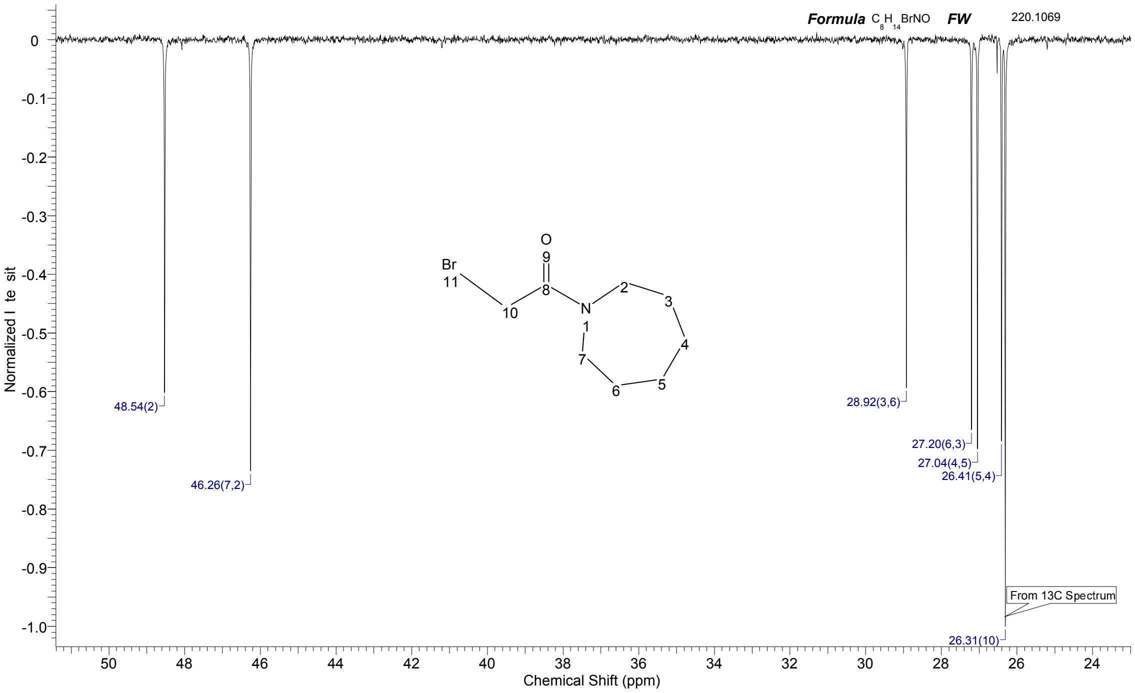

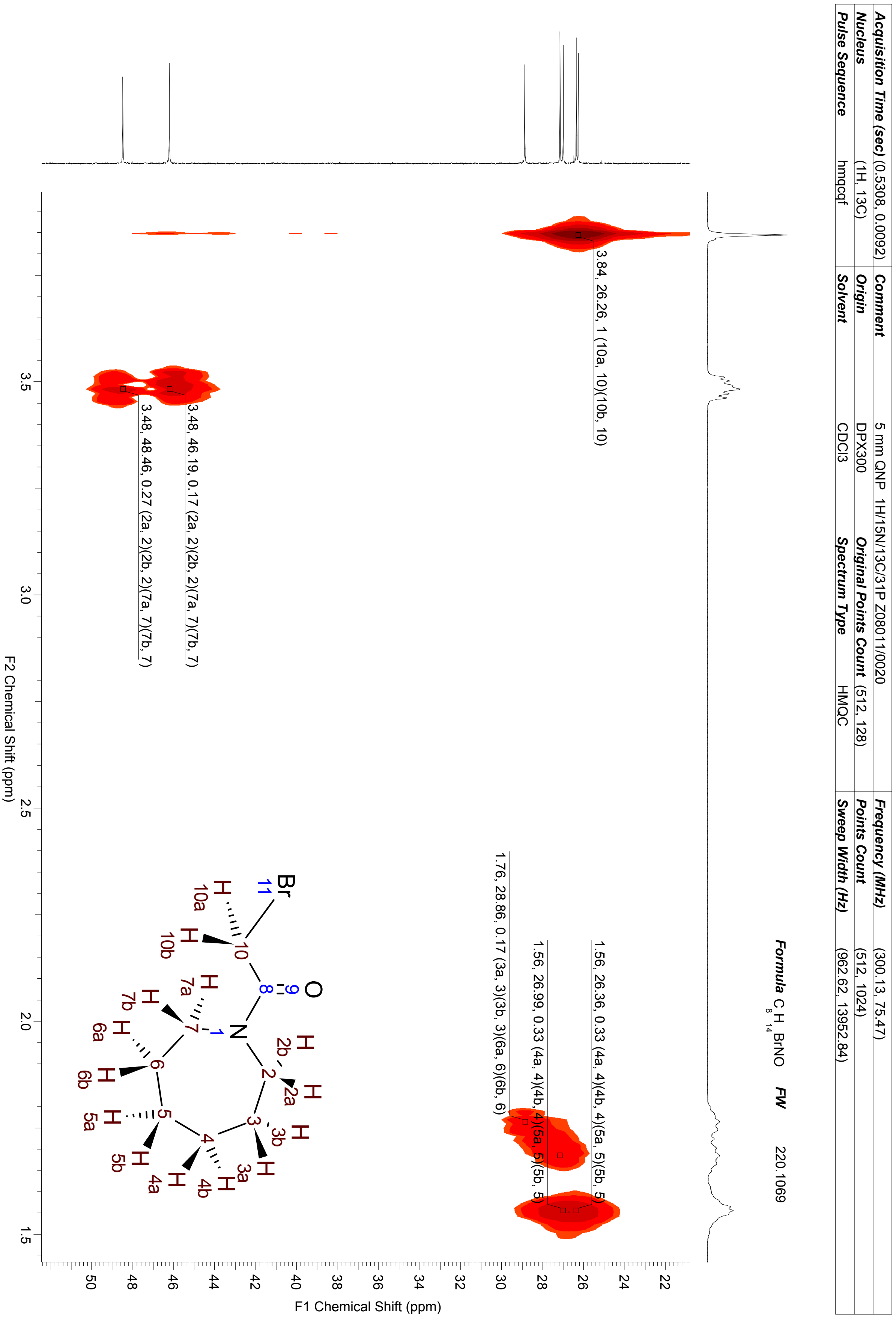
Acquisition Time (sec) (0.5308, 0.0092)
Comment 5 mm QNP 1H/15N/13C/31P Z08011/0020
Frequency (MHz) (300.13, 75.47)
Nucleus (1H, 13C)
Origin DPX300
Original Points Count (512, 128)
Points Count (512, 1024)
Pulse Sequence hmqcgf
Solvent CDCl3
Spectrum Type HMQC
Sweep Width (Hz) (962.62, 13952.84)
Formula C8H14BrNO
FW 220.1069
3.84, 26.26, 1 (10a, 10)(10b, 10)
3.48, 46.19, 0.17 (2a, 2)(2b, 2)(7a, 7)(7b, 7)
3.48, 48.46, 0.27 (2a, 2)(2b, 2)(7a, 7)(7b, 7)
1.56, 26.36, 0.33 (4a, 4)(4b, 4)(5a, 5)(5b, 5)
1.56, 26.99, 0.33 (4a, 4)(4b, 4)(5a, 5)(5b, 5)
1.76, 28.86, 0.17 (3a, 3)(3b, 3)(6a, 6)(6b, 6)
F2 Chemical Shift (ppm)
F1 Chemical Shift (ppm)

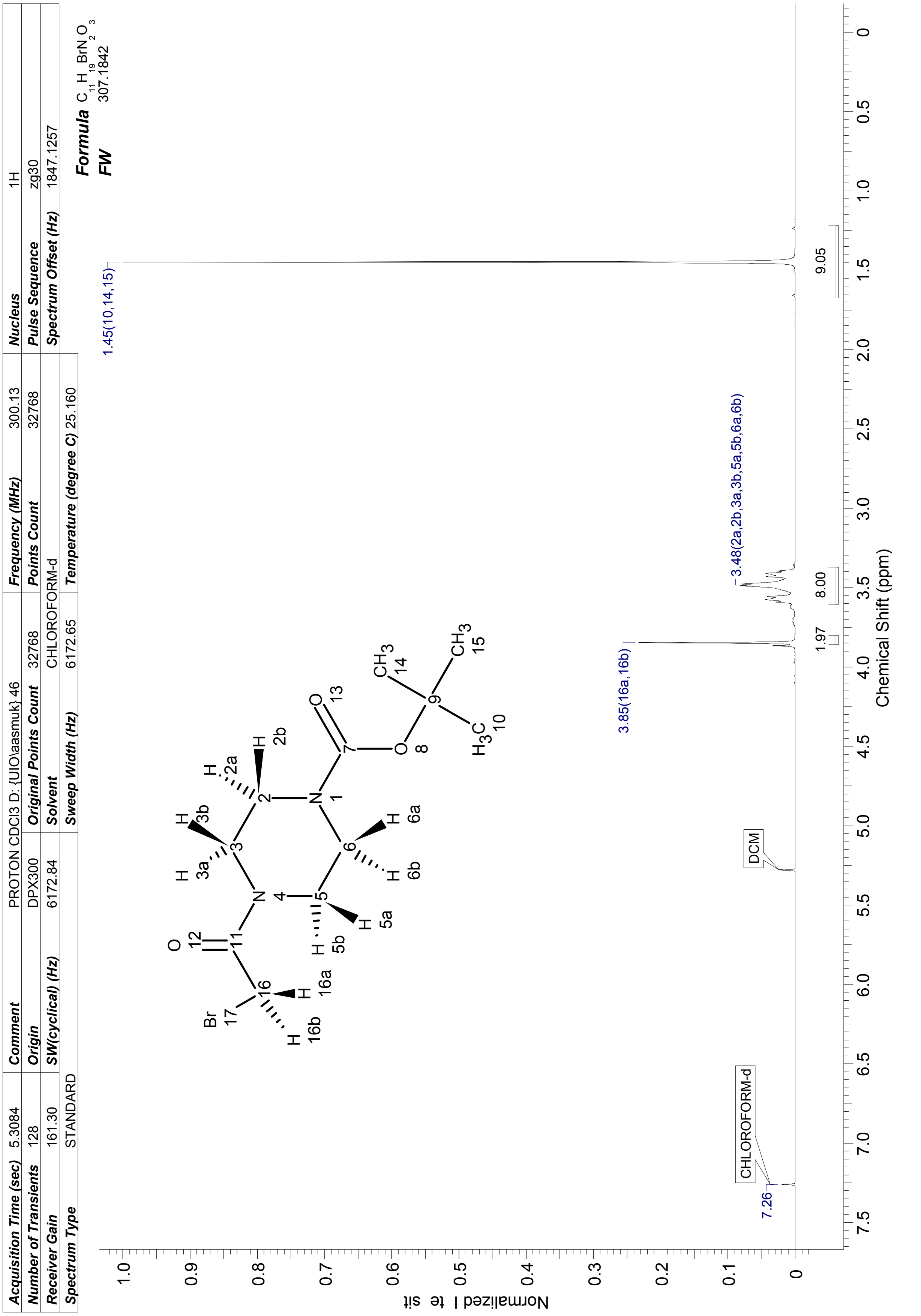

Acquisition Time (sec) 5.3084
Comment PROTON CDCl3 D: {UIO\aasmuk} 46
Frequency (MHz) 300.13
Nucleus 1H
Number of Transients 128
Origin DPX300
Original Points Count 32768
Points Count 32768
Pulse Sequence zg30
Receiver Gain 161.30
SW(cyclical) (Hz) 6172.84
Solvent CHLOROFORM-d
Spectrum Offset (Hz) 1847.1257
Spectrum Type STANDARD
Sweep Width (Hz) 6172.65
Temperature (degree C) 25.160
Formula C11H19BrN2O3
FW 307.1842
1.45(10,14,15)
3.48(2a,2b,3a,3b,5a,5b,6a,6b)
3.85(16a,16b)
7.26
CHLOROFORM-d
DCM
9.05
8.00
1.97
Chemical Shift (ppm)
Normalized Intensity

^{13}C-NMR & DEPT-135 – tert-butyl 4-(2-bromoacetyl)piperazine-1-carboxylate **(2d)**

Acquisition Time (sec)	1.8219	***Comment***	C13CPD CDCl3 D: {UIO\aasmuk} 46			***Frequency (MHz)***	75.48	***Nucleus***	13C
Number of Transients	1024	***Origin***	DPX300	***Original Points Count***	32768	***Points Count***	32768	***Pulse Sequence***	zgpg30
Receiver Gain	13004.00	***SW(cyclical) (Hz)***	17985.61	***Solvent***	CHLOROFORM-d			***Spectrum Offset (Hz)***	7541.9336
Spectrum Type	STANDARD			***Sweep Width (Hz)***	17985.06	***Temperature (degree C)***	25.160		

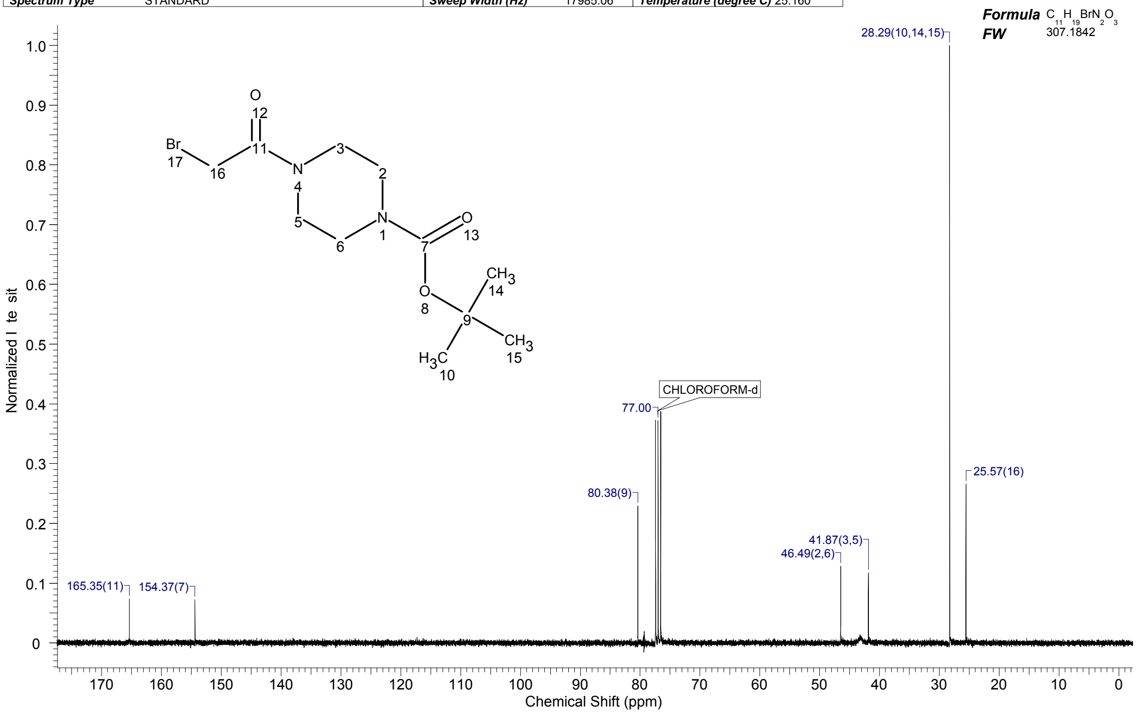

Acquisition Time (sec)	1.8219	***Comment***	C13DEPT135 CDCl3 D: {UIO\aasmuk} 46			***Frequency (MHz)***	75.48	***Nucleus***	13C
Number of Transients	256	***Origin***	DPX300	***Original Points Count***	32768	***Points Count***	32768	***Pulse Sequence***	dept135
Receiver Gain	13004.00	***SW(cyclical) (Hz)***	17985.61	***Solvent***	CHLOROFORM-d			***Spectrum Offset (Hz)***	7541.8994
Spectrum Type	DEPT135	***Sweep Width (Hz)***	17985.06	***Temperature (degree C)***	25.160				

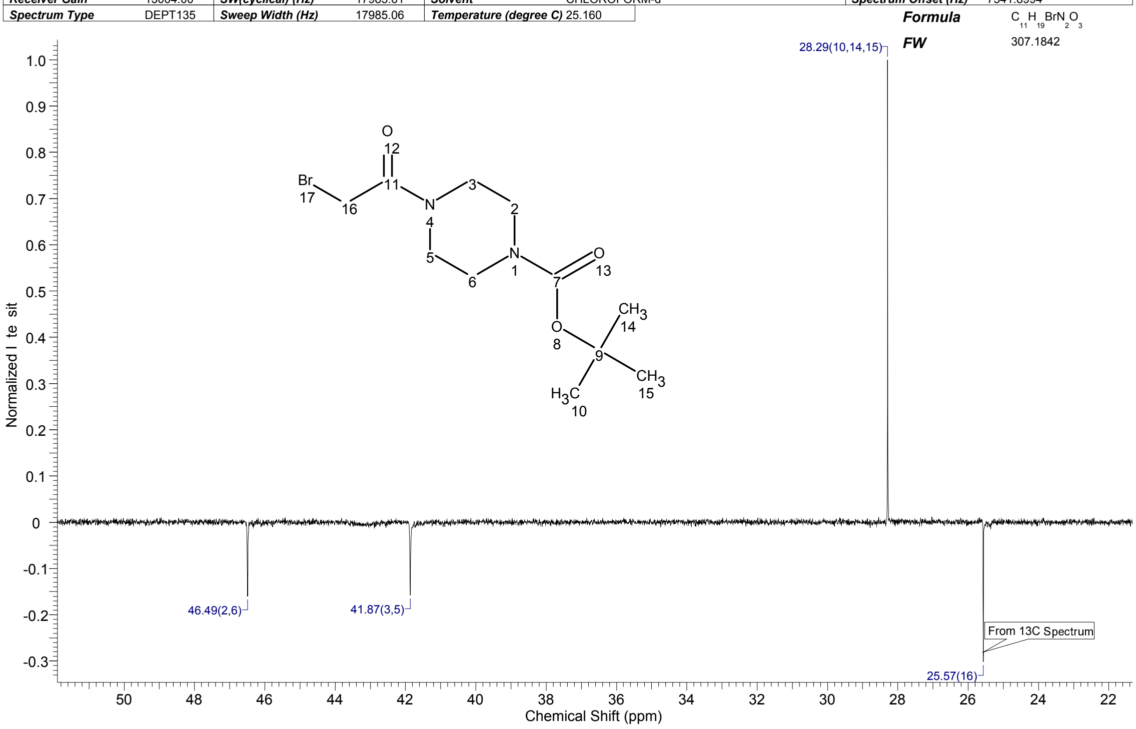

Acquisition Time (sec)	5.3084	***Comment***	PROTON CDCl3 D: {UIO\aasmuk} 52	***Frequency (MHz)***	300.13	***Nucleus***	1H		
Number of Transients	128	***Origin***	DPX300	***Original Points Count***	32768	***Points Count***	32768	***Pulse Sequence***	zg30
Receiver Gain	161.30	***SW(cyclical) (Hz)***	6172.84	***Solvent***	CHLOROFORM-d	***Spectrum Offset (Hz)***	1847.1257		
Spectrum Type	STANDARD	***Sweep Width (Hz)***	6172.65	***Temperature (degree C)***	25.160				

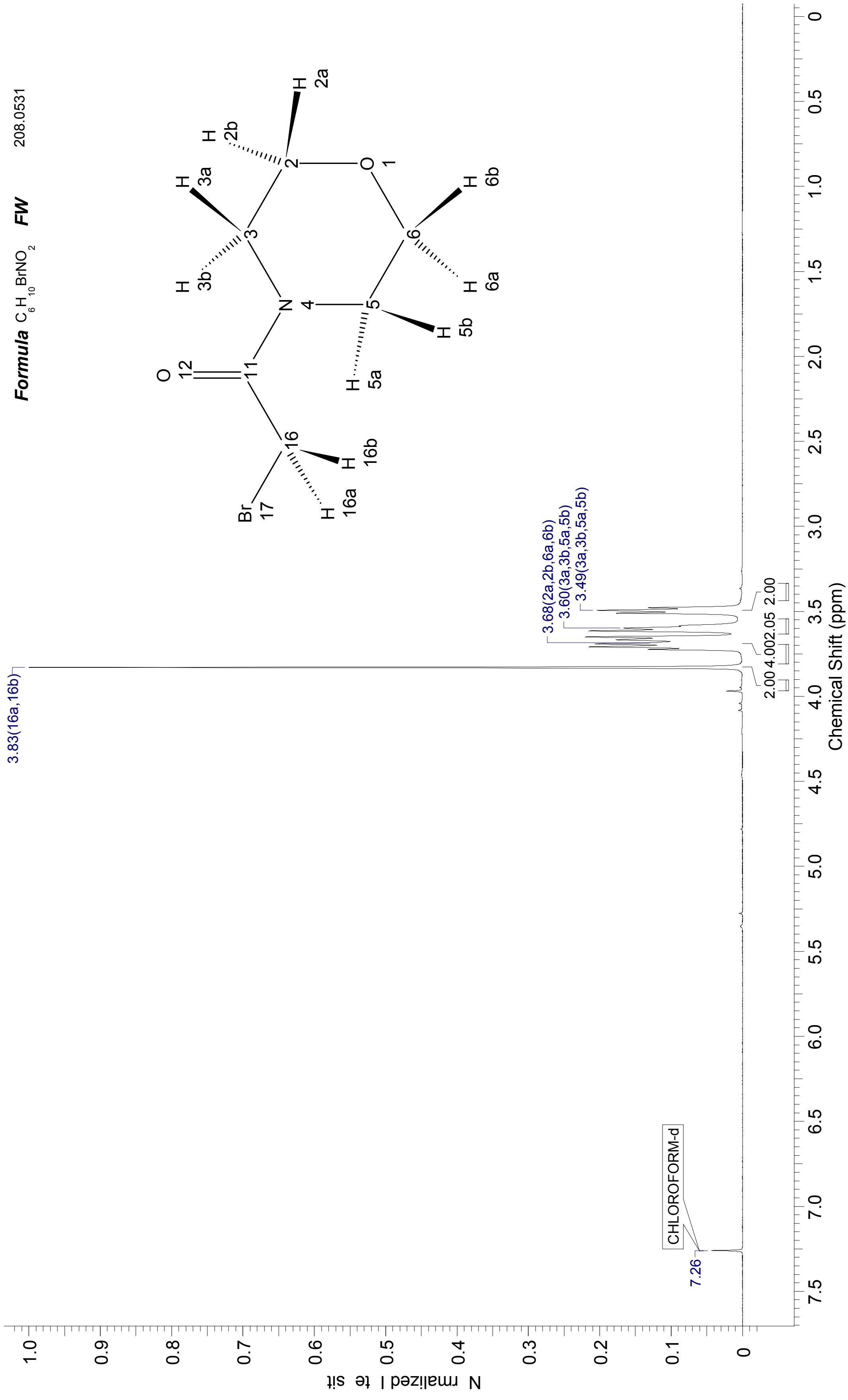

Acquisition Time (sec)	1.8219	***Comment***	C13CPD CDCl3 D: {UIO\aasmuk} 52			***Frequency (MHz)***	75.48	***Nucleus***	13C
Number of Transients	1024	***Origin***	DPX300	***Original Points Count***	32768	***Points Count***	32768	***Pulse Sequence***	zgpg30
Receiver Gain	10321.30	***SW(cyclical) (Hz)***	17985.61	***Solvent***	CHLOROFORM-d			***Spectrum Offset (Hz)***	7539.7378
Spectrum Type	STANDARD			***Sweep Width (Hz)***	17985.06	***Temperature (degree C)***	25.160		

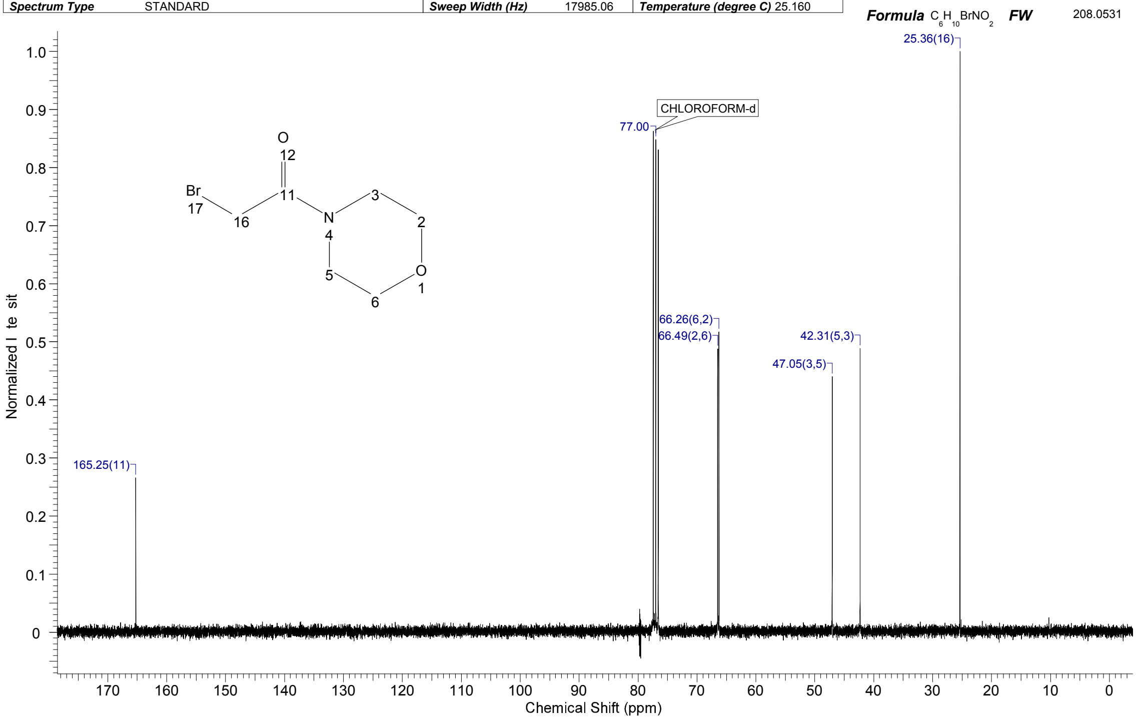

Acquisition Time (sec)	1.8219	***Comment***	C13DEPT135 CDCl3 D: {UIO\aasmuk} 52			***Frequency (MHz)***	75.48	***Nucleus***	13C
Number of Transients	256	***Origin***	DPX300	***Original Points Count***	32768	***Points Count***	32768	***Pulse Sequence***	dept135
Receiver Gain	16384.00	***SW(cyclical) (Hz)***	17985.61	***Solvent***	CHLOROFORM-d			***Spectrum Offset (Hz)***	7539.7715
Spectrum Type	DEPT135	***Sweep Width (Hz)***	17985.06	***Temperature (degree C)***	25.160				

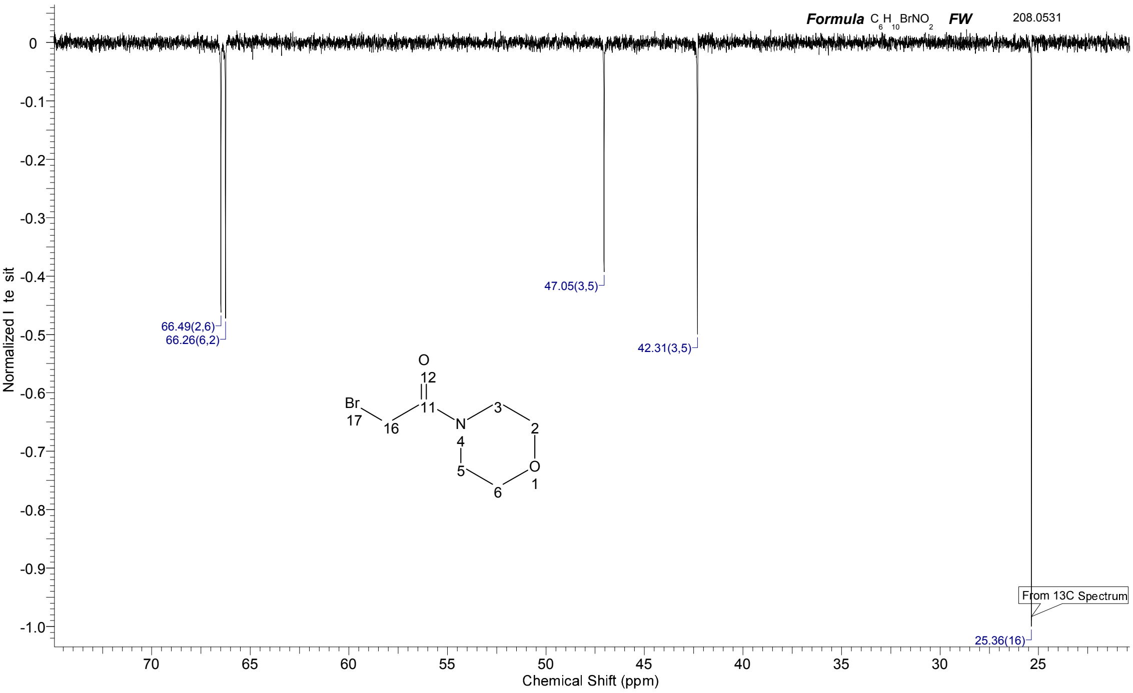

**Acquisition Time (sec)**	5.3084	**Comment**	PROTON CD3CN D: {UIO\aasmuk} 57			**Frequency (MHz)**	300.13
**Number of Transients**	16	**Origin**	DPX300	**Original Points Count**	32768	**Points Count**	32768
**Receiver Gain**	512.00	**SW(cyclical) (Hz)**	6172.84	**Solvent**	ACETONITRILE-d3		
**Spectrum Type**	STANDARD			**Sweep Width (Hz)**	6172.65	**Temperature (degree C)**	25.160

**Nucleus**	1H
**Pulse Sequence**	zg30
**Spectrum Offset (Hz)**	1851.8181

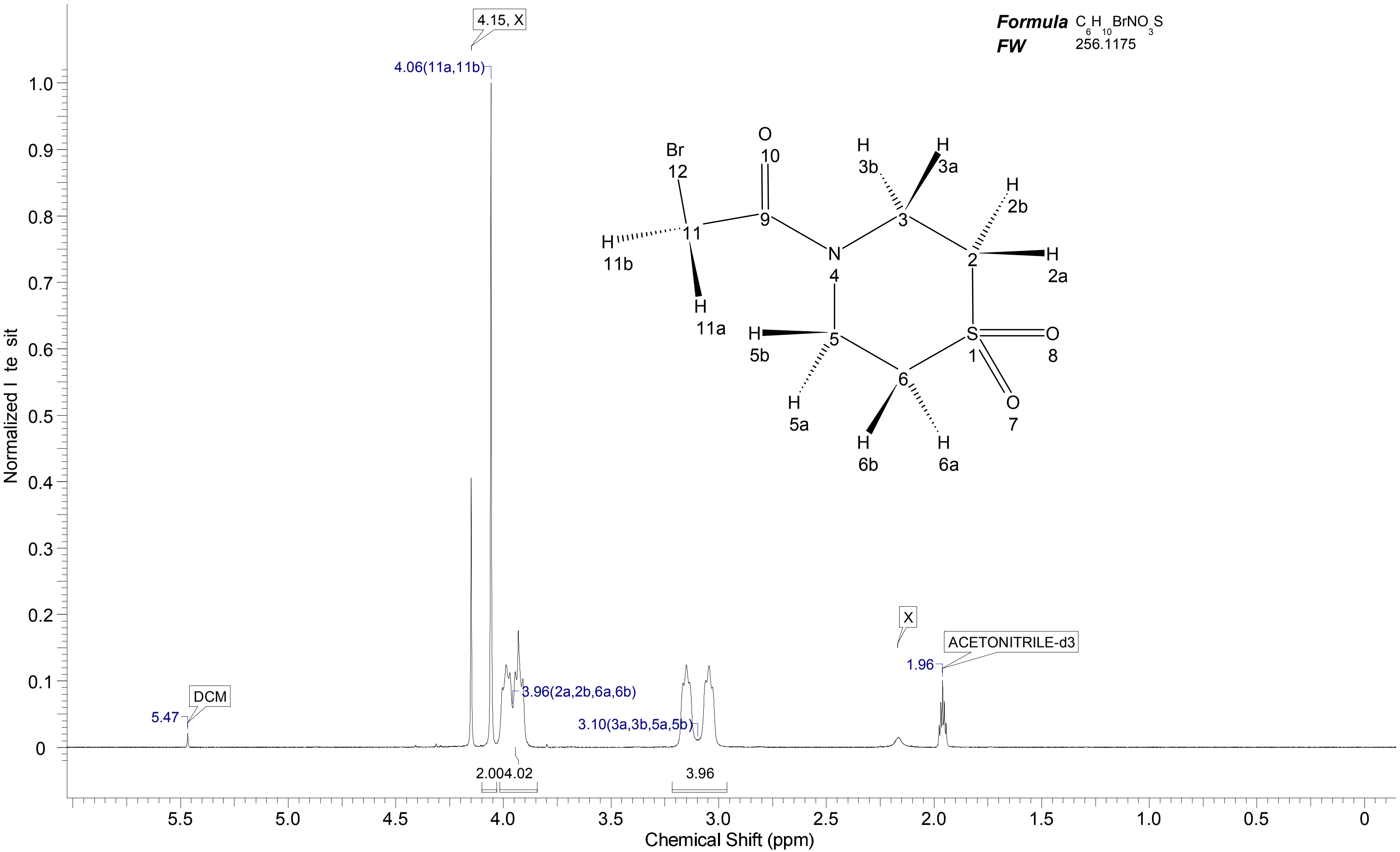

^{13}C-NMR & DEPT-135 – 2-bromo-1-(1,1-dioxidothiomorpholino)ethanone **(2f)**

Acquisition Time (sec)	1.8219	***Comment***	C13CPD CD3CN D: {UIO\aasmuk} 57			***Frequency (MHz)***	75.48	***Nucleus***	13C
Number of Transients	2048	***Origin***	DPX300	***Original Points Count***	32768	***Points Count***	32768	***Pulse Sequence***	zgpg30
Receiver Gain	11585.20	***SW(cyclical) (Hz)***	17985.61	***Solvent***	ACETONITRILE-d3			***Spectrum Offset (Hz)***	7617.4600
Spectrum Type	STANDARD			***Sweep Width (Hz)***	17985.06	***Temperature (degree C)***	25.160		

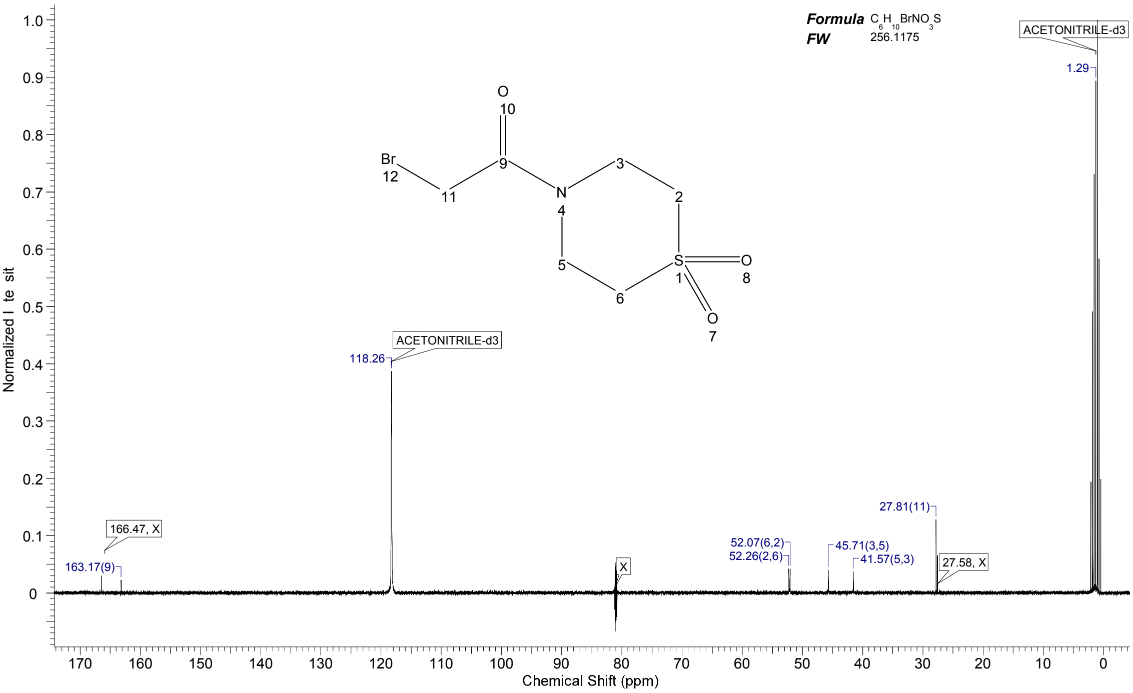

Acquisition Time (sec)	1.8219	***Comment***	C13DEPT135 CD3CN D: {UIO\aasmuk} 57			***Frequency (MHz)***	75.48	***Nucleus***	13C
Number of Transients	512	***Origin***	DPX300	***Original Points Count***	32768	***Points Count***	32768	***Pulse Sequence***	dept135
Receiver Gain	13004.00	***SW(cyclical) (Hz)***	17985.61	***Solvent***	ACETONITRILE-d3			***Spectrum Offset (Hz)***	7617.6548
Spectrum Type	DEPT135	***Sweep Width (Hz)***	17985.06	***Temperature (degree C)***	25.160				

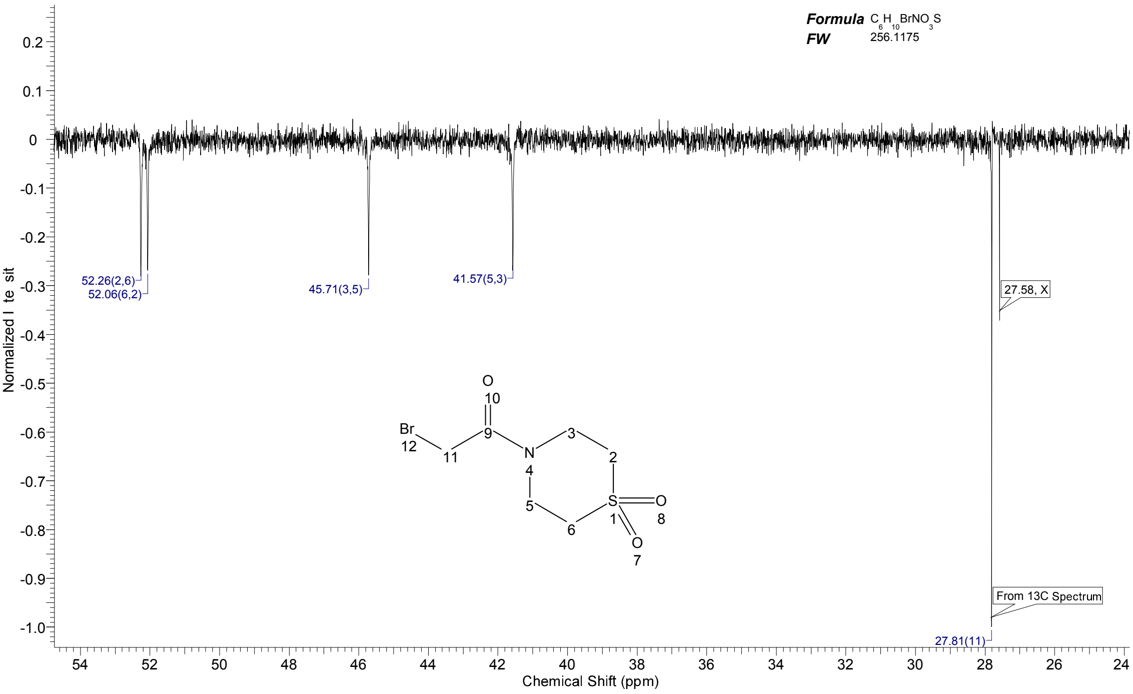

Acquisition Time (sec)	5.3084	***Comment***	5 mm QNP 1H/15N/13C/31P Z08011/0020			***Frequency (MHz)***	300.13	***Nucleus***	1H
Number of Transients	16	***Origin***	DPX300	***Original Points Count***	32768	***Points Count***	32768	***Pulse Sequence***	zg30
Receiver Gain	71.80	***SW(cyclical) (Hz)***	6172.84	***Solvent***	DICHLOROMETHANE-d2			***Spectrum Offset (Hz)***	1842.9987
Spectrum Type	STANDARD			***Sweep Width (Hz)***	6172.65	***Temperature (degree C)***	26.160		

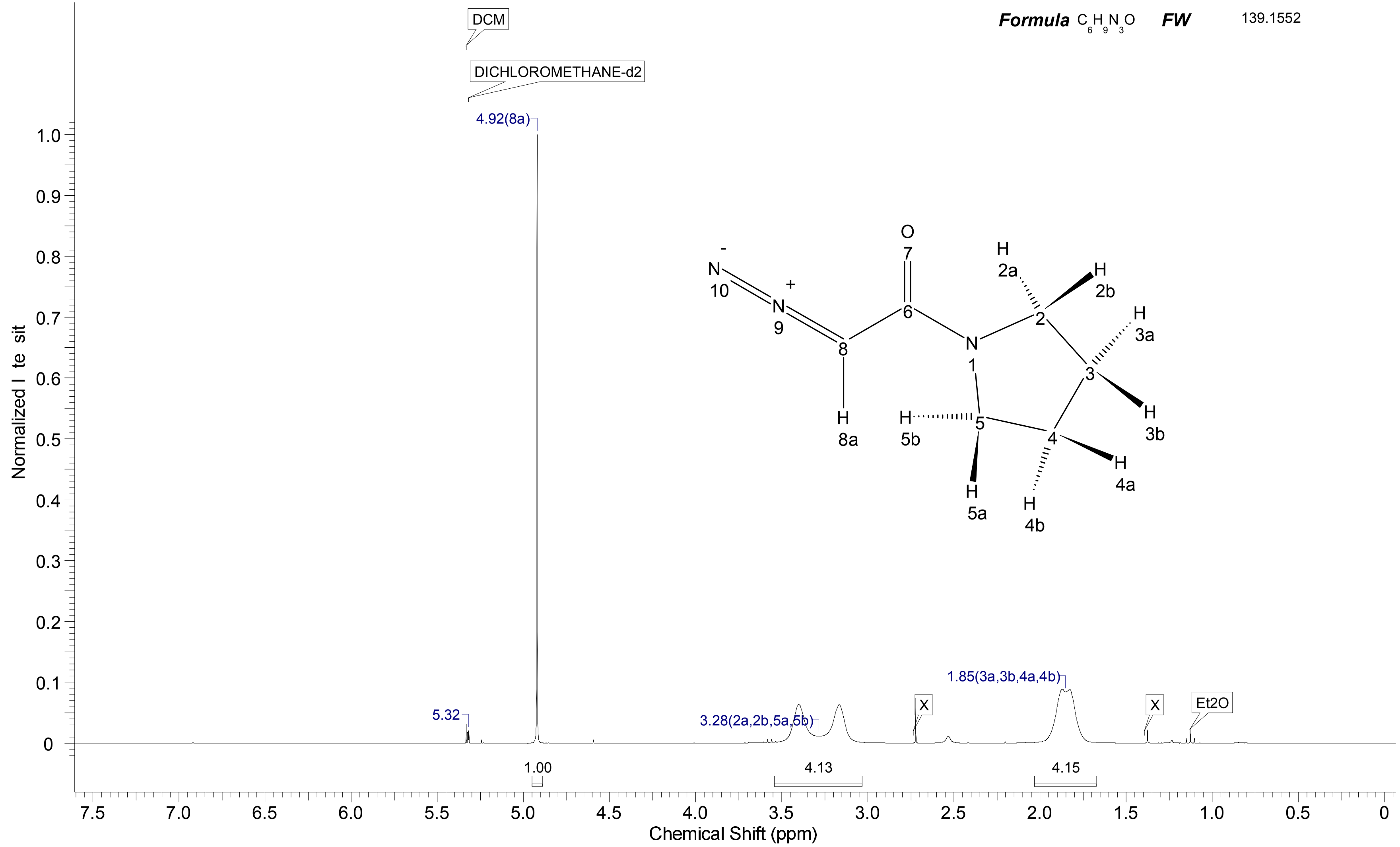

Acquisition Time (sec)	1.8219	***Comment***	C13CPD CD2Cl2 D: {UIO\aasmuk} 27			***Frequency (MHz)***	75.48	***Nucleus***	13C
Number of Transients	1024	***Origin***	DPX300	***Original Points Count***	32768	***Points Count***	32768	***Pulse Sequence***	zgpg30
Receiver Gain	11585.20	***SW(cyclical) (Hz)***	17985.61	***Solvent***	DICHLOROMETHANE-d2			***Spectrum Offset (Hz)***	7568.7197
Spectrum Type	STANDARD	***Sweep Width (Hz)***	17985.06	***Temperature (degree C)***	25.160				

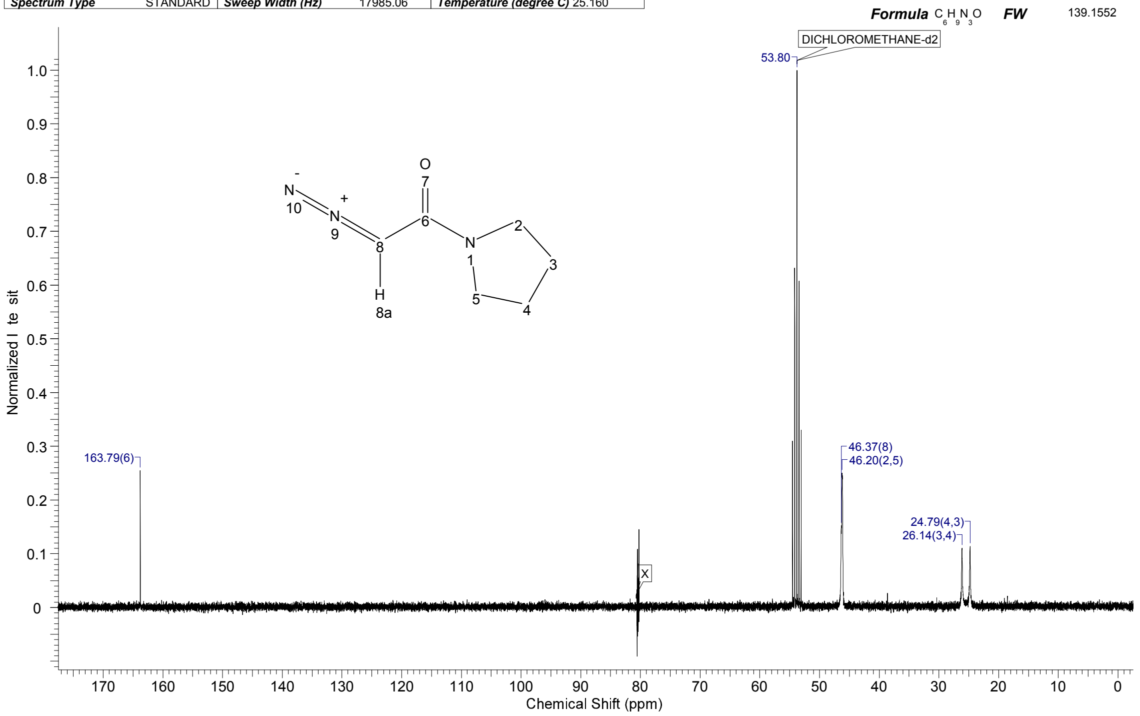

Acquisition Time (sec)	1.8219	***Comment***	C13DEPT135 CD2Cl2 D: {UIO\aasmuk} 27			***Frequency (MHz)***	75.48	***Nucleus***	13C
Number of Transients	1024	***Origin***	DPX300	***Original Points Count***	32768	***Points Count***	32768	***Pulse Sequence***	dept135
Receiver Gain	16384.00	***SW(cyclical) (Hz)***	17985.61	***Solvent***	DICHLOROMETHANE-d2			***Spectrum Offset (Hz)***	7570.8491
Spectrum Type	DEPT135	***Sweep Width (Hz)***	17985.06	***Temperature (degree C)***	25.160				

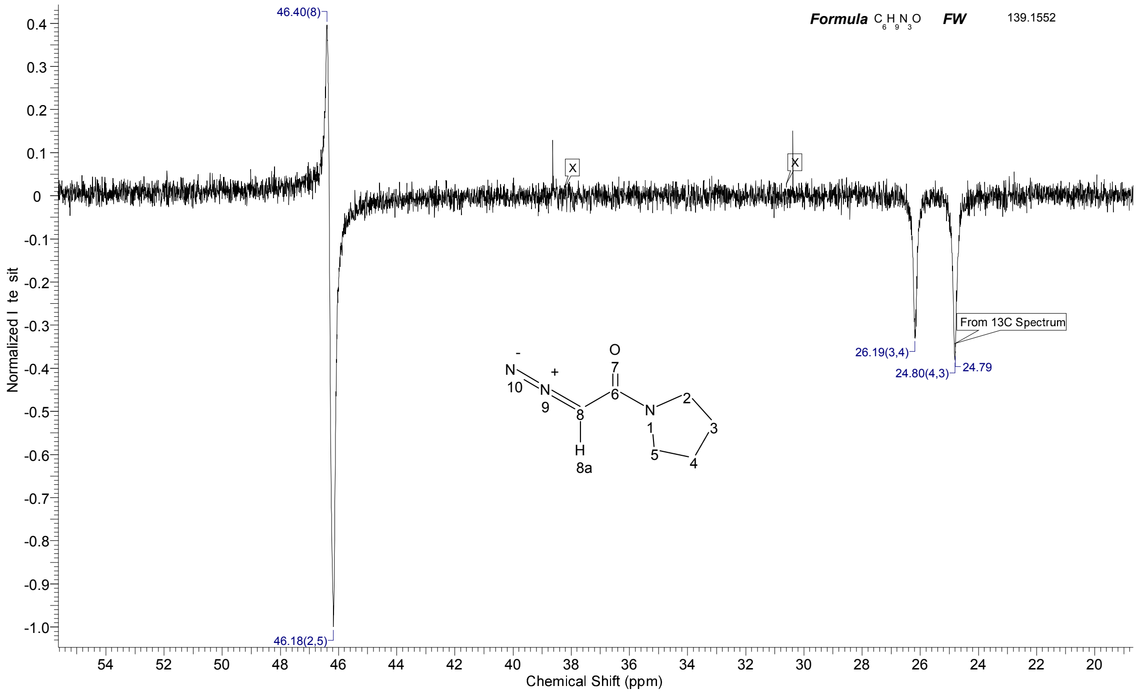

**Acquisition Time (sec)**	5.3084	**Comment**	5 mm QNP 1H/15N/13C/31P Z08011/0020	**Frequency (MHz)**	300.13	**Nucleus**	1H
**Number of Transients**	16	**Origin**	DPX300	**Original Points Count**	32768	**Points Count**	32768
**Pulse Sequence**	zg30	**Receiver Gain**	50.80	**SW(cyclical) (Hz)**	6172.84	**Solvent**	DICHLOROMETHANE-d2
**Spectrum Offset (Hz)**	1842.9987	**Spectrum Type**	STANDARD	**Sweep Width (Hz)**	6172.65	**Temperature (degree C)**	25.160

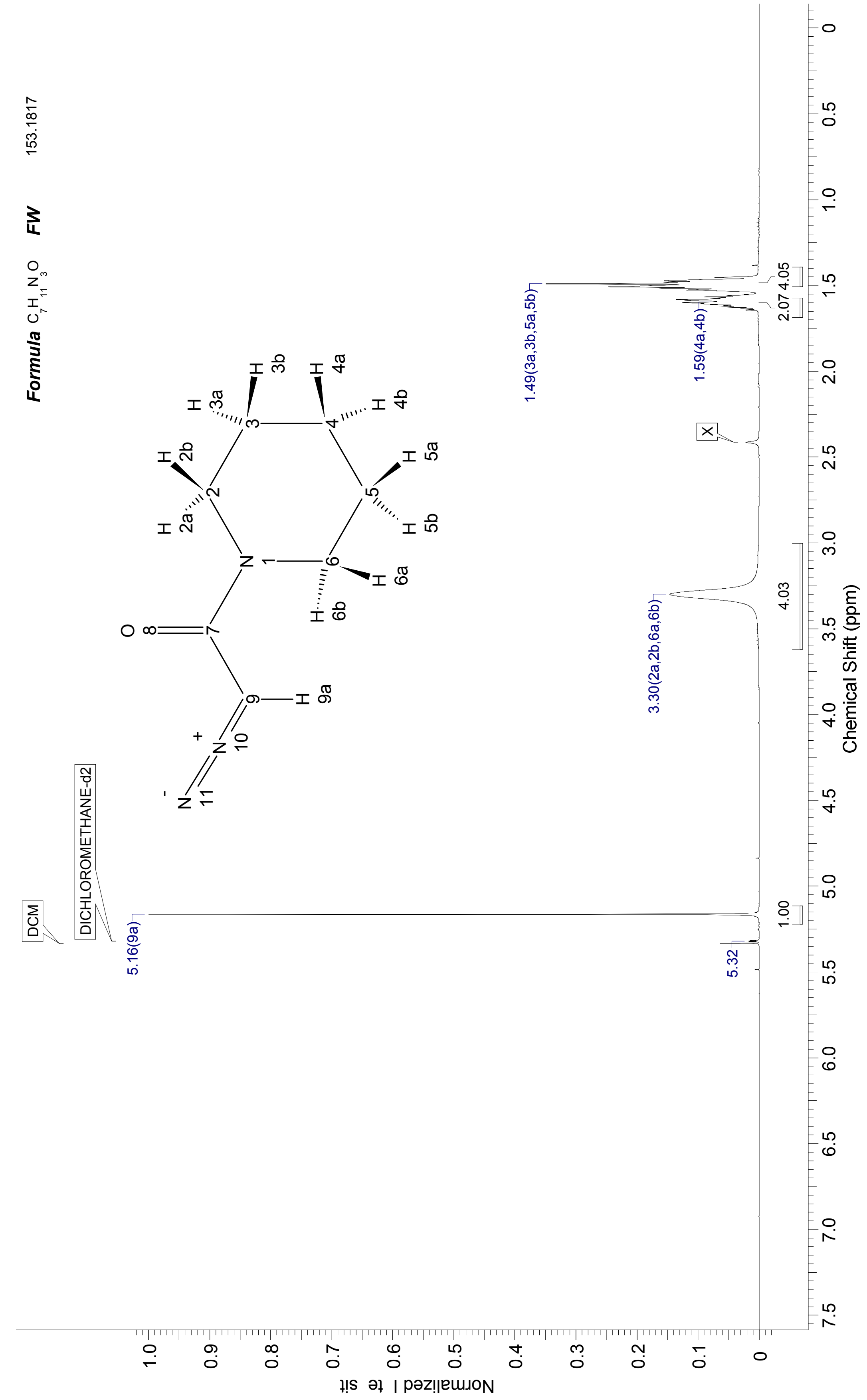

Acquisition Time (sec)	1.8219	***Comment***	C13CPD CD2Cl2 D: {UIO\aasmuk} 23			***Frequency (MHz)***	75.48	***Nucleus***	13C
Number of Transients	1024	***Origin***	DPX300	***Original Points Count***	32768	***Points Count***	32768	***Pulse Sequence***	zgpg30
Receiver Gain	11585.20	***SW(cyclical) (Hz)***	17985.61	***Solvent***	DICHLOROMETHANE-d2			***Spectrum Offset (Hz)***	7568.1714
Spectrum Type	STANDARD	***Sweep Width (Hz)***	17985.06	***Temperature (degree C)***	25.160				

Formula $C_7H_{11}N_3O$ ***FW*** 153.1817

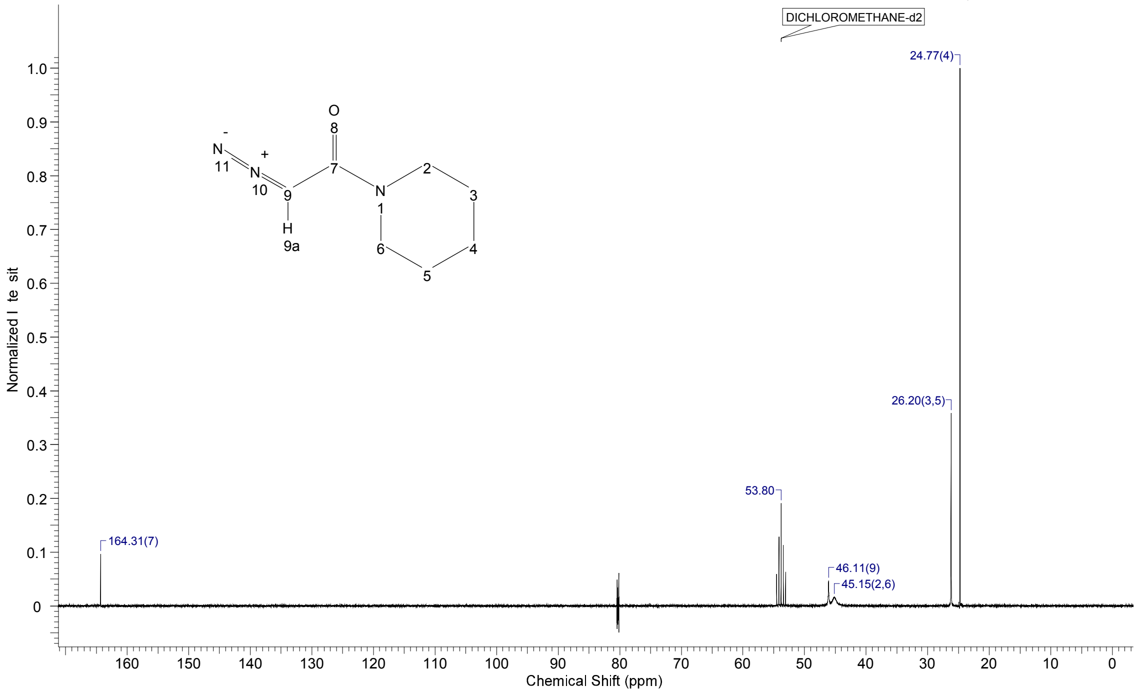

Acquisition Time (sec)	1.8219	***Comment***	C13DEPT135 CD2Cl2 D: {UIO\aasmuk} 23			***Frequency (MHz)***	75.48	***Nucleus***	13C
Number of Transients	1024	***Origin***	DPX300	***Original Points Count***	32768	***Points Count***	32768	***Pulse Sequence***	dept135
Receiver Gain	11585.20	***SW(cyclical) (Hz)***	17985.61	***Solvent***	DICHLOROMETHANE-d2			***Spectrum Offset (Hz)***	7568.2417
Spectrum Type	DEPT135	***Sweep Width (Hz)***	17985.06	***Temperature (degree C)***	25.160				

Formula $C_7H_{11}N_3O$ ***FW*** 153.1817

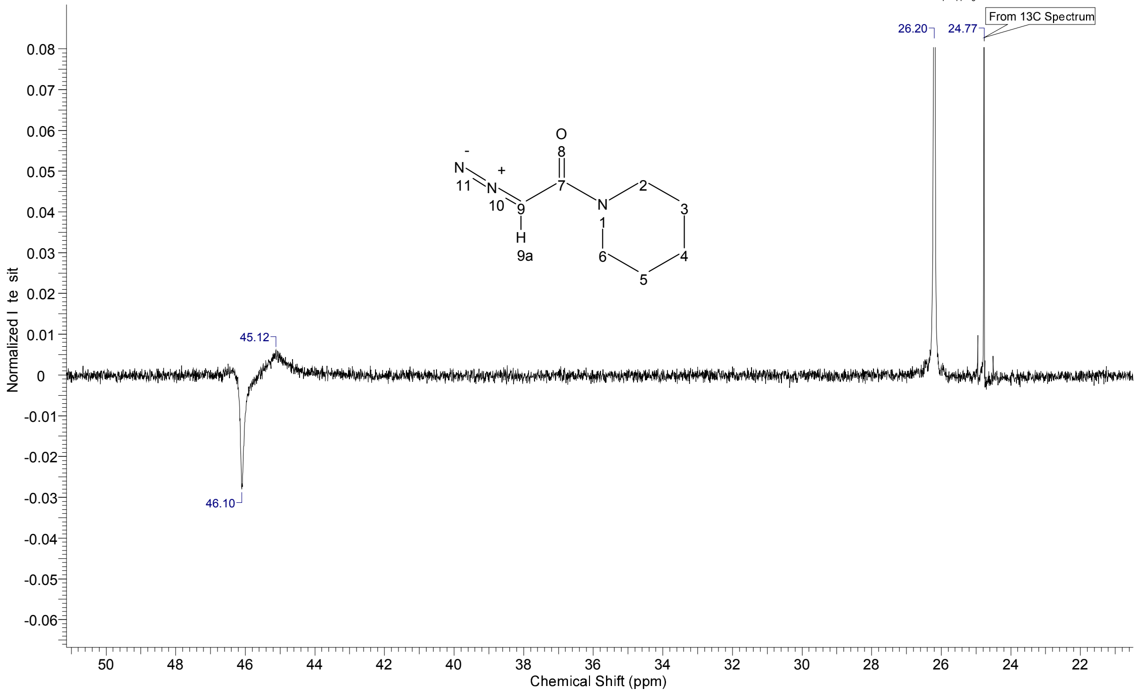

**Acquisition Time (sec)**	(0.3211, 0.0092)	**Comment**	5 mm QNP 1H/15N/13C/31P Z08011/0020			**Frequency (MHz)**	(300.13, 75.47)
**Nucleus**	(1H, 13C)	**Origin**	DPX300	**Original Points Count**	(512, 128)	**Points Count**	(512, 1024)
**Pulse Sequence**	hmqcgf	**Solvent**	CD2Cl2	**Spectrum Type**	HMQC	**Sweep Width (Hz)**	(1591.27, 13952.84)

Formula $C_7H_{11}N_3O$ **FW** 153.1817

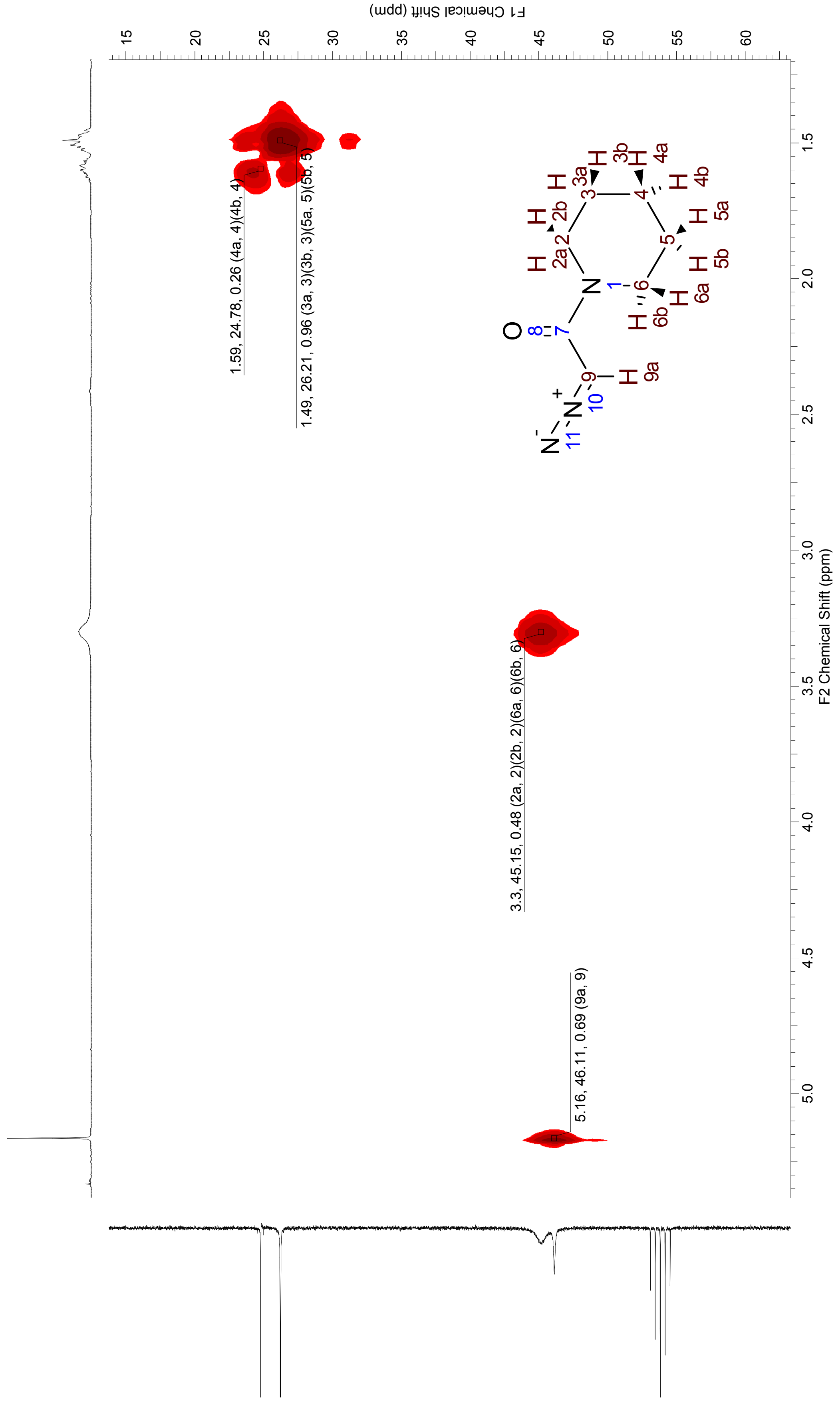

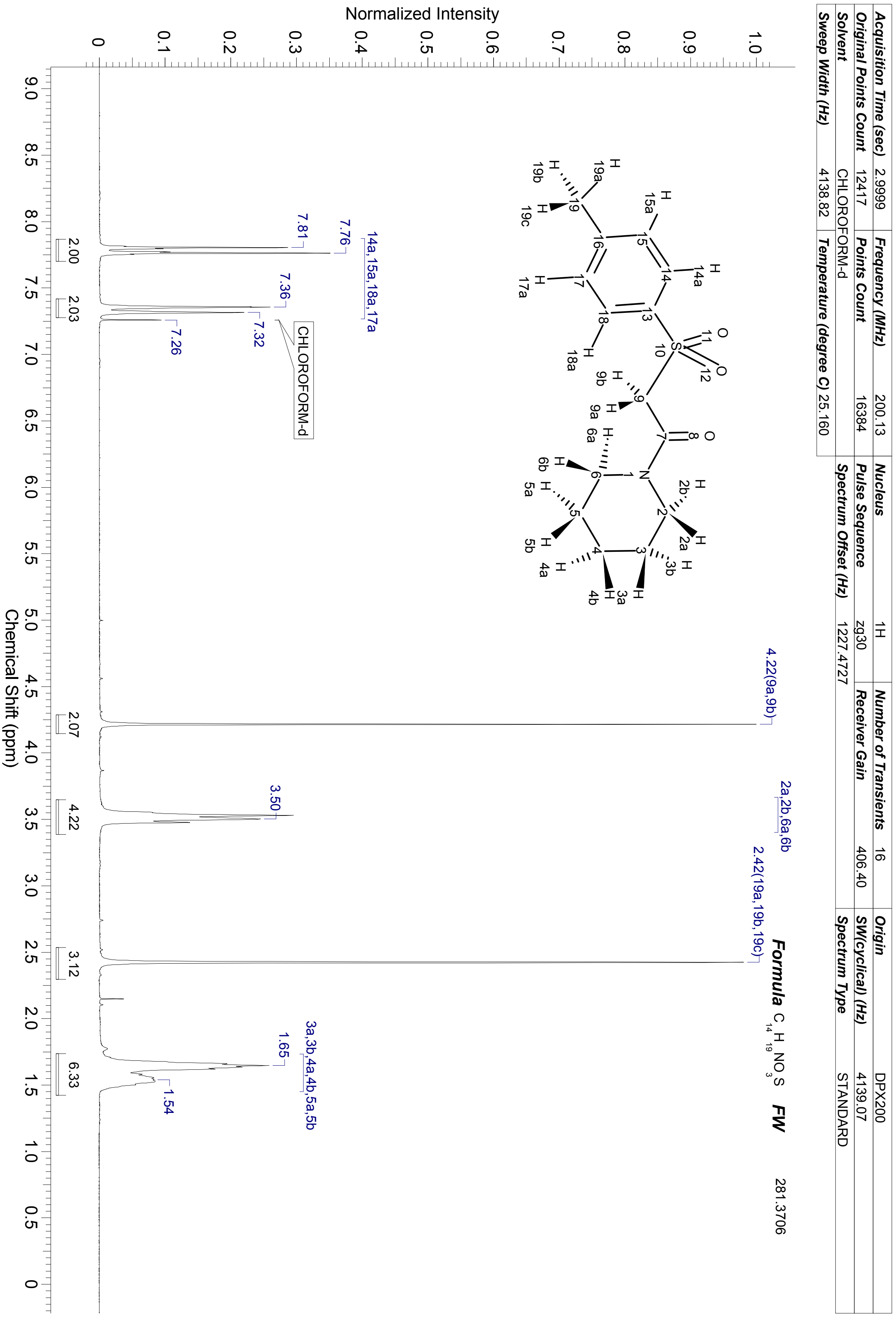
Acquisition Time (sec) 2.9999
Frequency (MHz) 200.13
Nucleus 1H
Number of Transients 16
Origin DPX200
Original Points Count 12417
Points Count 16384
Pulse Sequence zg30
Receiver Gain 406.40
SW(cyclical) (Hz) 4139.07
Solvent CHLOROFORM-d
Spectrum Offset (Hz) 1227.4727
Spectrum Type STANDARD
Sweep Width (Hz) 4138.82
Temperature (degree C) 25.160
Formula C14H19NO3S
FW 281.3706
Normalized Intensity
Chemical Shift (ppm)
4.22(9a,9b)
2.42(19a,19b,19c)
2a,2b,6a,6b
3.50
14a,15a,18a,17a
7.81
7.76
7.36
7.32
7.26
CHLOROFORM-d
3a,3b,4a,4b,5a,5b
1.65
1.54
2.00
2.03
2.07
4.22
3.12
6.33

**Acquisition Time (sec)**	1.8219	**Comment**	C13CPD CDCl3 D: {UIO\aasmuk} 24			**Frequency (MHz)**	75.48	**Nucleus**	13C
**Number of Transients**	1024	**Origin**	DPX300	**Original Points Count**	32768	**Points Count**	32768	**Pulse Sequence**	zgpg30
**Receiver Gain**	10321.30	**SW(cyclical) (Hz)**	17985.61	**Solvent**	CHLOROFORM-d			**Spectrum Offset (Hz)**	7539.7378
**Spectrum Type**	STANDARD			**Sweep Width (Hz)**	17985.06	**Temperature (degree C)**	25.160		

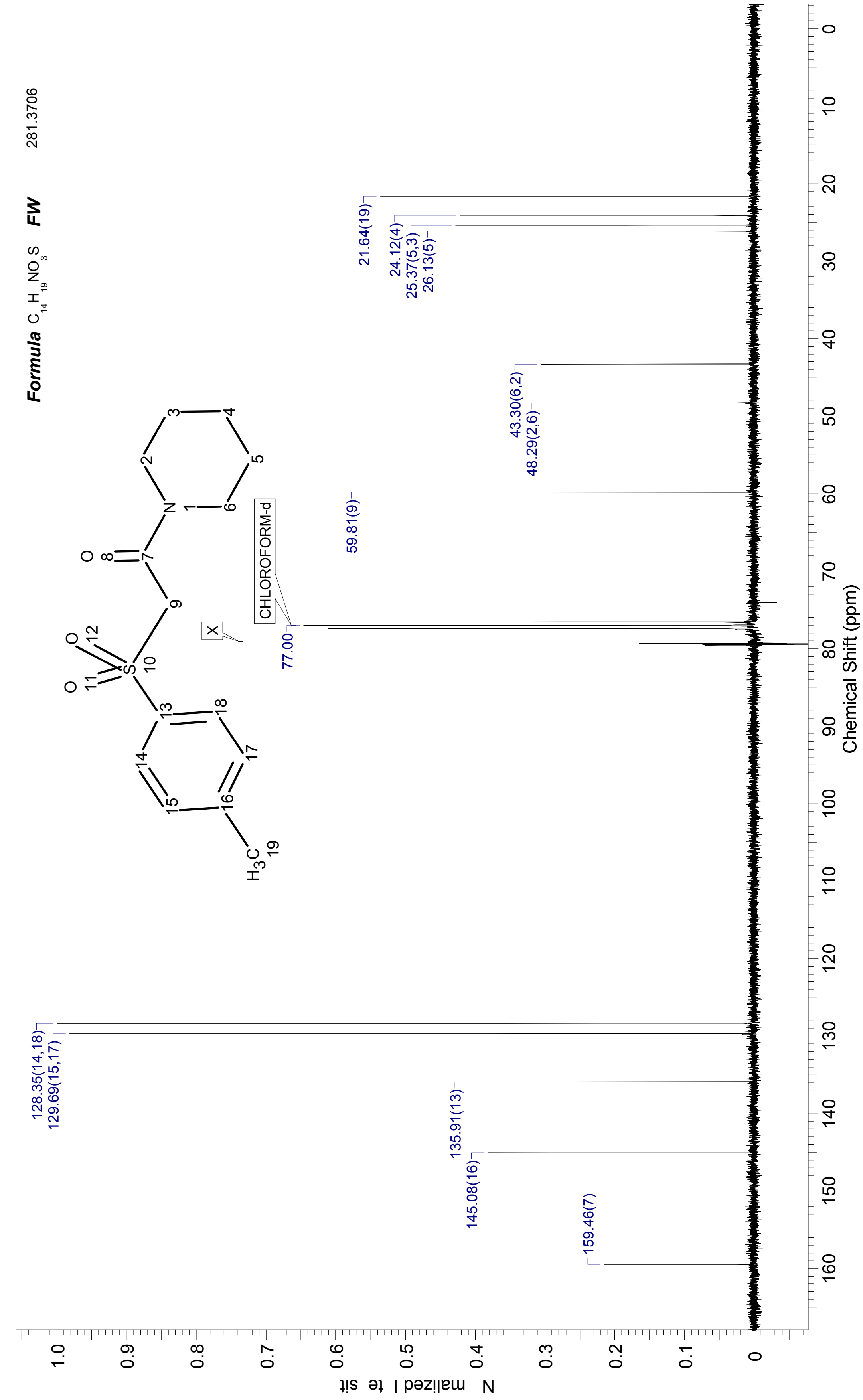

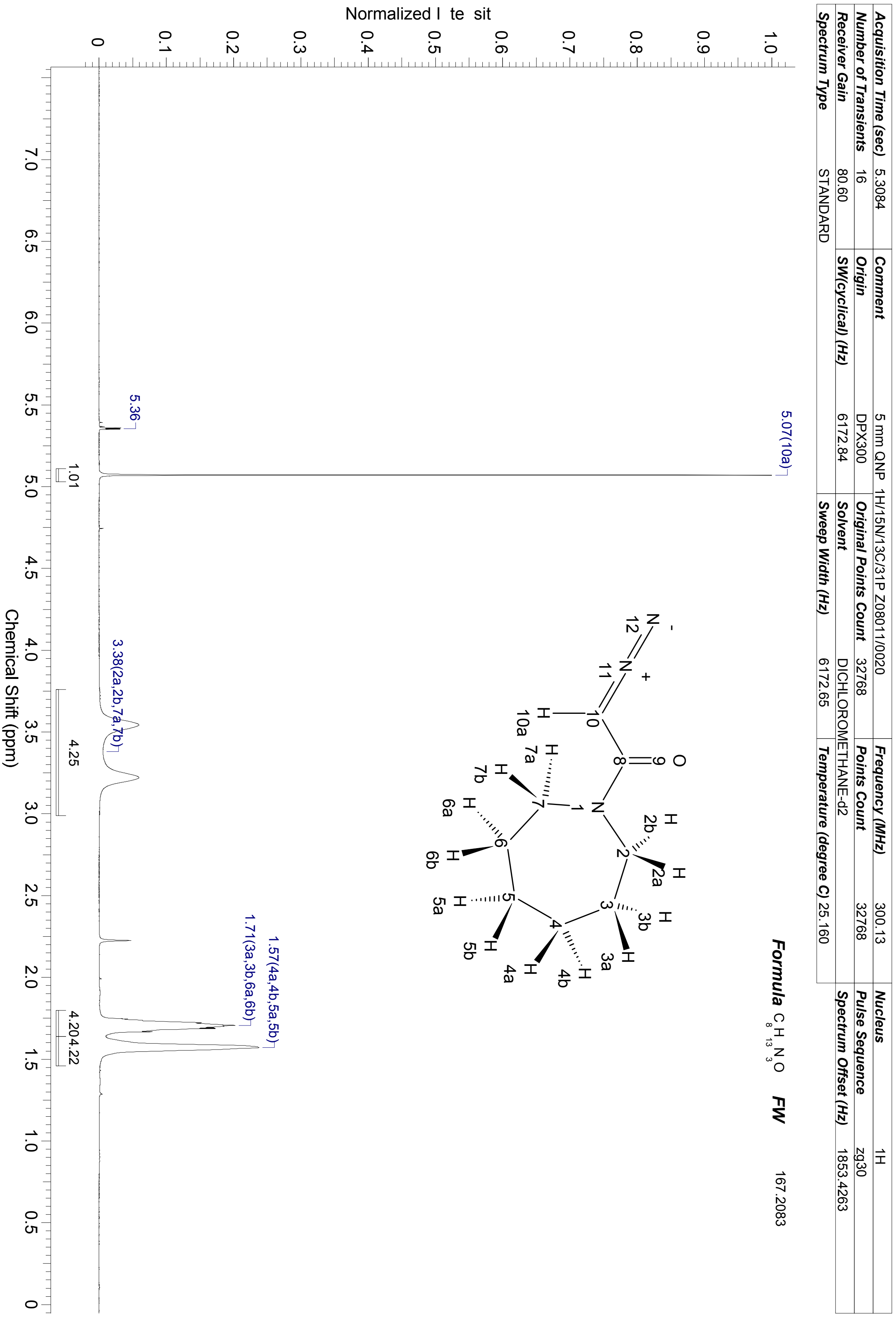
Acquisition Time (sec) 5.3084
Comment 5 mm QNP 1H/15N/13C/31P Z08011/0020
Frequency (MHz) 300.13
Nucleus 1H
Number of Transients 16
Origin DPX300
Original Points Count 32768
Points Count 32768
Pulse Sequence zg30
Receiver Gain 80.60
SW(cyclical) (Hz) 6172.84
Solvent DICHLOROMETHANE-d2
Spectrum Offset (Hz) 1853.4263
Spectrum Type STANDARD
Sweep Width (Hz) 6172.65
Temperature (degree C) 25.160
Formula C8H13N3O
FW 167.2083
Normalized Intensity
Chemical Shift (ppm)
5.07(10a)
5.36
3.38(2a,2b,7a,7b)
1.57(4a,4b,5a,5b)
1.71(3a,3b,6a,6b)
1.01
4.25
4.20
4.22

Acquisition Time (sec)	1.8219	***Comment***	C13CPD CD2Cl2 D: {UIO\aasmuk} 59			***Frequency (MHz)***	75.48	***Nucleus***	13C
Number of Transients	1024	***Origin***	DPX300	***Original Points Count***	32768	***Points Count***	32768	***Pulse Sequence***	zgpg30
Receiver Gain	10321.30	***SW(cyclical) (Hz)***	17985.61	***Solvent***	DICHLOROMETHANE-d2			***Spectrum Offset (Hz)***	7572.5620
Spectrum Type	STANDARD	***Sweep Width (Hz)***	17985.06	***Temperature (degree C)***	25.160				

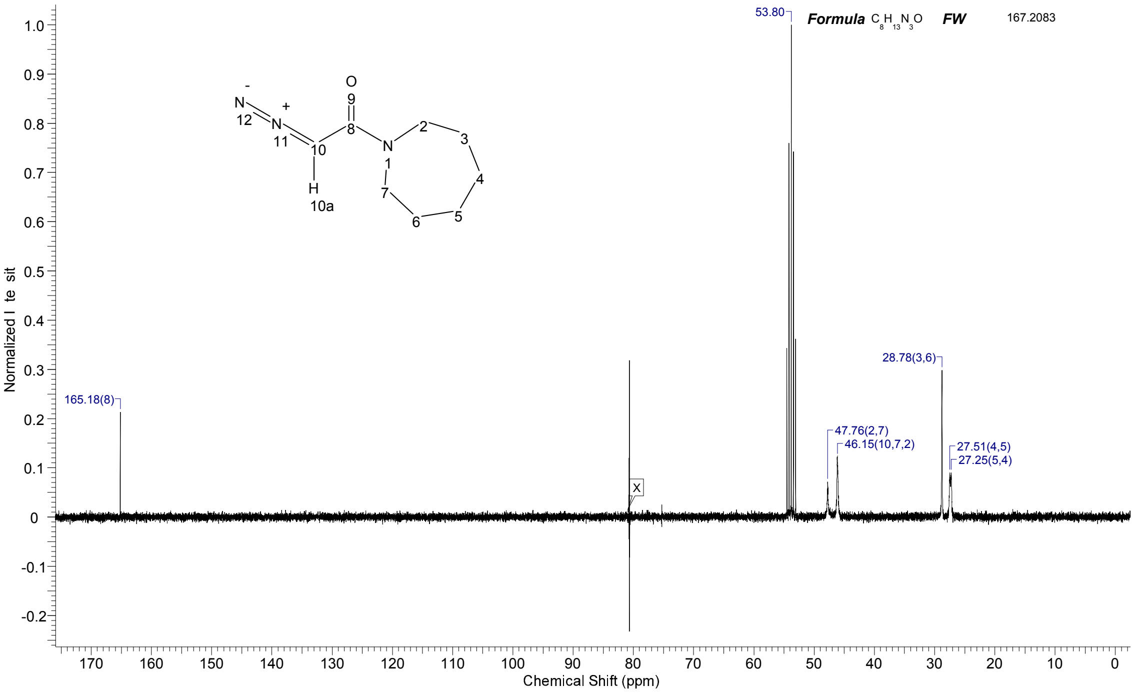

Acquisition Time (sec)	1.8219	***Comment***	C13DEPT135 CD2Cl2 D: {UIO\aasmuk} 28			***Frequency (MHz)***	75.48	***Nucleus***	13C
Number of Transients	1024	***Origin***	DPX300	***Original Points Count***	32768	***Points Count***	32768	***Pulse Sequence***	dept135
Receiver Gain	11585.20	***SW(cyclical) (Hz)***	17985.61	***Solvent***	DICHLOROMETHANE-d2			***Spectrum Offset (Hz)***	7572.8574
Spectrum Type	DEPT135	***Sweep Width (Hz)***	17985.06	***Temperature (degree C)***	25.160				

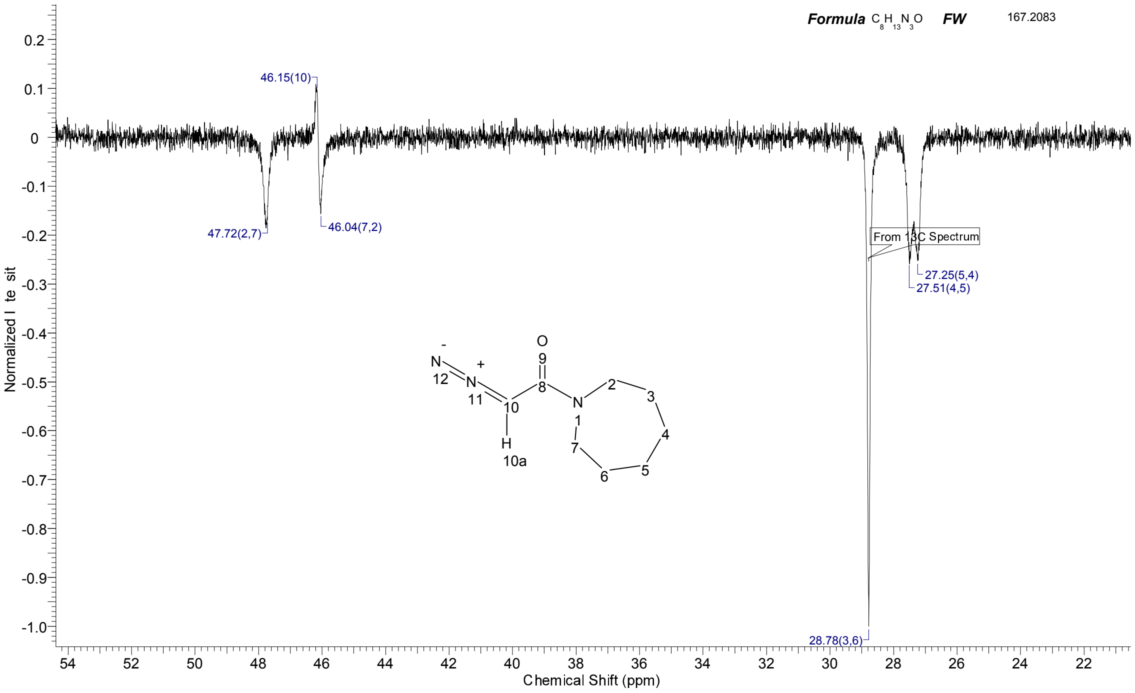

HMQC ($^{1}J_{^{13}C,^{1}H}$) – 1-(azepan-1-yl)-2-diazoethanone ***(3c)***

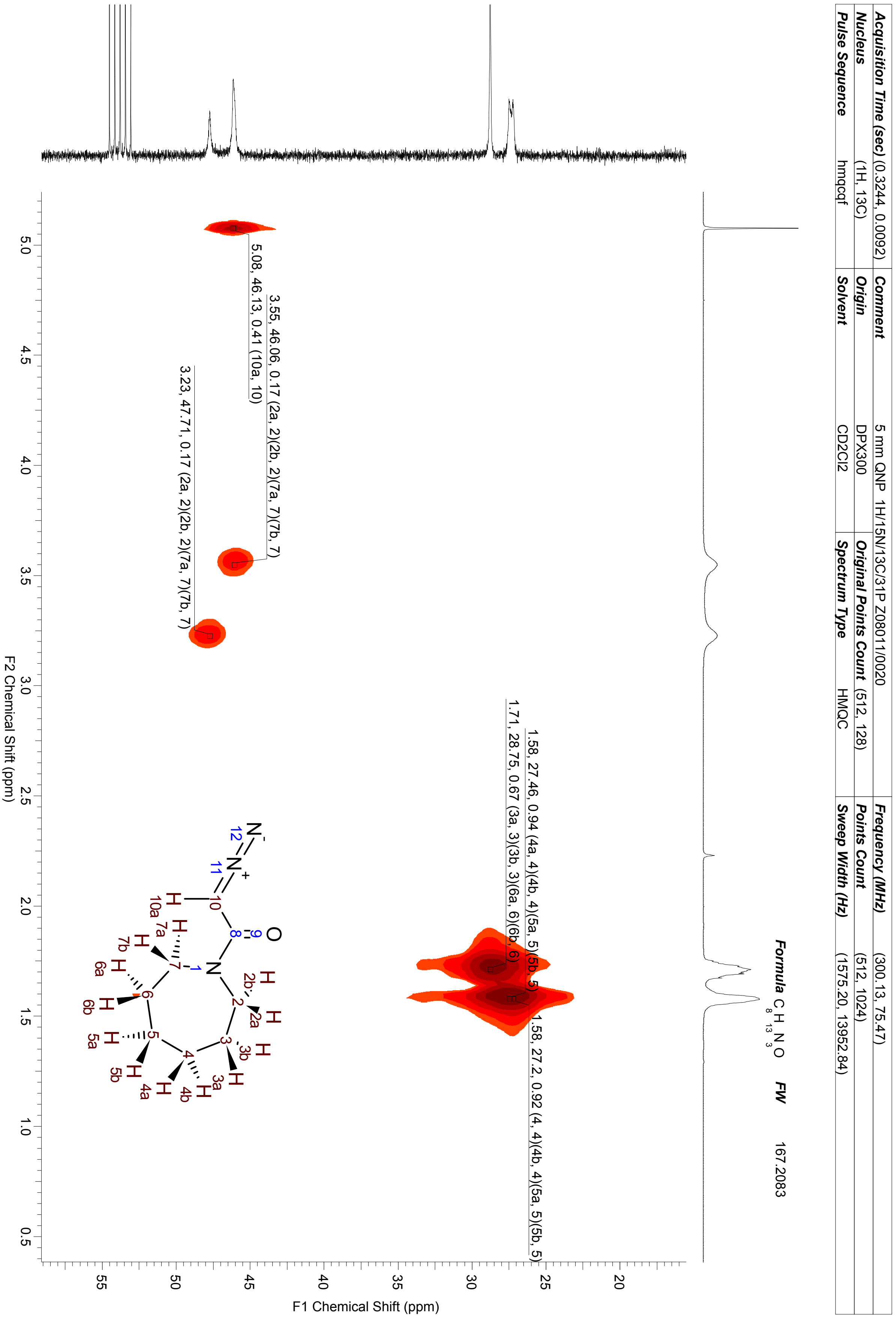

Acquisition Time (sec)	5.3084	Frequency (MHz)	300.13	Nucleus	1H	Origin	Bruker	Original Points Count	32768	Points Count	262144
Pulse Sequence	ZG30	Spectrum Offset (Hz)	1843.0216	Spectrum Type	STANDARD					Sweep Width (Hz)	6172.84

Formula $C_{12}H_{19}N_{3}O_{3}$ **FW** 253.2976

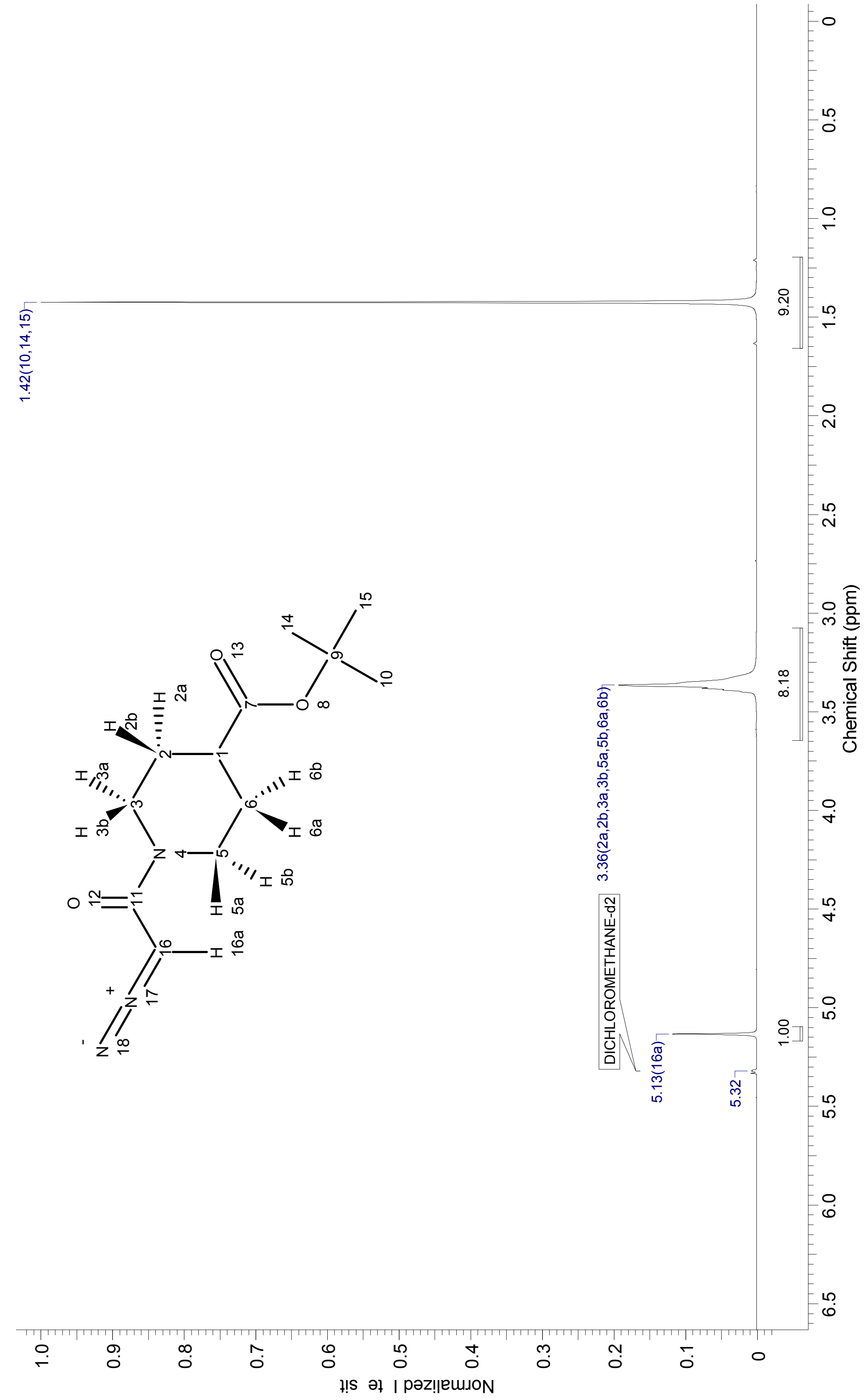

^{13}C-NMR & DEPT-135 – *tert-butyl 4-(2-diazoacetyl)piperazine-1-carboxylate* ***(3d)***

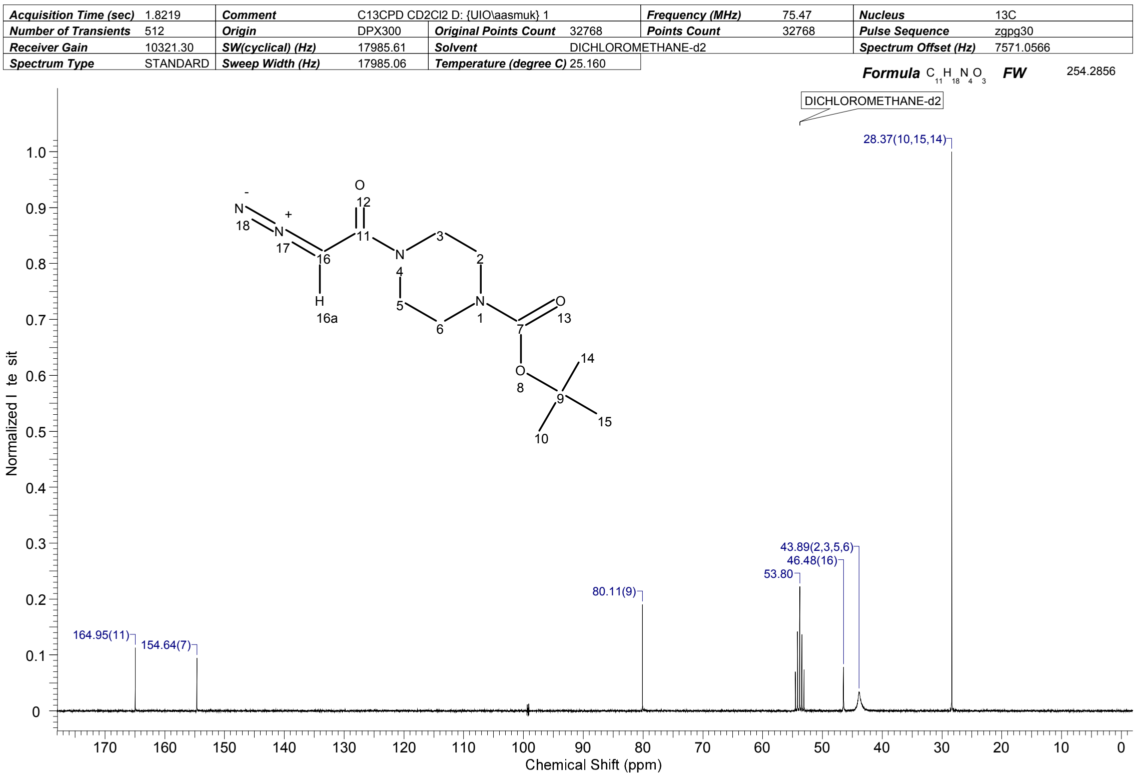

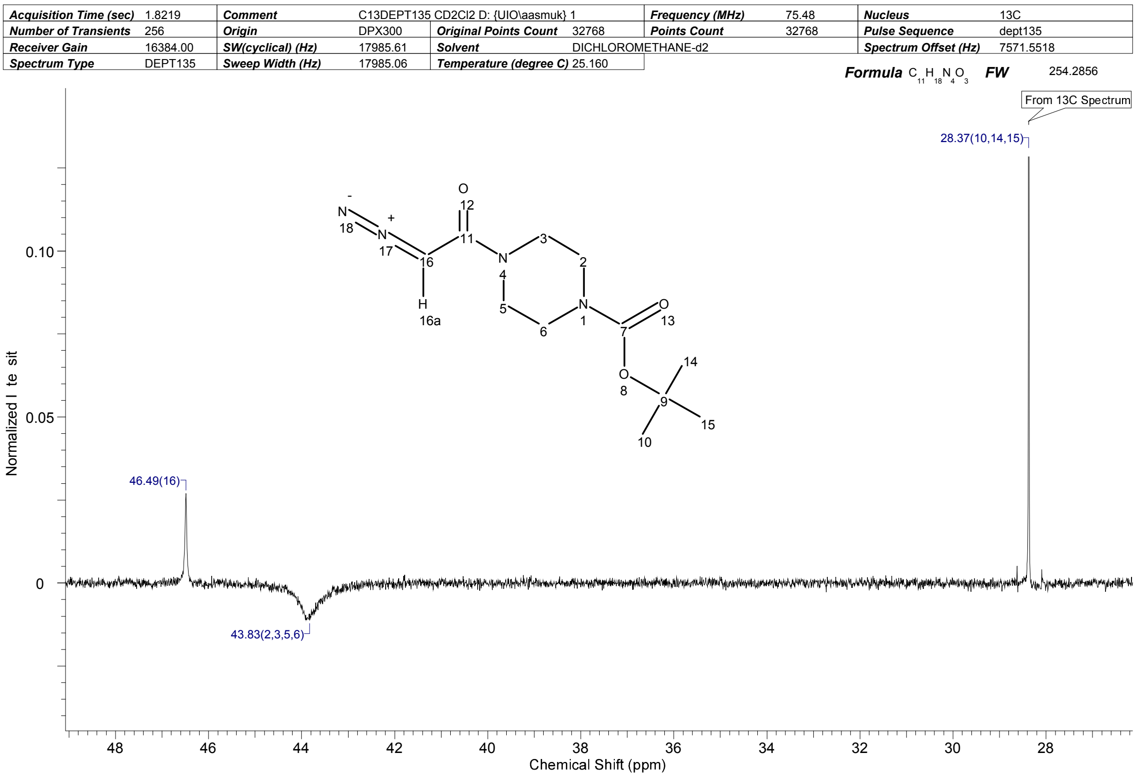

**Acquisition Time (sec)**	5.3084	**Comment**	5 mm QNP 1H/15N/13C/31P Z08011/0020			**Frequency (MHz)**	300.13	**Nucleus**	1H
**Number of Transients**	16	**Origin**	DPX300	**Original Points Count**	32768	**Points Count**	32768	**Pulse Sequence**	zg30
**Receiver Gain**	143.70	**SW(cyclical) (Hz)**	6172.84	**Solvent**	DICHLOROMETHANE-d2			**Spectrum Offset (Hz)**	1842.8103
**Spectrum Type**	STANDARD			**Sweep Width (Hz)**	6172.65	**Temperature (degree C)**	26.160		

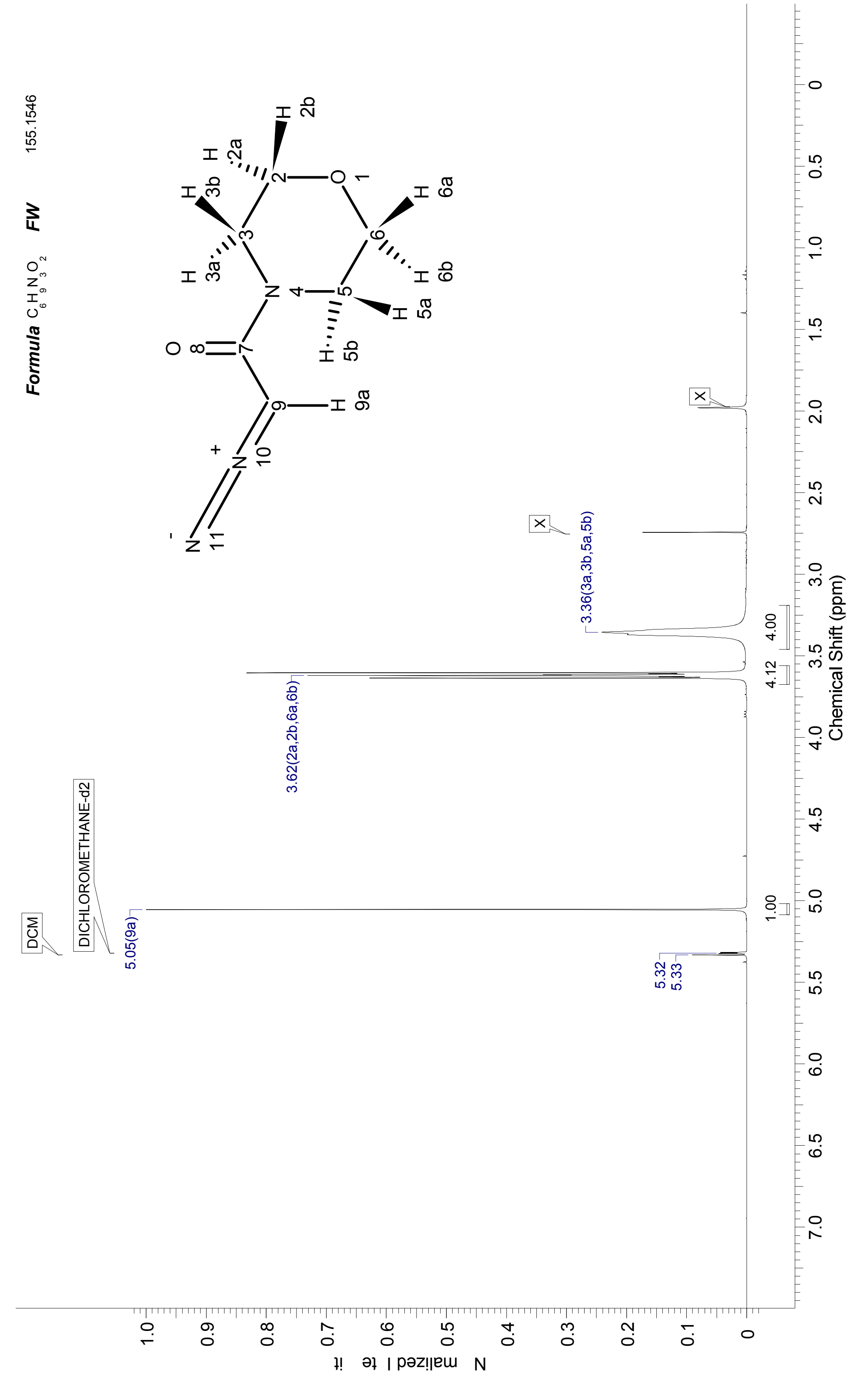

Acquisition Time (sec)	1.8219	***Comment***	C13CPD CD2Cl2 D: {UIO\aasmuk} 19			***Frequency (MHz)***	75.48	***Nucleus***	13C
Number of Transients	1024	***Origin***	DPX300	***Original Points Count***	32768	***Points Count***	32768	***Pulse Sequence***	zgpg30
Receiver Gain	11585.20	***SW(cyclical) (Hz)***	17985.61	***Solvent***	DICHLOROMETHANE-d2			***Spectrum Offset (Hz)***	7572.0137
Spectrum Type	STANDARD	***Sweep Width (Hz)***	17985.06	***Temperature (degree C)***	25.160				

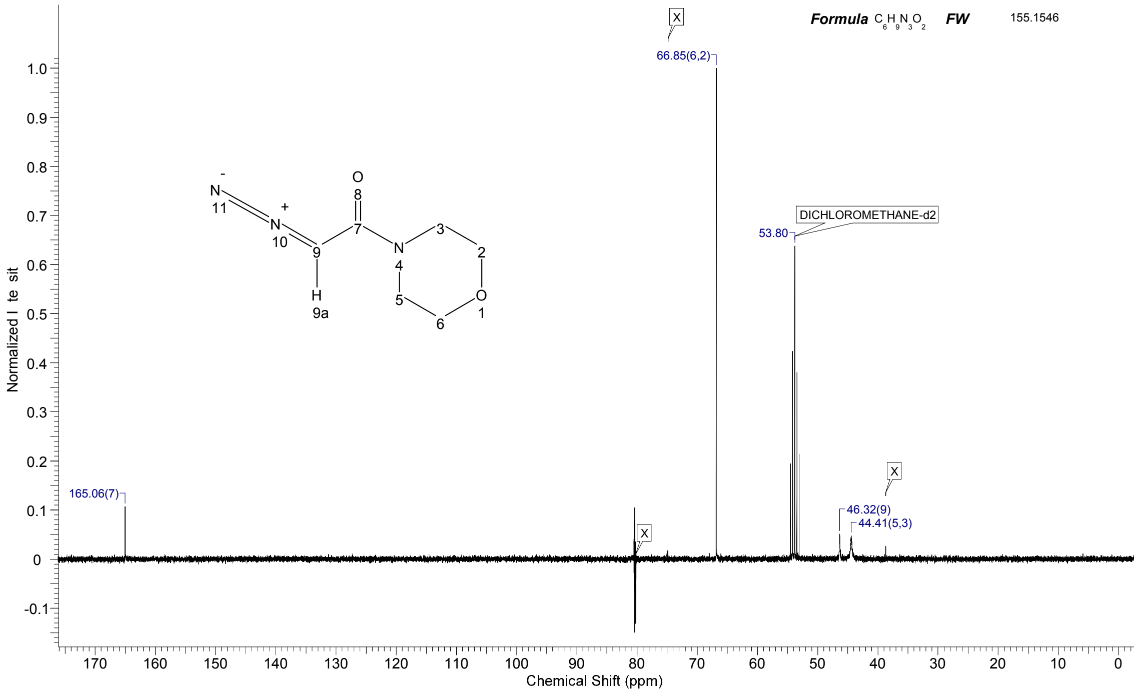

Acquisition Time (sec)	1.8219	***Comment***	C13DEPT135 CD2Cl2 D: {UIO\aasmuk} 19			***Frequency (MHz)***	75.48	***Nucleus***	13C
Number of Transients	1024	***Origin***	DPX300	***Original Points Count***	32768	***Points Count***	32768	***Pulse Sequence***	dept135
Receiver Gain	16384.00	***SW(cyclical) (Hz)***	17985.61	***Solvent***	DICHLOROMETHANE-d2			***Spectrum Offset (Hz)***	7571.7314
Spectrum Type	DEPT135	***Sweep Width (Hz)***	17985.06	***Temperature (degree C)***	25.160				

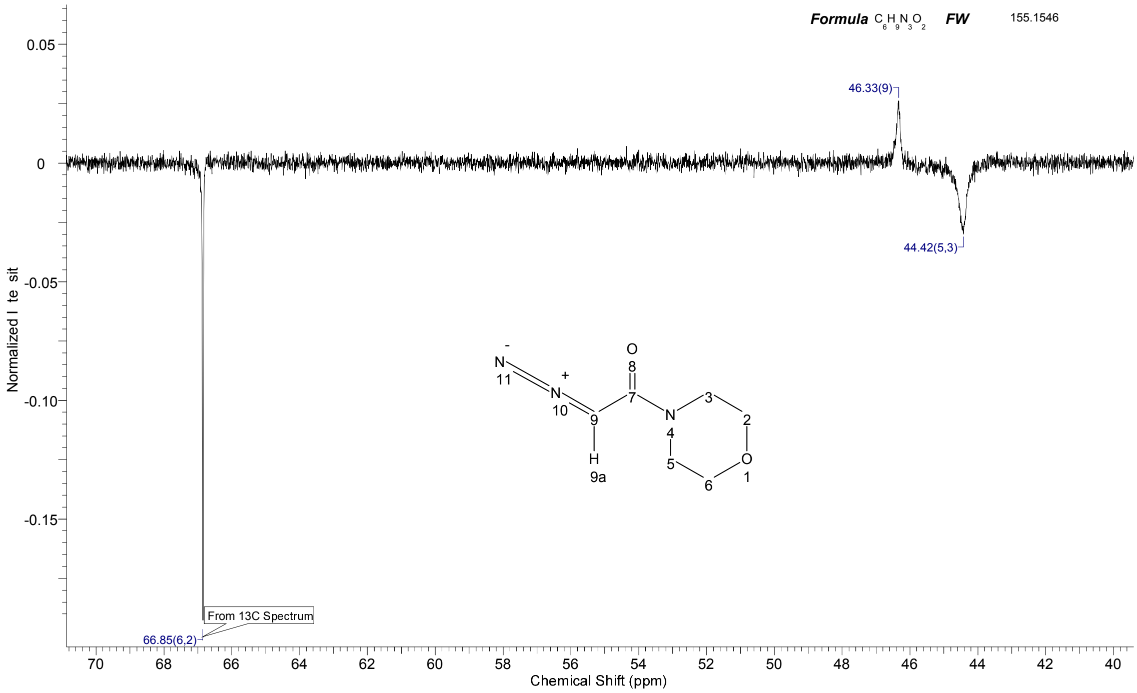

**Acquisition Time (sec)**	(0.4588, 0.0092)	**Comment**	5 mm QNP 1H/15N/13C/31P Z08011/0020			**Frequency (MHz)**	(300.13, 75.47)
**Nucleus**	(1H, 13C)	**Origin**	DPX300	**Original Points Count**	(512, 128)	**Points Count**	(512, 1024)
**Pulse Sequence**	hmqcgf	**Solvent**	CD2Cl2	**Spectrum Type**	HMQC	**Sweep Width (Hz)**	(1113.89, 13952.84)

Formula $C_6H_9N_3O_2$ ***FW*** 155.1546

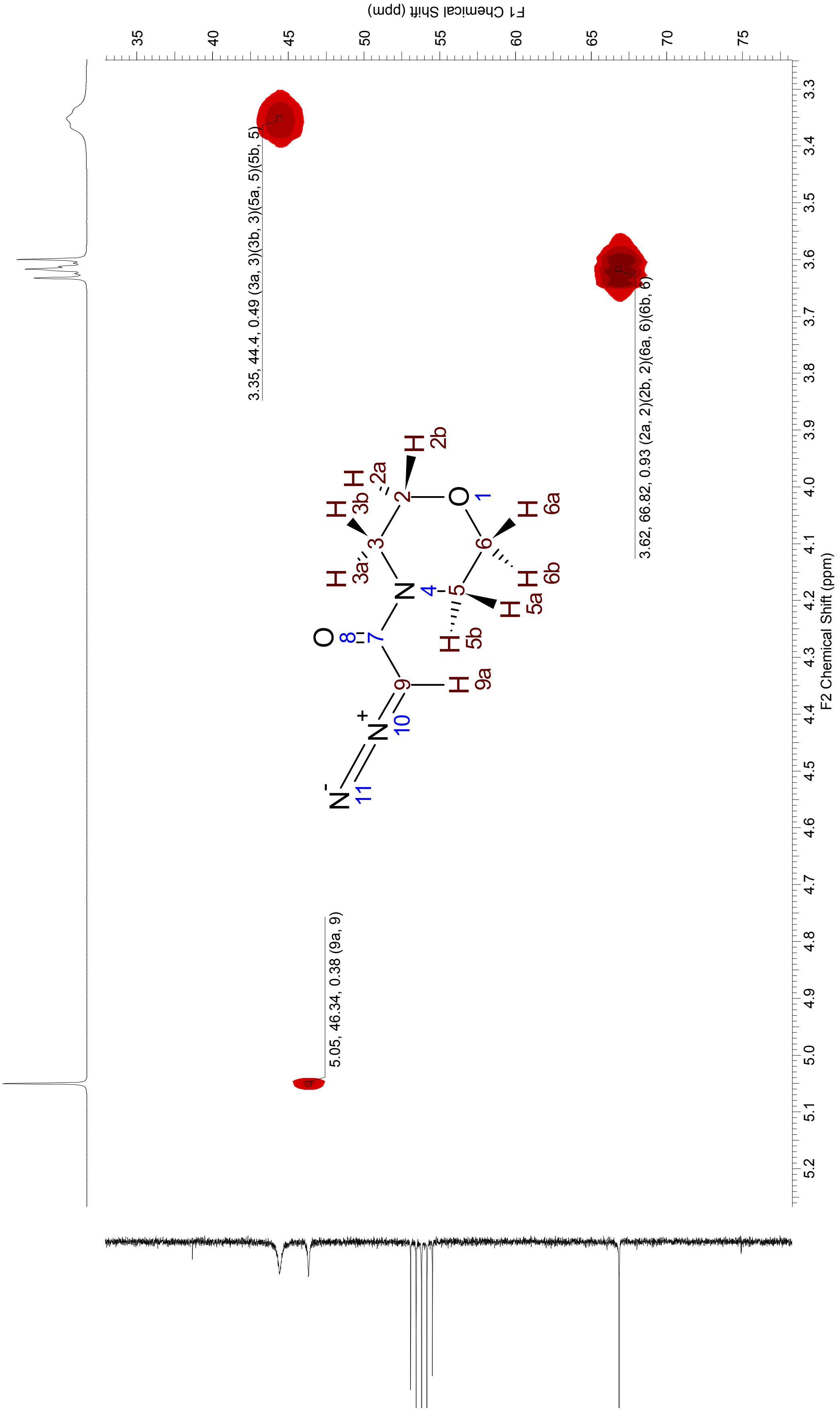

**Acquisition Time (sec)**	5.3084	**Comment**	PROTON CD2Cl2 D:\{UIO\aasmuk} 1			**Frequency (MHz)**	300.13	**Nucleus**	1H
**Number of Transients**	16	**Origin**	DPX300	**Original Points Count**	32768	**Points Count**	32768	**Pulse Sequence**	zg30
**Receiver Gain**	1448.20	**SW(cyclical) (Hz)**	6172.84	**Solvent**	DICHLOROMETHANE-d2			**Spectrum Offset (Hz)**	1842.8202
**Spectrum Type**	STANDARD			**Sweep Width (Hz)**	6172.65	**Temperature (degree C)**	25.160		

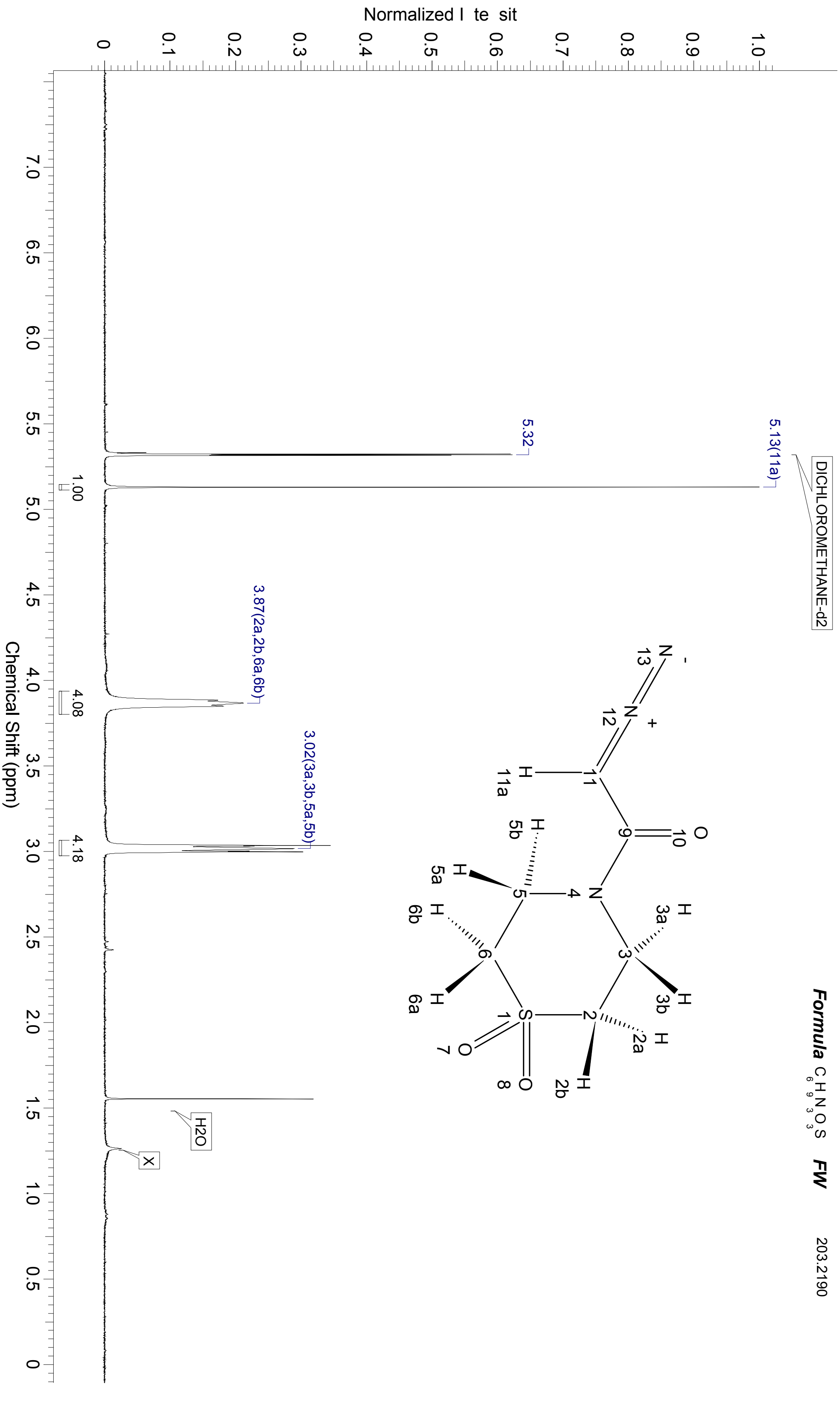

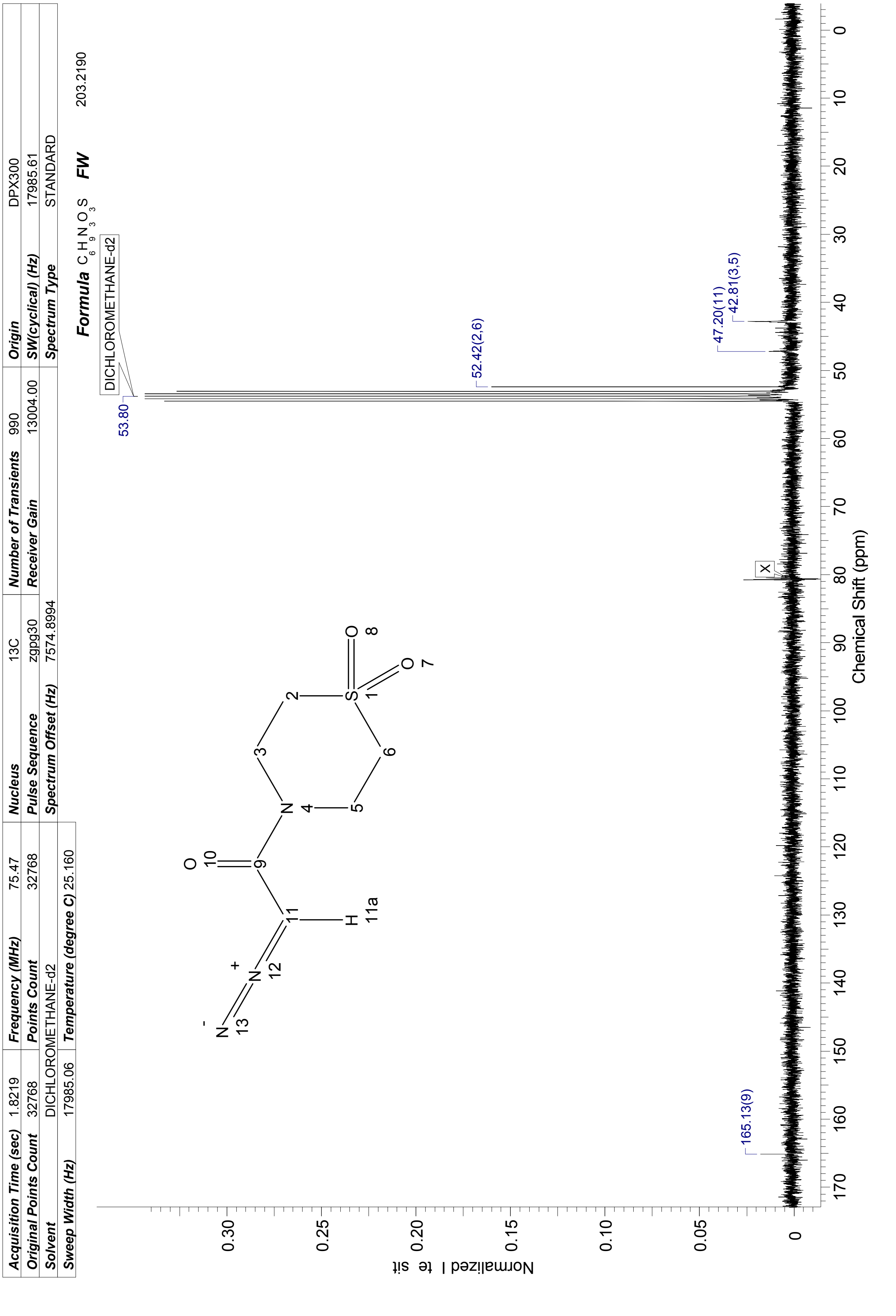
Acquisition Time (sec) 1.8219
Frequency (MHz) 75.47
Nucleus 13C
Number of Transients 990
Origin DPX300
Original Points Count 32768
Points Count 32768
Pulse Sequence zgpg30
Receiver Gain 13004.00
SW(cyclical) (Hz) 17985.61
Solvent DICHLOROMETHANE-d2
Spectrum Offset (Hz) 7574.8994
Spectrum Type STANDARD
Sweep Width (Hz) 17985.06
Temperature (degree C) 25.160
Formula C6 H9 N3 O3 S
FW 203.2190
DICHLOROMETHANE-d2
53.80
52.42(2,6)
47.20(11)
42.81(3,5)
165.13(9)
X
Chemical Shift (ppm)
Normalized Intensity

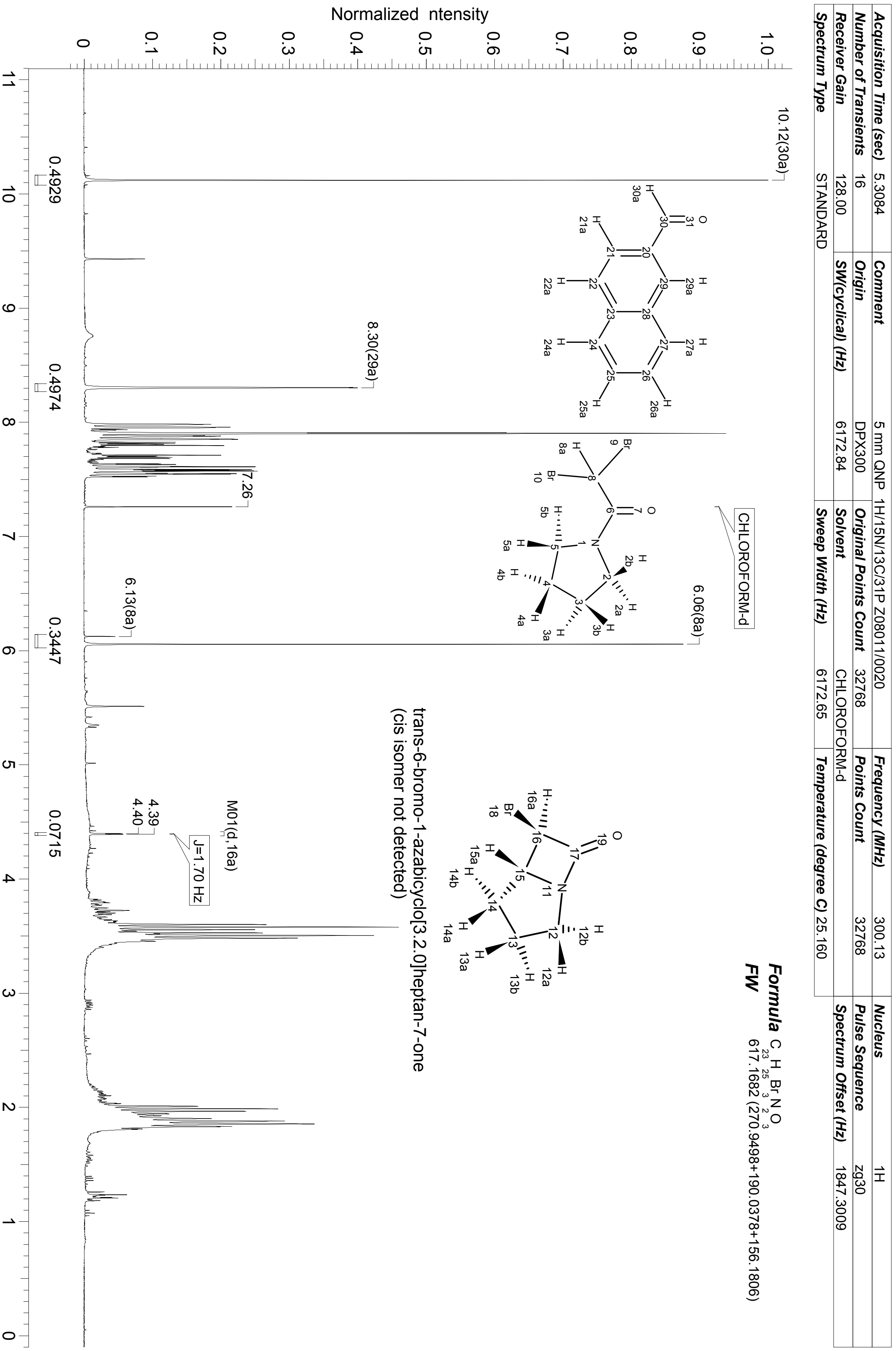
Acquisition Time (sec) 5.3084
Comment 5 mm QNP 1H/15N/13C/31P Z08011/0020
Frequency (MHz) 300.13
Nucleus 1H
Number of Transients 16
Origin DPX300
Original Points Count 32768
Points Count 32768
Pulse Sequence zg30
Receiver Gain 128.00
SW(cyclical) (Hz) 6172.84
Solvent CHLOROFORM-d
Spectrum Offset (Hz) 1847.3009
Spectrum Type STANDARD
Sweep Width (Hz) 6172.65
Temperature (degree C) 25.160
Formula C23 H25 Br3 N2 O3
FW 617.1682 (270.9498+190.0378+156.1806)
trans-6-bromo-1-azabicyclo[3.2.0]heptan-7-one
(cis isomer not detected)
CHLOROFORM-d
10.12(30a)
8.30(29a)
7.26
6.13(8a)
6.06(8a)
4.39
4.40
M01(d,16a)
J=1.70 Hz
0.4929
0.4974
0.3447
0.0715
Normalized Intensity
Chemical Shift (ppm)

Acquisition Time (sec)	5.3084	Comment	PROTON CDCl3 D: {UIO\aasmuk} 2			Frequency (MHz)	300.13	Nucleus	1H
Number of Transients	16	Origin	DPX300	Original Points Count	32768	Points Count	32768	Pulse Sequence	zg30
Receiver Gain	101.60	SW(cyclical) (Hz)	6172.84	Solvent	CHLOROFORM-d			Spectrum Offset (Hz)	1847.6909
Spectrum Type	STANDARD			Sweep Width (Hz)	6172.65	Temperature (degree C)	25.160		

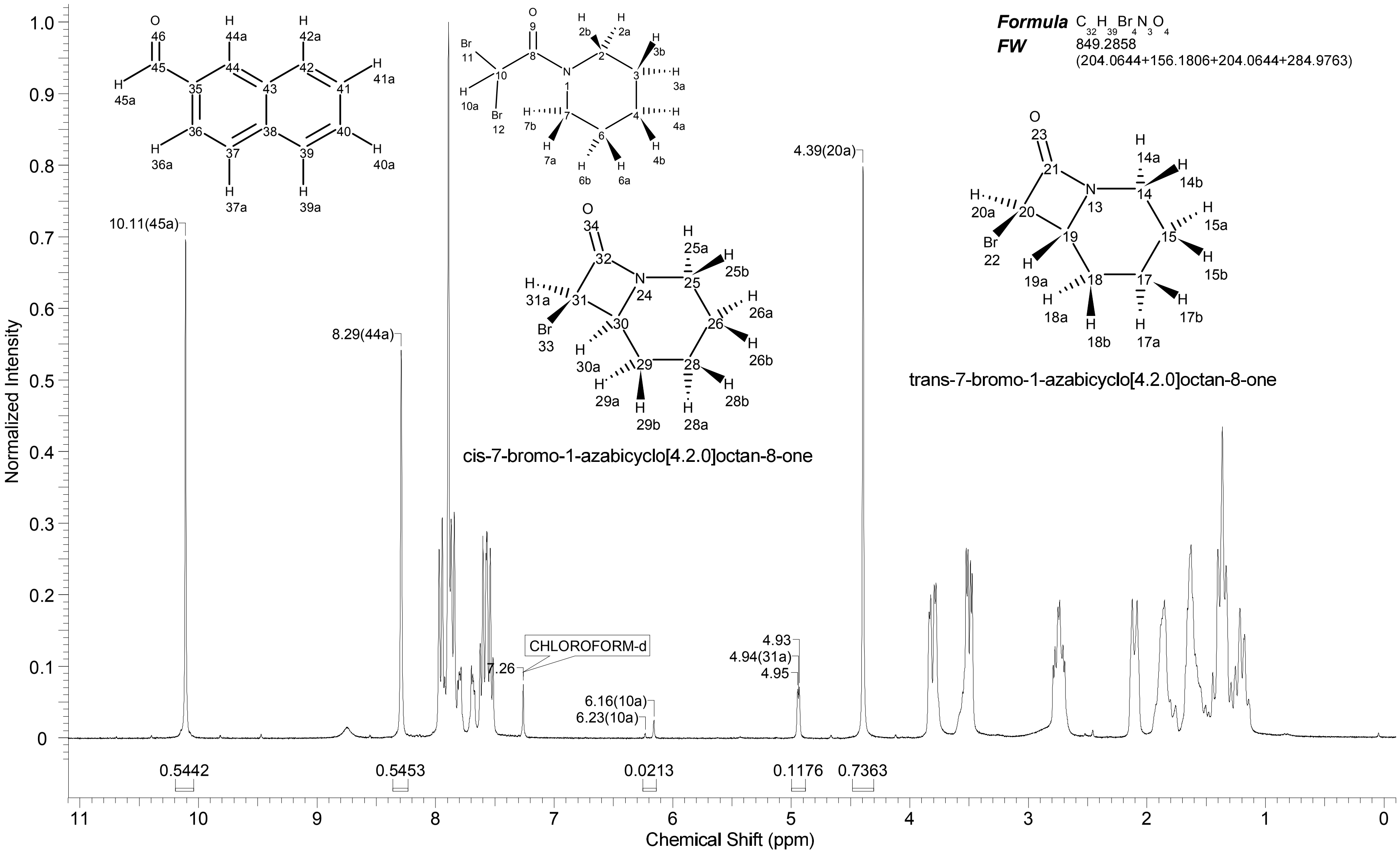

Acquisition Time (sec)	5.3084	***Comment***	5 mm QNP 1H/15N/13C/31P Z08011/0020	***Frequency (MHz)***	300.13	***Nucleus***	1H
Number of Transients	16	***Origin***	DPX300	***Original Points Count***	32768	***Points Count***	32768
Pulse Sequence	zg30	***Receiver Gain***	101.60	***SW(cyclical) (Hz)***	6172.84	***Solvent***	CHLOROFORM-d
Spectrum Offset (Hz)	1847.3009	***Spectrum Type***	STANDARD	***Sweep Width (Hz)***	6172.65	***Temperature (degree C)***	25.160

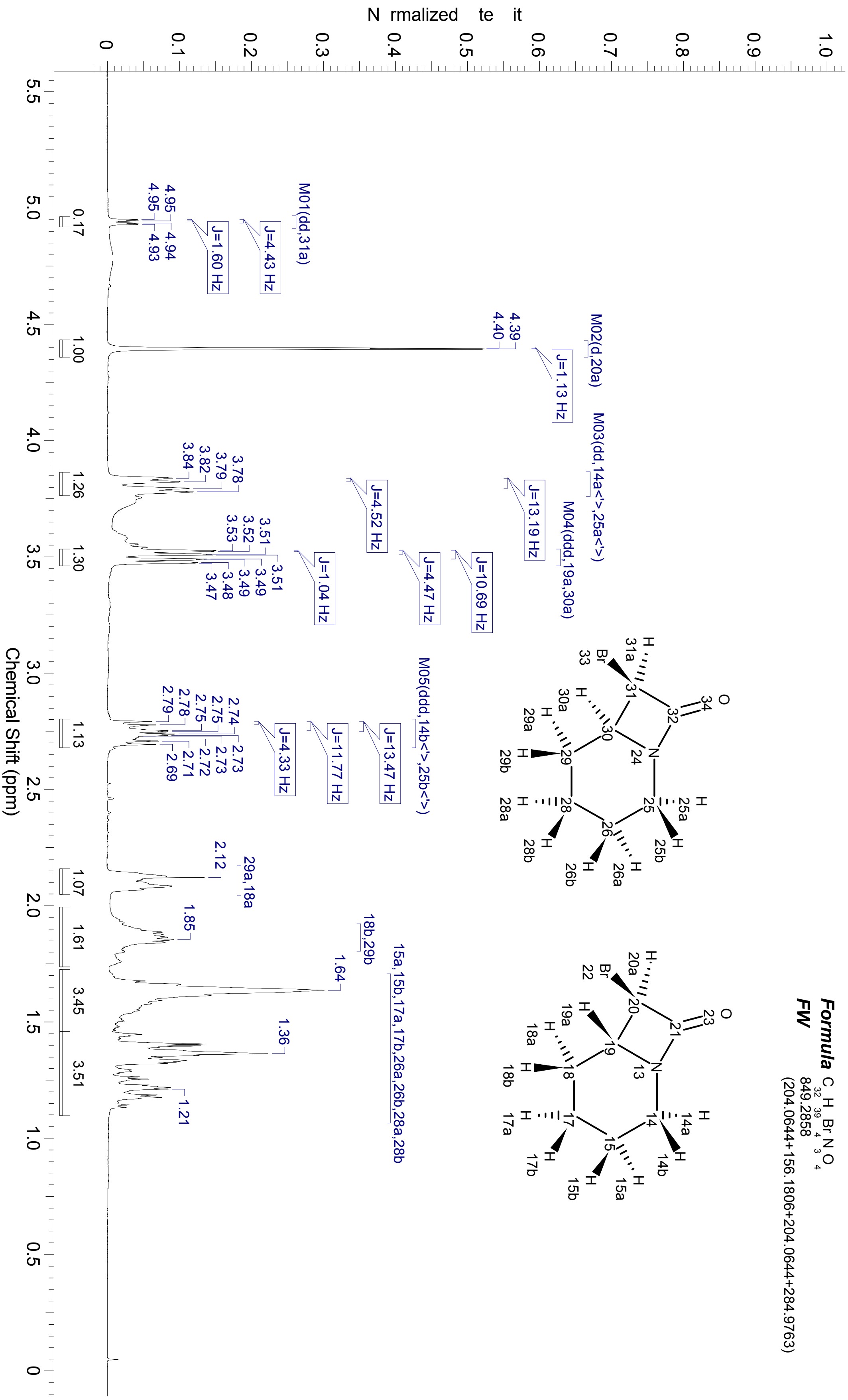

Acquisition Time (sec)	5.3084	***Comment***	5 mm QNP 1H/15N/13C/31P Z08011/0020			***Frequency (MHz)***	300.13	***Nucleus***	1H
Number of Transients	16	***Origin***	DPX300	***Original Points Count***	32768	***Points Count***	32768	***Pulse Sequence***	zg30
Receiver Gain	114.00	***SW(cyclical) (Hz)***	6172.84	***Solvent***	CHLOROFORM-d			***Spectrum Offset (Hz)***	1847.3009
Spectrum Type	STANDARD			***Sweep Width (Hz)***	6172.65	***Temperature (degree C)***	25.160		

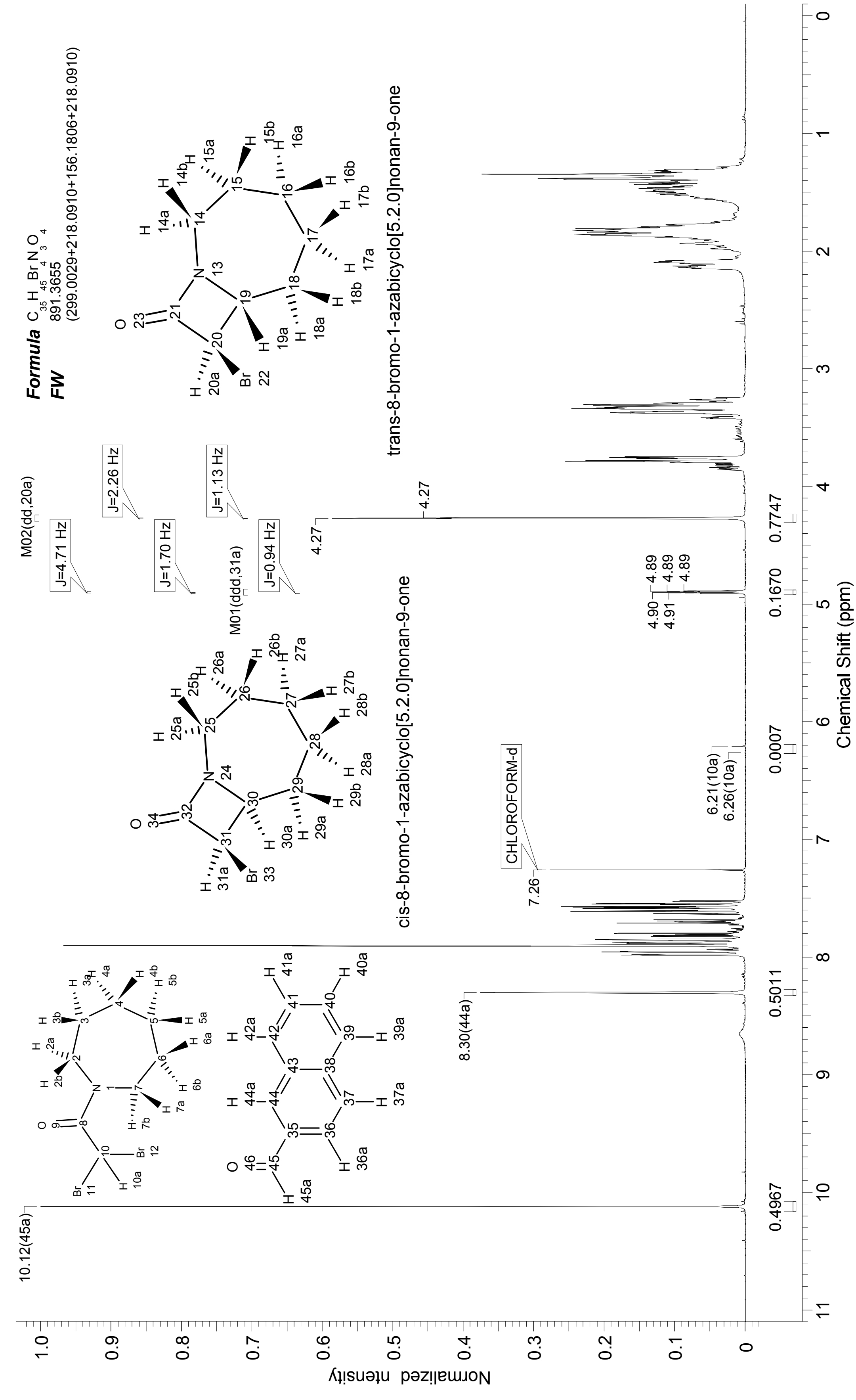

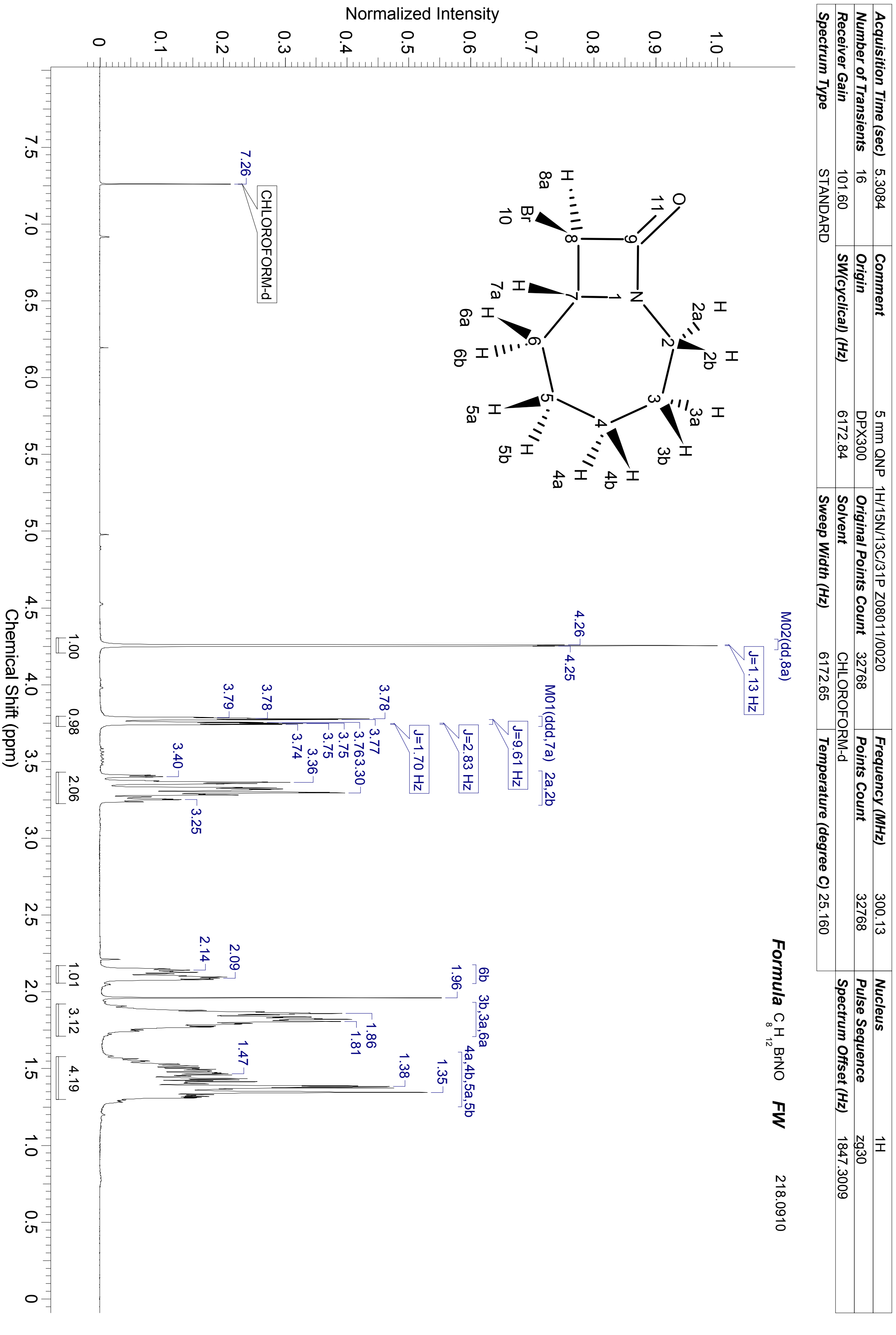
Acquisition Time (sec) 5.3084
Comment 5 mm QNP 1H/15N/13C/31P Z08011/0020
Frequency (MHz) 300.13
Nucleus 1H
Number of Transients 16
Origin DPX300
Original Points Count 32768
Points Count 32768
Pulse Sequence zg30
Receiver Gain 101.60
SW(cyclical) (Hz) 6172.84
Solvent CHLOROFORM-d
Spectrum Offset (Hz) 1847.3009
Spectrum Type STANDARD
Sweep Width (Hz) 6172.65
Temperature (degree C) 25.160
Formula C8H12BrNO
FW 218.0910
Normalized Intensity
Chemical Shift (ppm)
CHLOROFORM-d
7.26
M02(dd,8a)
J=1.13 Hz
4.26
4.25
M01(ddd,7a)
J=9.61 Hz
J=2.83 Hz
J=1.70 Hz
2a,2b
3.79
3.78
3.78
3.77
3.76
3.75
3.75
3.74
3.40
3.36
3.30
3.25
6b
3b,3a,6a
4a,4b,5a,5b
2.14
2.09
1.96
1.86
1.81
1.47
1.38
1.35
1.00
0.98
2.06
1.01
3.12
4.19

Acquisition Time (sec)	1.8219	***Frequency (MHz)***	75.48	***Nucleus***	13C	***Origin***	Bruker	***Original Points Count***	32768	***Points Count***	65536
Pulse Sequence	ZGPG30	***Spectrum Offset (Hz)***	7536.3345			***Spectrum Type***	STANDARD			***Sweep Width (Hz)***	17985.61

Formula $C_8H_{12}BrNO$ ***FW*** 218.0910

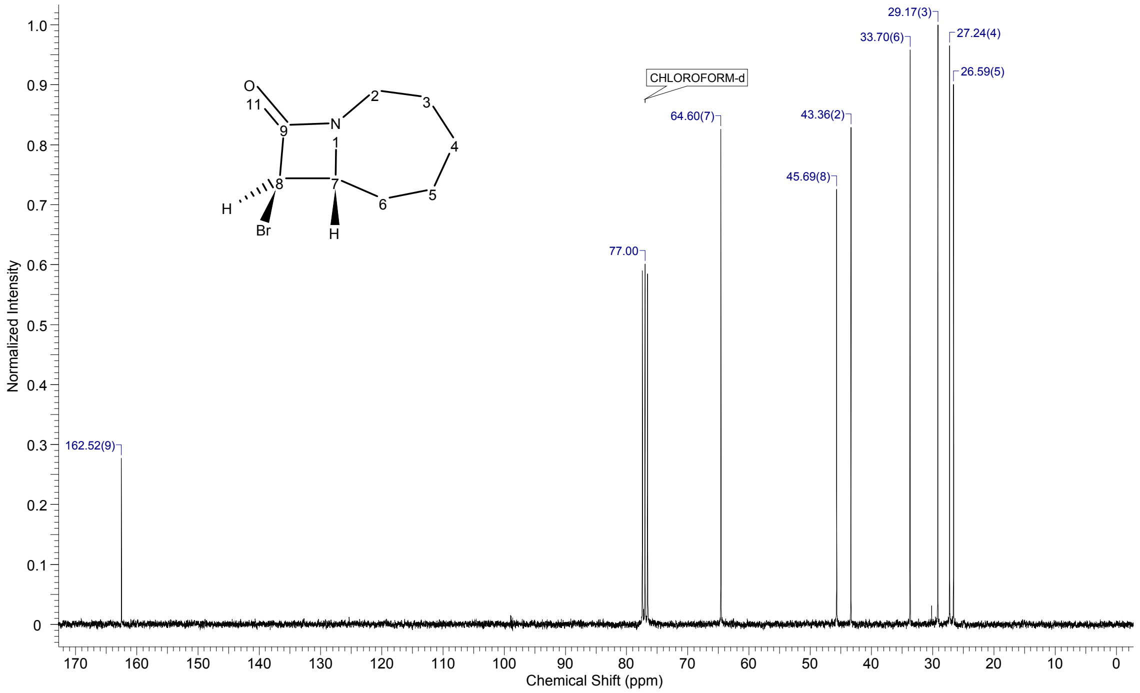

Acquisition Time (sec)	1.8219	***Frequency (MHz)***	75.48	***Nucleus***	13C	***Origin***	Bruker	***Original Points Count***	32768	***Points Count***	65536
Pulse Sequence	DEPT135			***Spectrum Offset (Hz)***	7536.2251			***Spectrum Type***	DEPT135		
Sweep Width (Hz)	17985.61										

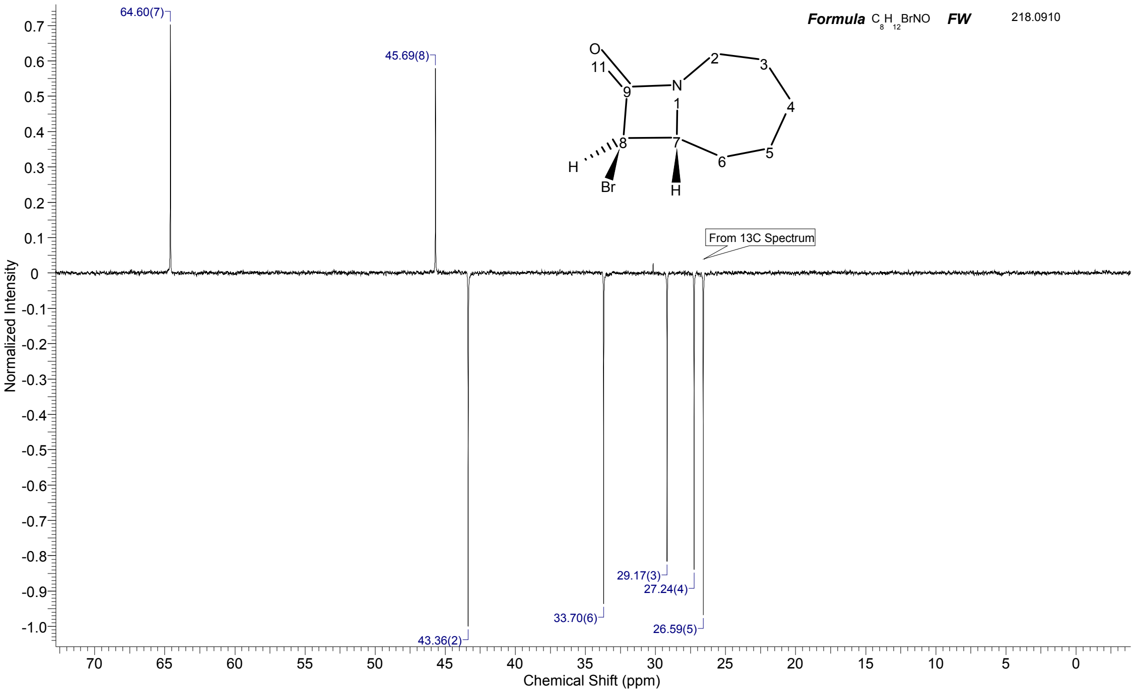

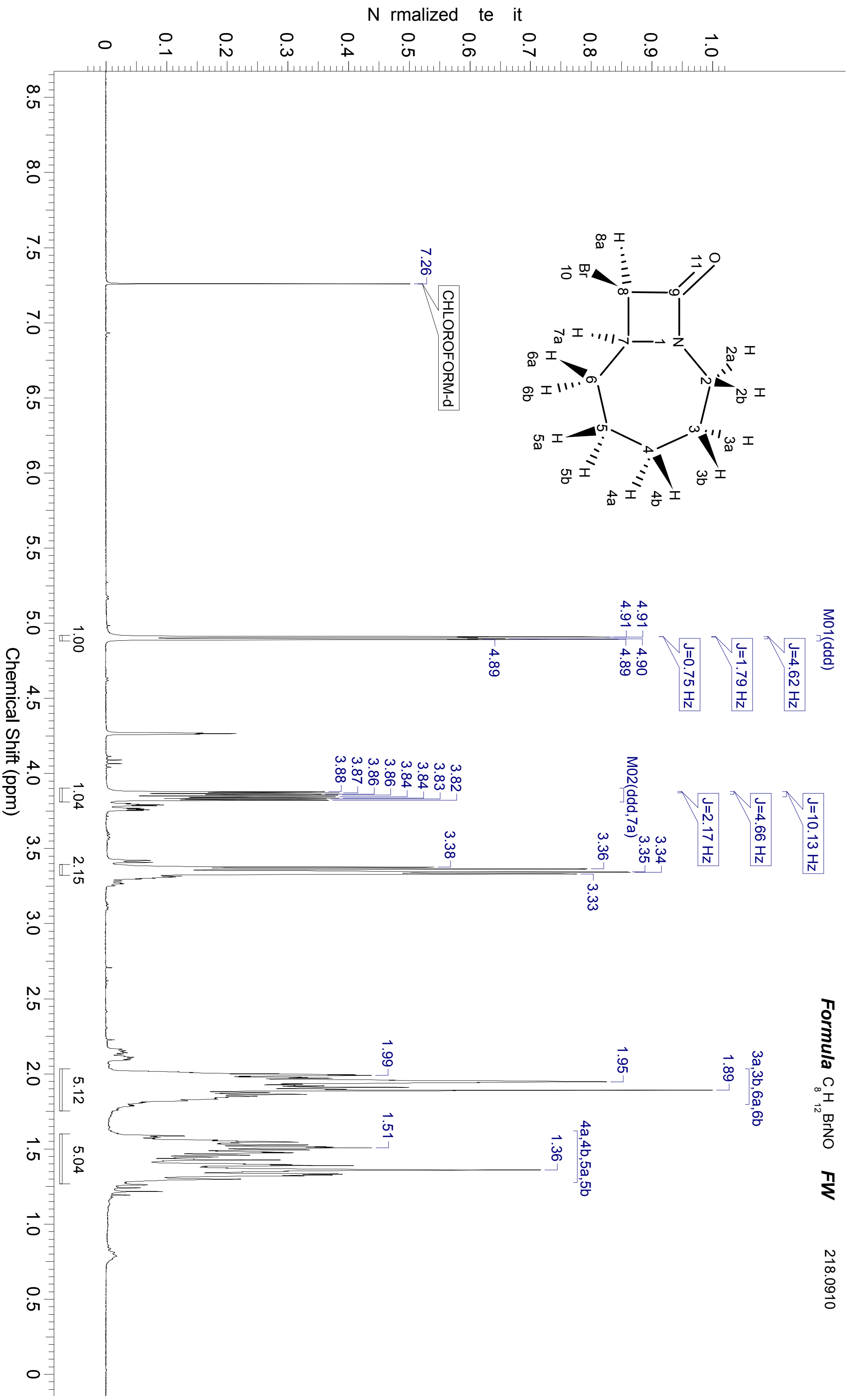

Acquisition Time (sec)	5.3084	Comment	5 mm QNP 1H/15N/13C/31P Z08011/0020	Frequency (MHz)	300.13	Nucleus	1H
Number of Transients	16	Origin	DPX300	Original Points Count	32768	Points Count	32768
Pulse Sequence	zg30	Receiver Gain	143.70	SW(cyclical) (Hz)	6172.84	Solvent	CHLOROFORM-d
Spectrum Offset (Hz)	1847.1124	Spectrum Type	STANDARD	Sweep Width (Hz)	6172.65	Temperature (degree C)	26.160

endo-8-bromo-1-azabicyclo[5.2.0]nonan-9-one (*endo-***5c**) – ^{13}C-NMR & DEPT-135

Acquisition Time (sec)	1.8219	***Comment***	C13CPD CDCl3 D: {UIO\aasmuk} 1			***Frequency (MHz)***	75.48	***Nucleus***	13C
Number of Transients	512	***Origin***	DPX300	***Original Points Count***	32768	***Points Count***	32768	***Pulse Sequence***	zgpg30
Receiver Gain	10321.30	***SW(cyclical) (Hz)***	17985.61	***Solvent***	CHLOROFORM-d			***Spectrum Offset (Hz)***	7538.6401
Spectrum Type	STANDARD			***Sweep Width (Hz)***	17985.06	***Temperature (degree C)***	25.160		

Formula $C_8H_{12}BrNO$ ***FW*** 218.0910

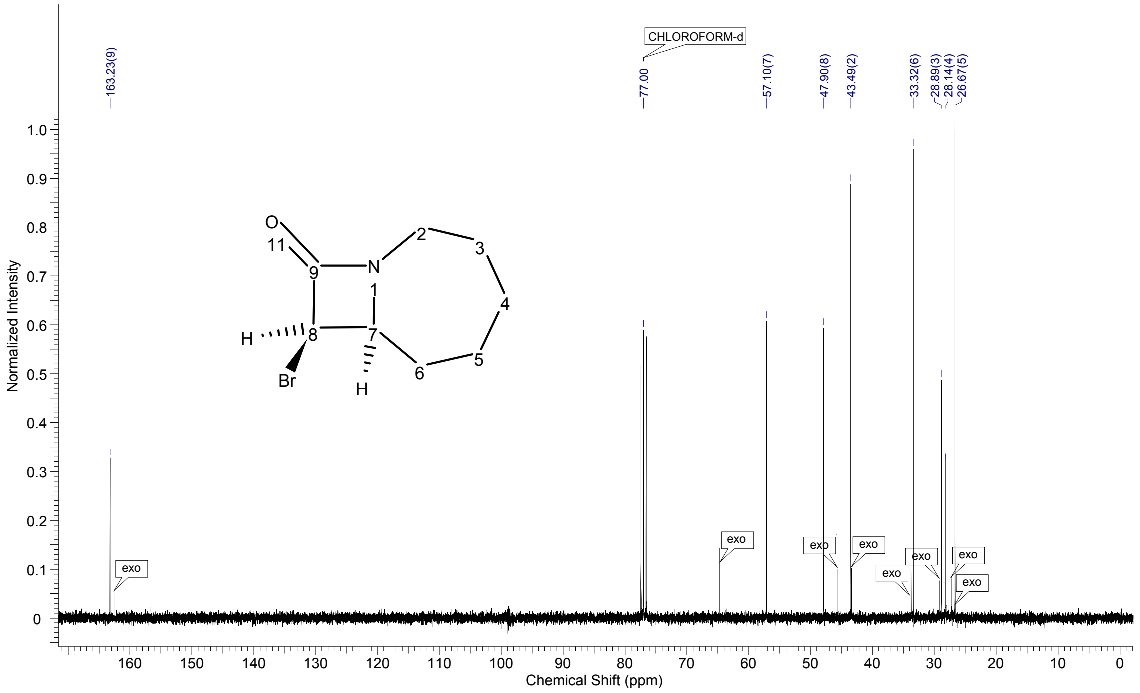

Acquisition Time (sec)	1.8219	***Comment***	C13DEPT135 CDCl3 D: {UIO\aasmuk} 1			***Frequency (MHz)***	75.48	***Nucleus***	13C
Number of Transients	256	***Origin***	DPX300	***Original Points Count***	32768	***Points Count***	32768	***Pulse Sequence***	dept135
Receiver Gain	16384.00	***SW(cyclical) (Hz)***	17985.61	***Solvent***	CHLOROFORM-d			***Spectrum Offset (Hz)***	7538.7485
Spectrum Type	DEPT135	***Sweep Width (Hz)***	17985.06	***Temperature (degree C)***	25.160				

Formula $C_8H_{12}BrNO$ ***FW*** 218.0910

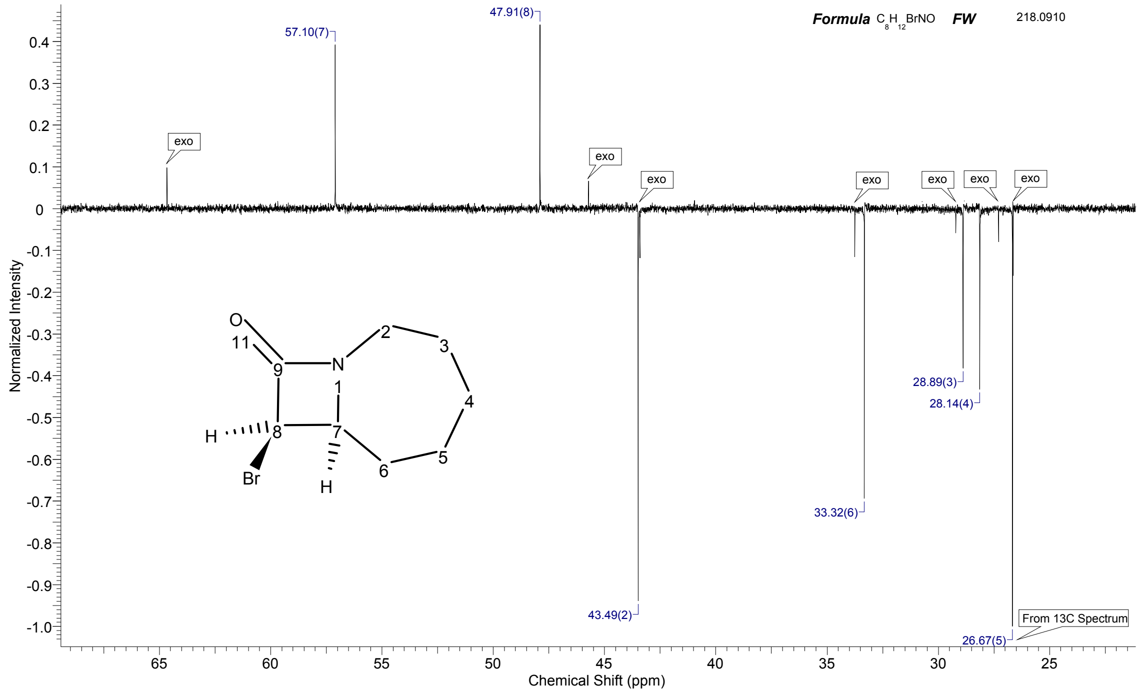

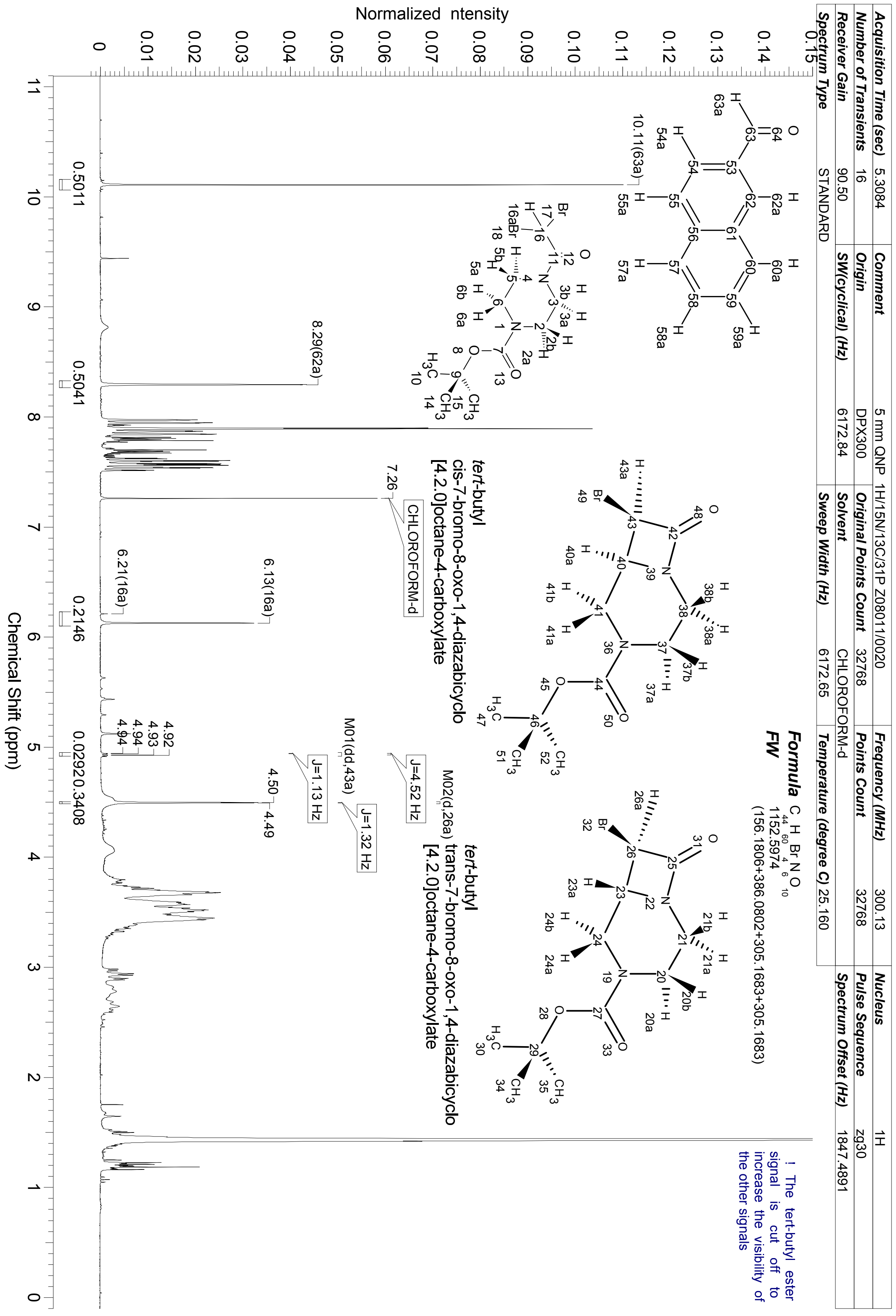
Acquisition Time (sec) 5.3084
Comment 5 mm QNP 1H/15N/13C/31P Z08011/0020
Frequency (MHz) 300.13
Nucleus 1H
Number of Transients 16
Origin DPX300
Original Points Count 32768
Points Count 32768
Pulse Sequence zg30
Receiver Gain 90.50
SW(cyclical) (Hz) 6172.84
Solvent CHLOROFORM-d
Spectrum Offset (Hz) 1847.4891
Spectrum Type STANDARD
Sweep Width (Hz) 6172.65
Temperature (degree C) 25.160
Formula C44 H60 Br4 N6 O10
FW 1152.5974
(156.1806+386.0802+305.1683+305.1683)
tert-butyl cis-7-bromo-8-oxo-1,4-diazabicyclo[4.2.0]octane-4-carboxylate
tert-butyl trans-7-bromo-8-oxo-1,4-diazabicyclo[4.2.0]octane-4-carboxylate
! The tert-butyl ester signal is cut off to increase the visibility of the other signals
10.11(63a)
8.29(62a)
7.26
CHLOROFORM-d
6.21(16a)
6.13(16a)
4.92
4.93
4.94
4.94
M01(dd,43a)
J=1.13 Hz
J=4.52 Hz
M02(d,26a)
J=1.32 Hz
4.50
4.49
0.5011
0.5041
0.2146
0.0292
0.3408
Normalized Intensity
Chemical Shift (ppm)

Acquisition Time (sec)	5.3084	***Comment***	5 mm QNP 1H/15N/13C/31P Z08011/0020	***Frequency (MHz)***	300.13	***Nucleus***	1H
Number of Transients	16	***Origin***	DPX300	***Original Points Count***	32768	***Points Count***	32768
Pulse Sequence	zg30	***Receiver Gain***	128.00	***SW(cyclical) (Hz)***	6172.84	***Solvent***	CHLOROFORM-d
Spectrum Offset (Hz)	1847.3009	***Spectrum Type***	STANDARD	***Sweep Width (Hz)***	6172.65	***Temperature (degree C)***	25.160

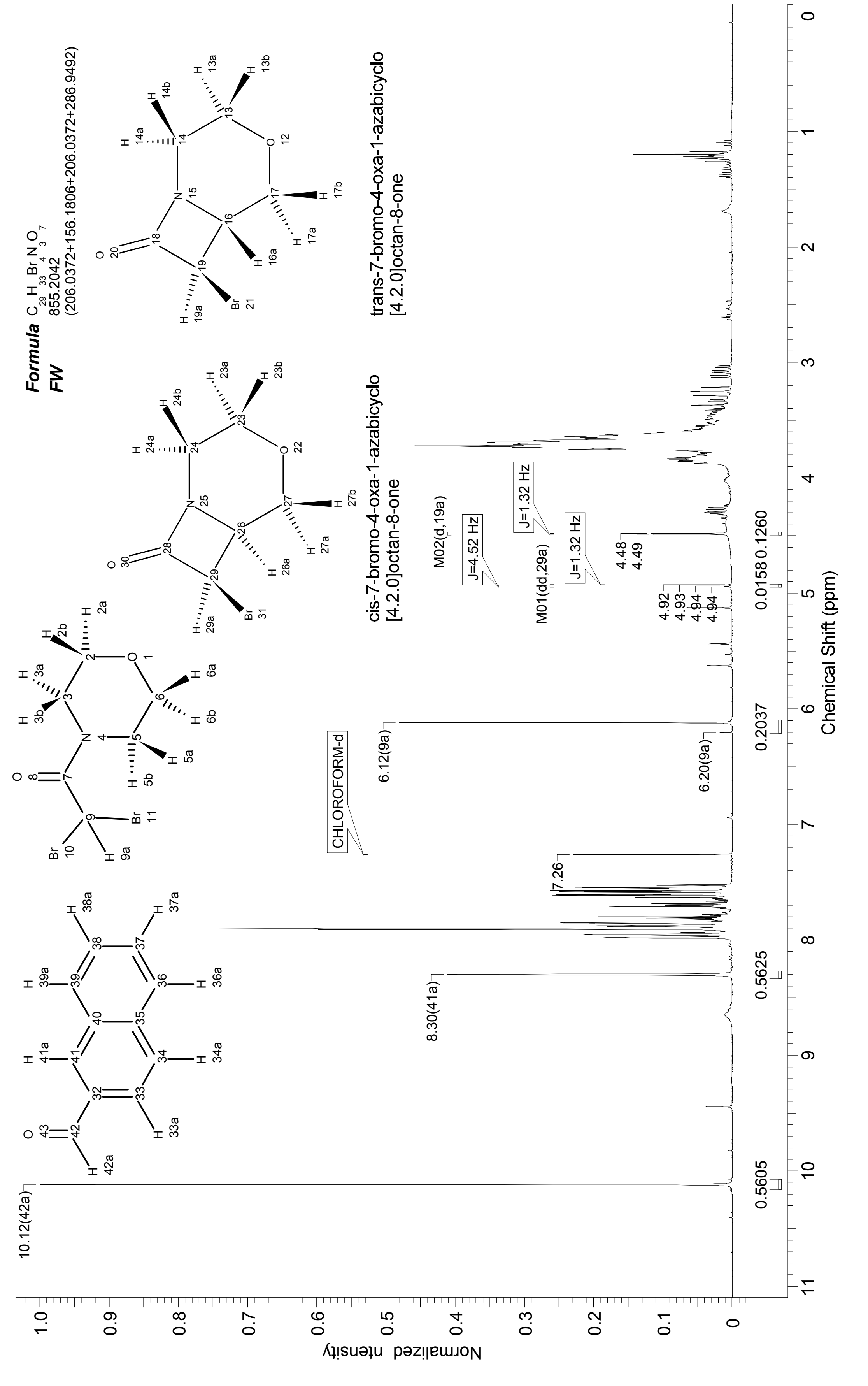

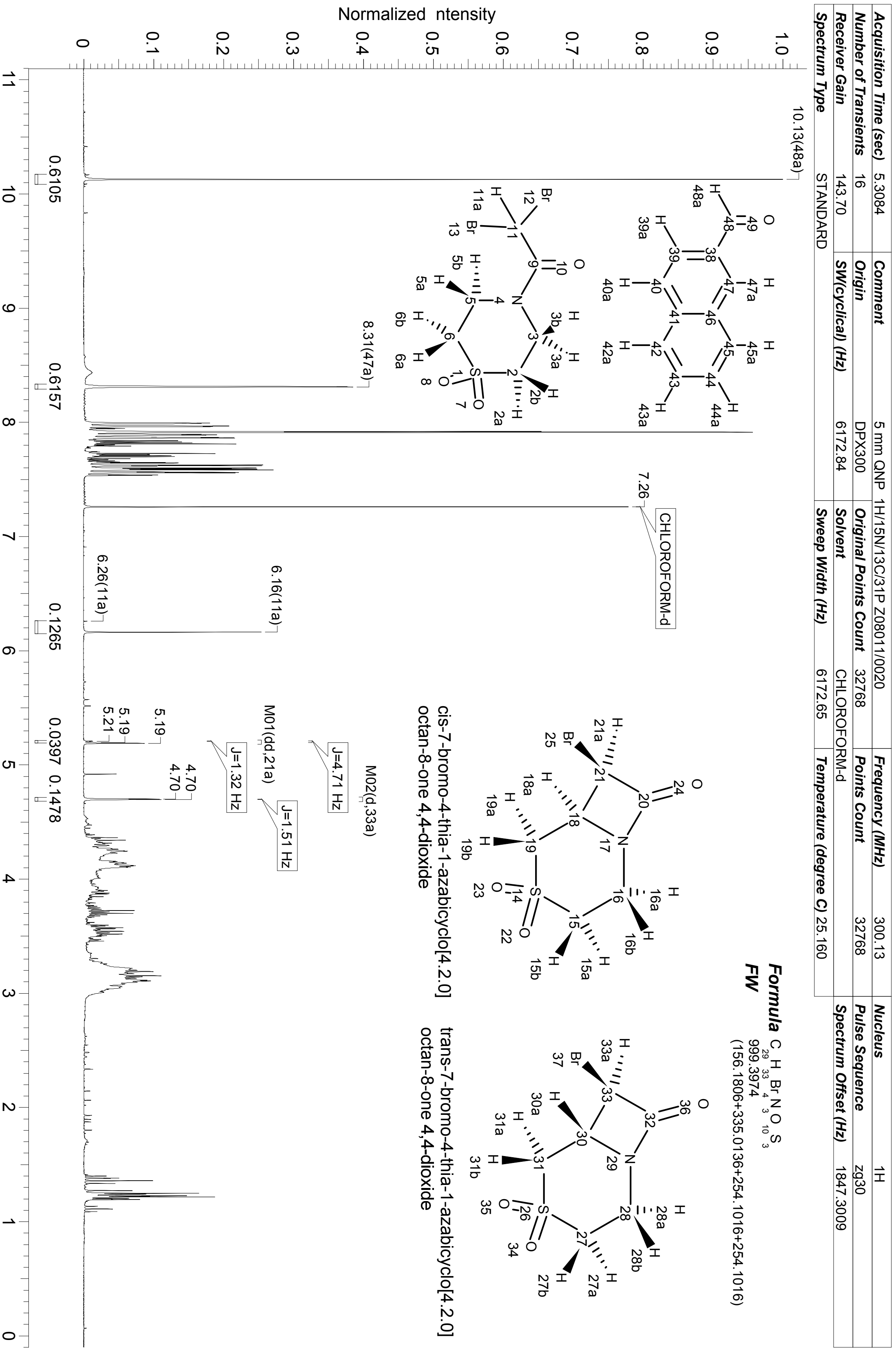
Acquisition Time (sec) 5.3084
Comment 5 mm QNP 1H/15N/13C/31P Z08011/0020
Frequency (MHz) 300.13
Nucleus 1H
Number of Transients 16
Origin DPX300
Original Points Count 32768
Points Count 32768
Pulse Sequence zg30
Receiver Gain 143.70
SW(cyclical) (Hz) 6172.84
Solvent CHLOROFORM-d
Spectrum Offset (Hz) 1847.3009
Spectrum Type STANDARD
Sweep Width (Hz) 6172.65
Temperature (degree C) 25.160
Formula C29H33Br4N3O10S3
FW 999.3974
(156.1806+335.0136+254.1016+254.1016)
cis-7-bromo-4-thia-1-azabicyclo[4.2.0]octan-8-one 4,4-dioxide
trans-7-bromo-4-thia-1-azabicyclo[4.2.0]octan-8-one 4,4-dioxide
10.13(48a)
8.31(47a)
7.26
CHLOROFORM-d
6.16(11a)
6.26(11a)
5.19
5.19
5.21
M01(dd,21a)
J=1.32 Hz
J=1.51 Hz
M02(d,33a)
J=4.71 Hz
4.70
4.70
0.6105
0.6157
0.1265
0.0397
0.1478
Normalized Intensity
Chemical Shift (ppm)

**Acquisition Time (sec)**	5.3084	**Comment**	PROTON CDCl3 D: {UIO\aasmuk} 12			**Frequency (MHz)**	300.13	**Nucleus**	1H
**Number of Transients**	16	**Origin**	DPX300	**Original Points Count**	32768	**Points Count**	32768	**Pulse Sequence**	zg30
**Receiver Gain**	114.00	**SW(cyclical) (Hz)**	6172.84	**Solvent**	CHLOROFORM-d			**Spectrum Offset (Hz)**	1846.7491
**Spectrum Type**	STANDARD			**Sweep Width (Hz)**	6172.65	**Temperature (degree C)**	25.160		

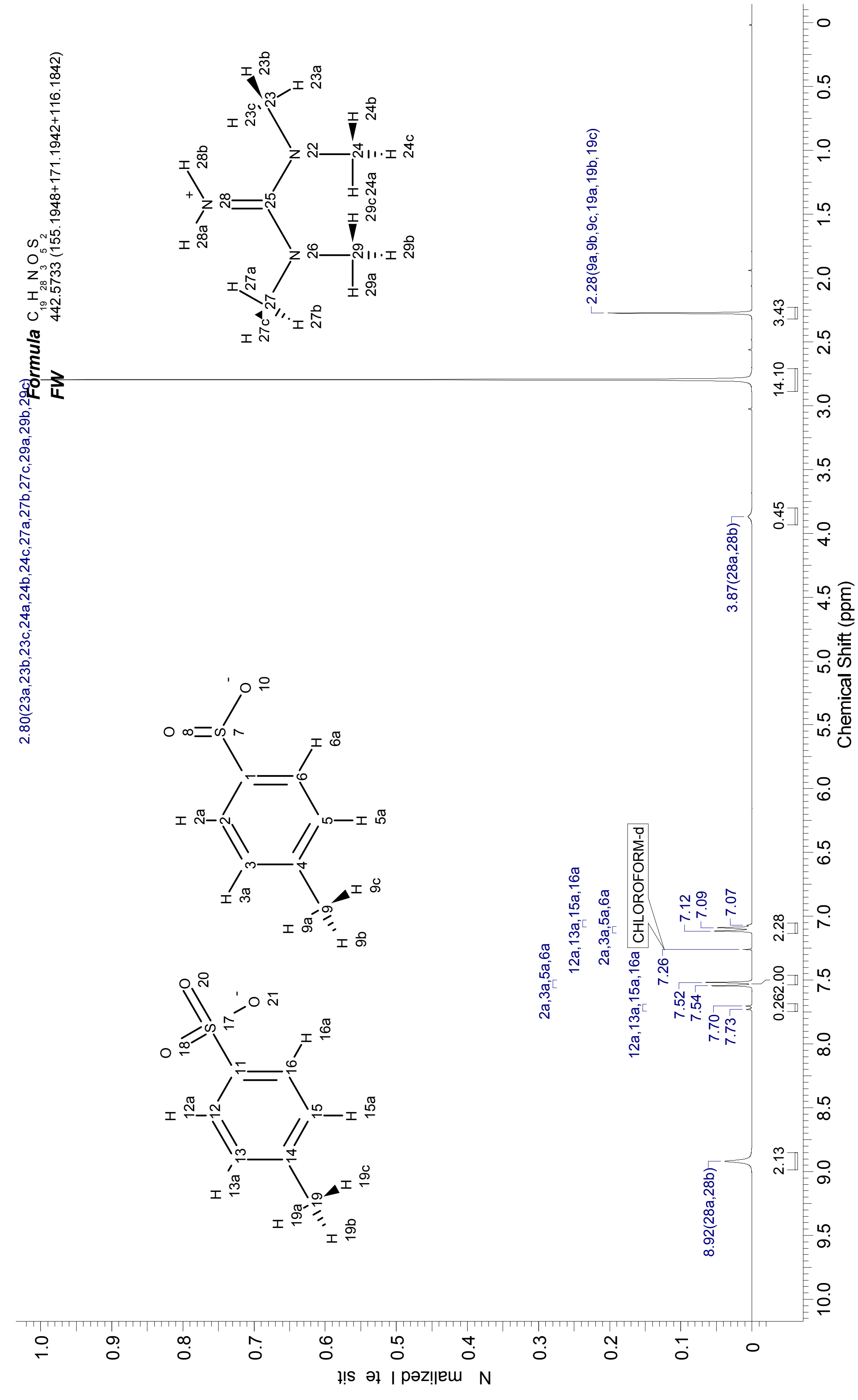

**Acquisition Time (sec)**	2.9999	**Frequency (MHz)**	200.13	**Nucleus**	1H	**Number of Transients**	16	**Origin**	DPX200
**Original Points Count**	12417	**Points Count**	16384	**Pulse Sequence**	zg30	**Receiver Gain**	2580.30	**SW(cyclical) (Hz)**	4139.07
**Solvent**	CHLOROFORM-d			**Spectrum Offset (Hz)**	1227.2200			**Spectrum Type**	STANDARD
**Sweep Width (Hz)**	4138.82	**Temperature (degree C)**	25.160						

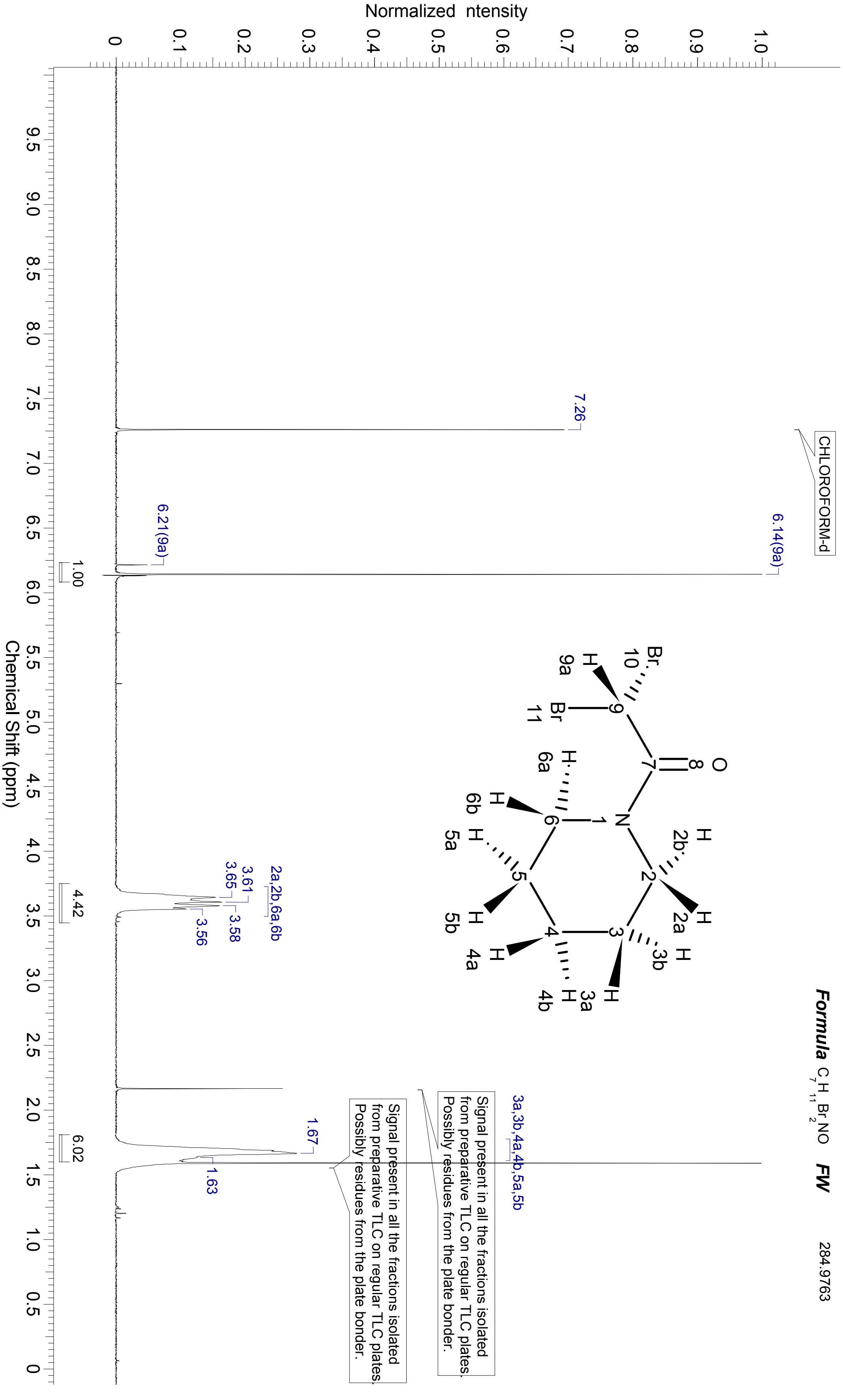

Supplement: File 1 — Detailed experimental procedures and physical data for the obtained products. [file Beilstein_J_Org_Chem-09-1407-s001.pdf]
